# Design, Synthesis, and Biological Evaluation of Naphthoquinone Salts as Anticancer Agents

**DOI:** 10.3390/molecules30091938

**Published:** 2025-04-27

**Authors:** Yao Cheng, Tsz Tin Yu, Ellen M. Olzomer, Kyle L. Hoehn, Frances L. Byrne, Naresh Kumar, David StC Black

**Affiliations:** 1 School of Chemistry, University of New South Wales, Sydney, NSW 2052, Australia; yao.cheng@unsw.edu.au (Y.C.); tsztin.yu@unsw.edu.au (T.T.Y.); 2 School of Biotechnology and Biomolecular Sciences, University of New South Wales, Sydney, NSW 2052, Australia; e.olzomer@unsw.edu.au (E.M.O.); k.hoehn@unsw.edu.au (K.L.H.)

**Keywords:** naphthoimidazole, anticancer, glycolysis, selectivity

## Abstract

The Warburg effect, a unique glycolytic phenomenon in cancer cells, presents a promising target for developing selective anticancer agents. Previously, **BH10**, a hit compound disrupting glycolytic metabolism, was identified via phenotypic screening, with Kelch-like ECH-associated protein 1 (Keap1) proposed as a potential target. To enhance its potency and selectivity, a library of **BH10**-derived salt compounds was synthesized. Among these, **7b** exhibited nanomolar anticancer activity (IC_50_ = 22.97 nM) and a high selectivity ratio (IC_50_ of non-cancerous cells/IC_50_ of cancer cells = 41.43). Molecular docking revealed that all naphthoimidazole salt analogues (**7a**–**f**) bind to Keap1 via carbonyl-mediated interactions, with variations in hydrogen-bonding residues (e.g., VAL606, ILE559).

## 1. Introduction

To sustain life, all organisms on Earth require a continuous energy supply, with glucose serving as the primary energy source for most species, including humans [1]. Energy metabolism differs between normal and cancer cells. In normal cells, glucose is transported into the cytoplasm and converted into pyruvate, generating two molecules of adenosine triphosphate (ATP) [2,3]. Under aerobic conditions, pyruvate is preferentially oxidized into CO_2_ in mitochondria, producing 36 ATP molecules. However, under hypoxic conditions, pyruvate is converted into lactate with the production of two ATP molecules [4,5]. In contrast, after glucose is converted into pyruvate in cancer cells, regardless of oxygen availability, pyruvate is primarily diverted into lactate, yielding only four ATP molecules. This phenomenon is known as the Warburg effect and is characterized by an increased rate of glycolysis followed by lactic acid fermentation, even in the presence of sufficient oxygen for oxidative phosphorylation. The Warburg effect plays a crucial role in the metabolic reprogramming of cancer cells, enabling rapid growth and proliferation [6,7]. By prioritizing glycolysis, cancer cells rapidly generate ATP and produce essential metabolic intermediates for the synthesis of macromolecules, including nucleotides, amino acids, and lipids, which support cancer cell growth and division [8]. Furthermore, the acidic microenvironment created by lactic acid production may facilitate cancer cell invasion and immune evasion [9]. Thus, the Warburg effect underscores the potential of targeting cancer-specific glycolysis as a promising therapeutic strategy.

**Figure 1:**
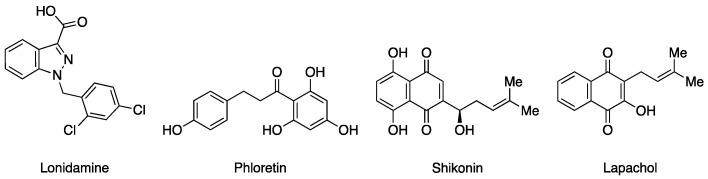
Representatives of reported quinone glycolysis inhibitors.

To date, numerous glycolysis inhibitors have been developed, particularly quinone-based compounds such as lonidamine, phloretin, shikonin, and lapachol, which interfere with key metabolic pathways essential for cancer cell proliferation (Figure 1) [10,11,12,13,14,15]. These agents have demonstrated promising in vitro and in vivo biological activity, commonly measured by their half-maximal inhibitory concentration (IC_50_) values. Low IC_50_ values indicate strong potency, which is the amount needed to produce a certain biological effect (in this case, cytotoxicity and cell death). However, these values do not always guarantee effective treatment, especially in the absence of sufficient selectivity [16,17,18]. Selectivity refers to how well a compound can target cancer cells while sparing normal cells. This ability is important when it comes to evaluating how effective the treatment can be. In anticancer drug development, high potency must be complemented by high selectivity to minimize off-target effects and unwanted side effects [19,20]. Traditional chemotherapeutics often display potent cytotoxicity (low IC_50_ values) but poor selectivity, leading to various adverse effects such as immunosuppression, alopecia, and organ toxicity [21]. In contrast, selective agents can achieve comparable or improved anticancer effects at lower doses by sparing healthy cells, thereby reducing dose-limiting toxicities and improving patient outcomes [22,23,24]. In addition, compounds that have both high potency and selectivity are less likely to lead to resistance, as they create a more intense selective pressure on cancer-specific pathways [25,26]. Such agents are also more amenable to combination therapies with immunotherapies, targeted agents, or radiotherapy, without exacerbating body toxicity [27,28]. Therefore, the optimization of both potency (reflected by IC_50_) and selectivity remains a central goal in the rational design of glycolysis-targeting anticancer agents [29].

**Figure 2:**
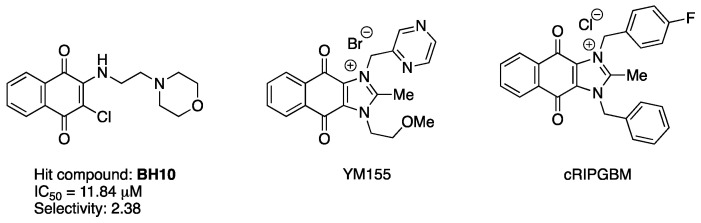
Chemical structure of hit compound and some highly potent anticancer agent.

Recently, we identified a hit compound, **BH10**, from over 5000 compounds [30]. **BH10** is a quinone molecule with a simple structure that has demonstrated promising potency (IC_50_ = 11.84 µM) against human endometrial cancer cells (HEC1A) (Figure 2). However, its selective toxicity towards cancer cells (2.38 = IC_50_ value in non-cancerous cells/IC_50_ value of cancer cells) is limited [30]. Mechanistic studies showed that **BH10** targets the Kelch-like ECH-associated protein (Keap1), induces mitochondrial oxidative stress, and reduces the activity of the redox-sensitive enzyme mitochondrial aconitase (mACN), ultimately causing the accumulation of citrate and inhibiting glycolysis in cancer cells [30,31]. The unique characteristics of the **BH10** anticancer pathway offer a promising opportunity to investigate highly selective anticancer agents.

Given that some salt compounds, such as YM155 and cRIPGBM (Figure 2), exhibit excellent anticancer potency, we aimed to synthesize a series of salt compounds based on **BH10** to investigate their anticancer activity [32,33,34]. In the design of anticancer salt compounds, halide anions are frequently selected as counterions, particularly chloride, bromide, and iodide [35]. In this study, iodide and bromide anions were selected as the primary counterions for our salt-based anticancer compounds. This preference is due to their synthetic convenience and their ability to form stable salts under mild reaction conditions, which facilitates early-stage compound evaluation [35,36]. Overall, the main objective of this study is to determine whether these salt compounds exhibit increased potency and selectivity compared to **BH10**.

## 2. Results and Discussion

### 2.1. Chemistry

**Scheme 1:**
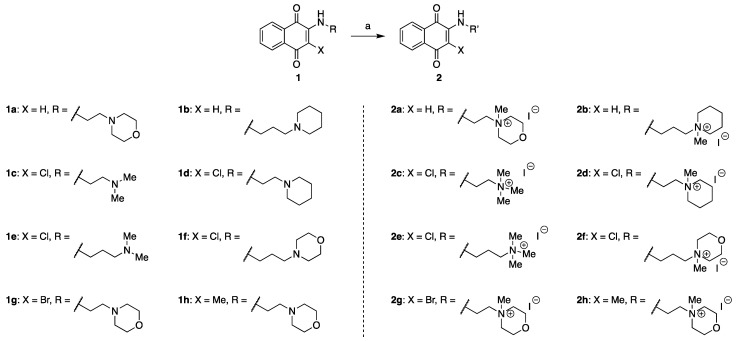
Synthesis of quinone salt analogues **2a**–**h**. Reagents and conditions: (a) MeI, MeCN, r.t., 12 h.

**Scheme 2:**
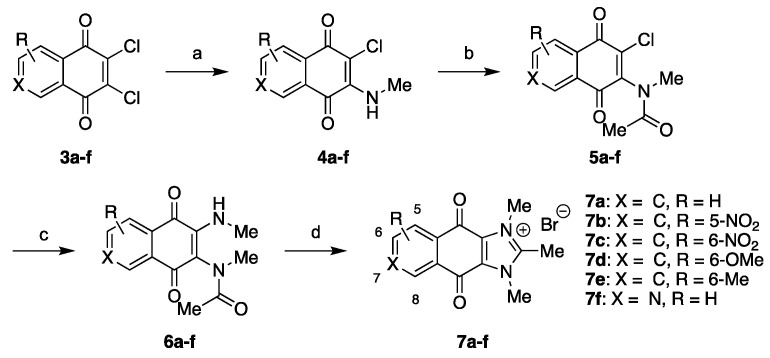
Synthesis of quinone salt analogues **7a**–**f**. Reagents and conditions: (a) NH_2_CH_3_, Et_3_N, EtOH, r.t., 18 h; (b) acetic anhydride, conc. H_2_SO_4_ (1 drop), r.t., 1.5 h; (c) NH_2_CH_3_, Et_3_N, toluene, 45 °C, 1 h; (d) 48% HBr (aq), EtOH, EtOAc, 45 °C, 4 h, r.t., overnight.

**Scheme 3:**
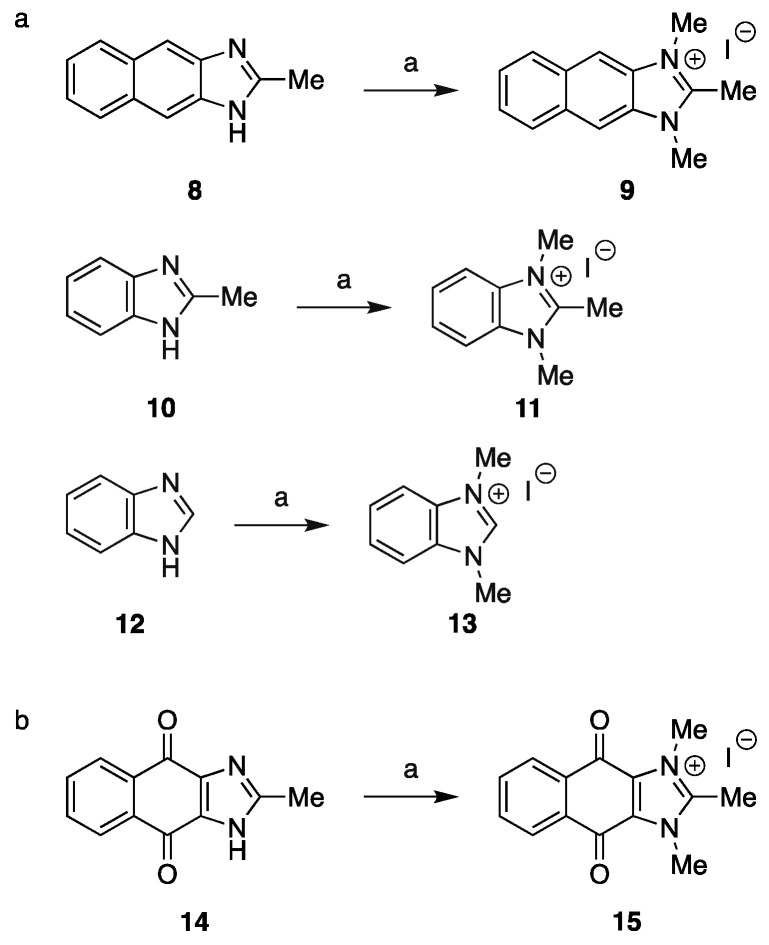
Synthesis of quinone salt analogues **9**, **11**, **13**, and **15** (**a**,**b**). Reagents and conditions: (a) MeI, 70 °C, 8 h, seal tube.

**Scheme 4:**
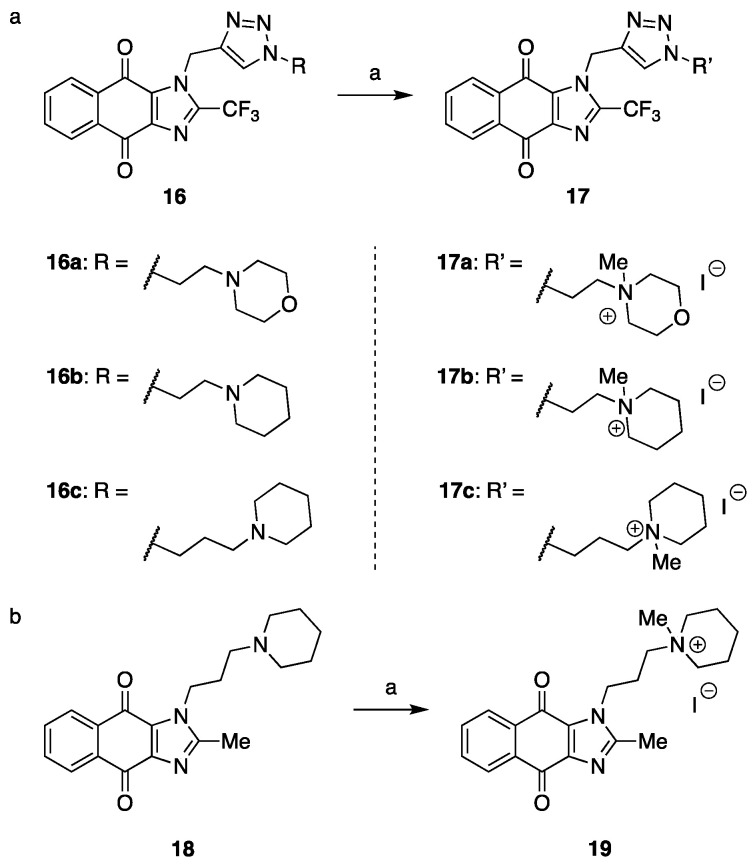
Synthesis of quinone salt analogues **17a**–**c** and **19** (**a**,**b**). Reagents and conditions: (a) MeI, MeCN, r.t., 12 h.

A library of quinone salt analogues was synthesized according to Scheme 1, Scheme 2, Scheme 3 and Scheme 4. **BH10** salt derivatives **2a**–**h** were synthesized to evaluate the impact of iodide salts (Scheme 1). Methyl iodide was selected to methylate the amine group, generating the corresponding iodide salt analogues. All derivatives were synthesized under identical reaction conditions: the corresponding starting materials were treated with excess methyl iodide in acetonitrile at room temperature for 12 h, affording the target products in similar yields. All starting materials **1a**–**h** have been reported in our earlier work [37].

Based on our previous study, the naphthoimidazole scaffold demonstrated better potency; thus, we aimed to incorporate this scaffold into salt compounds [31,37]. The dichlorononaphthoquinones **3a**–**f** underwent nucleophilic substitution with methylamine via Michael addition to yield **4a**–**f** in a yield of about 85% across all reactions. Subsequently, **4a**–**f** were reacted with acetic anhydride in the presence of concentrated sulfuric acid to form the amide intermediates **5a**–**f**. These intermediates then underwent a second Michael addition with methylamine to produce intermediates **6a**–**f**. Finally, compounds **7a**–**f** were synthesized through imidazole cyclization under hydrobromic acid, achieving yield of about 50% (Scheme 2).

Additionally, 1*H*-benzo[*d*]imidazole derivatives (**9**, **11**, and **13**) were synthesized to evaluate the impact of the removal of quinone on anticancer activity (Scheme 3). Compound **15** was synthesized to assess the effect of different counterions (iodide and bromide) on biological activity.

Furthermore, in a previous study, we introduced the triazoles to the naphthoimidazole scaffold, which showed good glycolysis-inhibiting potency [31]. Methylation of these structures was performed to generate salt compounds and evaluate the impact of the salt formation on their bioactivity (Scheme 4).

### 2.2. Structure–Activity Relationship (SAR) Study

The cytotoxic effects of the synthesized salt compounds were assessed against human endometrial cancer cells (HEC1A) and normal endometrial stromal cells (MAD11) using the MTT cell viability assay. **BH10** was used as the reference compound. The results are presented in Table 1.

**Figure 3:**
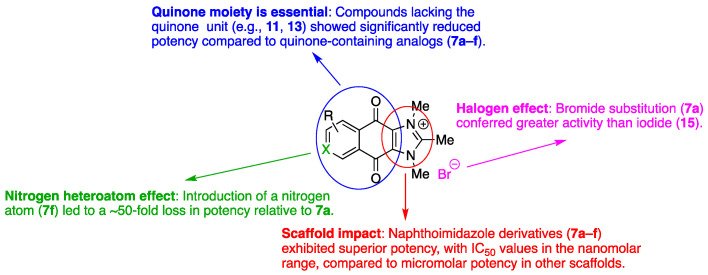
SAR of synthesized salt derivatives. The quinone moiety is essential for potency. The naphthoimidazole scaffold demonstrated remarkable potency and selectivity. However, the incorporation of a nitrogen atom into the naphthoimidazole core resulted in decreased activity. Additionally, the bromide counterion conferred greater potency than the iodide counterion.

Based on the biological characterization data of the synthesized analogues against the HEC1A and MAD11 cell lines, a structure–activity relationship (SAR) was established (Figure 3). Firstly, a series of naphthalene-1,4-dione scaffold analogues (**2a**–**h** in Scheme 1) were synthesized to investigate the effect of chain modification on bioactivity. All analogues exhibited weaker bioactivity than **BH10**, with some compounds completely losing potency. The starting materials **1a**–**h** were derived from our previous study, in which some of them demonstrated good anticancer potency (for **1b**–**g**, IC_50_: 1–20 μM, whereas for **1a** and **1h**, IC_50_ > 40 μM) [37]. The addition of a methyl group to the terminal amine, forming a salt compound, was generally detrimental to potency. However, in the case of **2a**, this modification improved potency when converting **1a** from a non-potent compound into a weakly active analogue (IC_50_ = 21.12 μM). In contrast, the same modification in **2b**–**h** resulted in a significant decrease or complete loss of potency. Therefore, the strategy of introducing a salt amine group is not suitable for naphthalene-1,4-dione scaffold compounds.

Subsequently, relative to the lead compound **BH10**, dimethylnaphthoimidazole salt compounds (**7a**–**f** in Scheme 2) exhibited significantly enhanced potency against cancer cells. These compounds consistently demonstrated nanomolar-range IC_50_ values, with compound **7a** displaying the highest activity at an IC_50_ of 9.53 nM. Upon introducing an electron-withdrawing group (–NO₂) at the 5-position of the benzene ring (**7b**), potency decreased to 22.97 nM; however, selectivity markedly increased to 41.43, representing the best performance observed in this study. Interestingly, although the introduction of –NO₂ at the 6-position (**7c**) resulted in a potency comparable to that of **7b**, its selectivity declined sharply to 2.37, aligning with that of **BH10**. Additionally, the incorporation of electron-donating groups at the benzene ring, 6-OMe in **7d** and 7-Me in **7e**, had a minimal impact, yielding IC_50_ values of 25.62 and 14.92 nM, respectively, with both compounds exhibiting similar selectivity of approximately 7. Furthermore, the introduction of a nitrogen heteroatom at the 7-position of the benzene ring resulted in compound **7f**, which displayed significantly lower potency (IC_50_ = 472.4 nM) and a selectivity of 10.16. Among the compounds investigated, **7b** emerged as the most promising candidate, as it exhibited the highest selectivity despite not possessing the greatest potency. These findings indicate that among salt compounds, the naphthoimidazole scaffold is intrinsically more potent than the 1,4-naphthoquinone scaffold.

In addition, benzimidazole salt compounds (**9**, **11**, and **13**) were synthesized to assess the effect of removing the quinone moiety from the scaffold. Biological testing revealed that compounds **11** and **13** completely lost biological activity (IC_50_ > 100 µM), while the potency of compound **9** was also significantly reduced, with an IC_50_ of 24.88 µM. Given that compounds **7a**–**f** are bromide salts, and the remaining compounds are iodide salts, compound **15** was synthesized to investigate the effect of counterion variation. Comparison of **7a** (bromide) and **15** (iodide) revealed that compound **15** exhibited slightly reduced potency (IC_50_ = 13.47 nM) but marginally higher selectivity (11.43). These results indicate that counterions could influence the biological activity and selectivity of these compounds to a limited extent. Counterion optimization may be essential for clinical translation, and in future studies, chloride salts will be introduced to assess their effects on biocompatibility [35,38]. This direction will also allow us to further investigate the effects of counterion identity on pharmacokinetics, toxicity, and therapeutic efficacy [35,36].

In a previous study, naphthoimidazole analogues demonstrated promising activity. To further investigate the impact of becoming salt compounds, several naphthoimidazole analogues, **16a**–**c** (**16a** with IC_50_ of 18.11 µM and selectivity of 1.73; **16b** with IC_50_ of 6.84 µM and selectivity of 2.01; **16c** with IC_50_ of 2.60 µM and selectivity of 2.77) and **18** (with IC_50_ of 6.40 µM and selectivity of 3.59), were chosen to be converted into salt derivatives [31]. Upon converting these analogues into their corresponding salt, none of these derivatives (**17a**–**c**, and **19**) showed significant change in potency, but all of them showed a significant improvement in selectivity, especially for **17a**–**c**, in which the selectivity increased by 5.74, 7.33, and 6.84 times, whilst the selectivity also improved by 1.79 times for **19**. Overall, comparing these salt compounds with their previous corresponding parent compounds, salt compounds show higher potential for anticancer drug development.

Based on the biotesting results and subsequent analysis, several structure–activity relationships were identified (Figure 3). Firstly, the quinone moiety played a pivotal role in determining potency. Secondly, the naphthoimidazole scaffold exhibited a significant enhancement in both potency and selectivity. Additionally, the incorporation of a nitrogen atom into the naphthoimidazole core led to a reduction in biological activity. Lastly, the bromide counterion exhibited superior potency compared to the iodide counterion.

To further highlight the potential of compound **7b**, a comparative analysis was performed against other reported glycolysis-targeting and ionic anticancer agents, including lonidamine, phloretin, shikonin, lapachol, cRIPGBM, and YM155, as listed in Table S1 [31,33,39,40,41,42,43,44,45,46,47,48,49]. These compounds represent diverse structural classes and mechanisms of action. Notably, compound **7b** exhibited a nanomolar IC_50_ value (22.97 nM) and an exceptional selectivity index (41.43), surpassing most classical inhibitors in both potency and selectivity. YM155 and cRIPGBM also displayed nanomolar potency, although their selectivity indices are comparatively lower.

### 2.3. Molecular Docking Study

**Figure 4:**
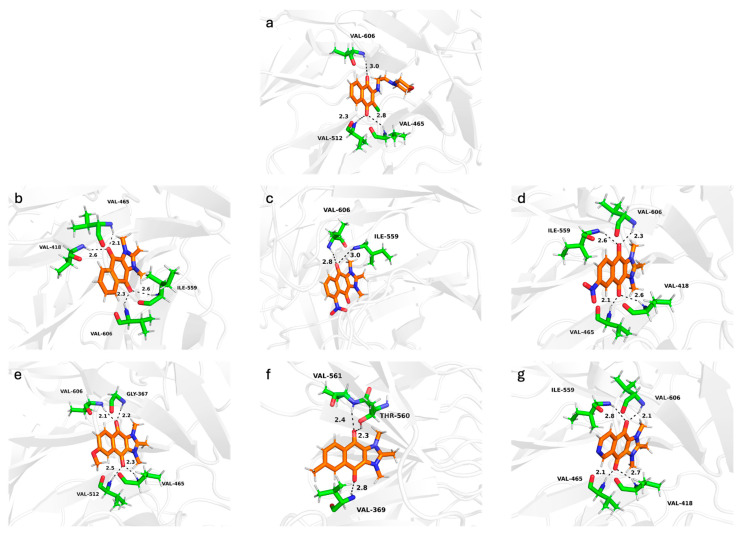
Predicted binding interactions of **BH10** (**a**) and **7a**–**f** (**b**–**g**) with Keap1 (PDB code: 4XMB). Black dashed lines indicate hydrogen bonding interactions between the ligands and the target protein. (**a**) The carbonyl group of **BH10** forms hydrogen bonds with VAL-465, VAL-512, and VAL-606 (docking score: −5.805 kcal/mol). Similarly, the six potent naphthoimidazole salt analogues bind within the same hydrophobic pocket: (**b**) **7a** interacts with VAL-418, VAL-465, ILE-559, and VAL-606 (docking score: −8.207 kcal/mol); (**c**) **7b**, with ILE-559 and VAL-606 (docking score: −7.703 kcal/mol); (**d**) **7c**, with VAL-418, VAL-465, ILE-559, and VAL-606 (docking score: −8.828 kcal/mol); (**e**) **7d**, with GLY-367, VAL-465, VAL-512, and VAL-606 (docking score: −8.449 kcal/mol); (**f**) **7e** with VAL-369, THR-560, and VAL-561 (docking score: −8.842 kcal/mol); (**g**) and **7f**, with VAL-418, VAL-465, ILE-559, and VAL-606 (docking score: −8.488 kcal/mol).

Given the potent anticancer activity of the naphthoimidazole salt analogues (**7a**–**f**), a molecular docking simulation was conducted to evaluate their interactions with the target protein (Figure 4). Docking was performed using Maestro 14.1, with Keap1 (PDB: 4XMB) treated as a rigid receptor and all ligands modelled as flexible, using the Standard Precision (SP) protocol. The docking was carried out at the native ligand-binding pocket of Keap1, as defined by the co-crystallized ligand. **BH10**, the reference compound, was included for comparison. All salt analogues interacted with the same hydrophobic pocket as **BH10**, primarily through their carbonyl groups. **BH10** formed hydrogen bonds with residues VAL-465, VAL-512, and VAL-606, and exhibited a docking score of −5.805 kcal/mol. In contrast, the naphthoimidazole salts demonstrated significantly stronger predicted binding affinities, with docking scores ranging from −7.703 to −8.842 kcal/mol, consistent with their enhanced biological potency. Among these, **7b** showed nanomolar anticancer activity (IC_50_ = 22.97 nM) and the highest selectivity (41.43). Docking analysis revealed that **7b** formed key hydrogen bonds with ILE-559 and VAL-606, suggesting that these interactions may contribute to its enhanced selectivity. Although **7b** did not exhibit the most favorable docking score (−7.703 kcal/mol), the positioning of its nitro group at the 5-position may influence its binding orientation and reduce non-specific interactions, potentially explaining its superior selectivity despite a slightly lower predicted affinity. Other analogues, such as **7c** and **7e**, exhibited higher docking scores (−8.828 and −8.842 kcal/mol, respectively) but displayed relatively lower selectivity compared to **7b**. This suggests that although a general correlation exists between docking energy and biological potency, selectivity may also depend on specific molecular interactions or steric factors not captured by docking simulations.

## 3. Experimental

### 3.1. Chemistry

#### 3.1.1. General Information

All starting materials, solvents, and reagents were reagent grade, commercially obtained, and used as received without further purification. Reaction progress was monitored using thin-layer chromatography (TLC) on Merck 60-F254 (20–25 μm, Merck, Darmstadt, Germany) silica gel plates, visualized under UV light. Flash chromatography was performed using 200–300 mesh silica gel, with detection under UV light at 254 and 284 nm. ^1^H NMR and ^13^C NMR spectra were recorded on Bruker ARX-400 spectrometers (Bruker, Singapore) using CDCl_3_ or DMSO-*d*_6_ as solvents, with tetramethylsilane (TMS) as the internal standard. Low-resolution mass spectra were acquired in ESI mode using an Elite mass spectrometer. High-resolution mass spectrometry (ESI-MS) was performed by the Bioanalytical Mass Spectrometry Unit at UNSW.

**General procedure A**: A flask was loaded with **BH10** analogues (**1a**–**h**) (0.2 mmol, 1.0 equiv.), acetonitrile (5.0 mL), and MeI (0.4 mmol, 2.0 equiv.). The reaction was stirred at room temperature for 12 h under a nitrogen atmosphere. The solvent was then evaporated, the residue filtered, and the precipitate washed with EtOAc under vacuum to afford salt compounds **2a**–**h**.

**General procedure B**: The corresponding dichloronaphthoquinone (**3a**–**f**) (2.0 mmol, 1.0 equiv.) was dissolved in 10.0 mL of ethanol under a nitrogen atmosphere, followed by the addition of 200 μL of a 25% methylamine solution and triethylamine (6.0 mmol, 3.0 equiv.). The reaction mixture was stirred at room temperature for 18 h. The solvent was evaporated, and the resulting red residue was filtered and washed with hexane under vacuum, yielding the corresponding intermediates (**4a**–**f**).

**General procedure C**: A solution of intermediates (**4a**–**f**) (1.0 mmol, 1.0 equiv.) in acetic anhydride (1.5 mL) was prepared under a nitrogen atmosphere, and one drop of concentrated H_2_SO_4_ was added as a catalyst. The reaction mixture was stirred at room temperature for 1.5 h. Subsequently, 5 mL of water was added dropwise to quench excess acetic anhydride, with continuous stirring for an additional 5 min. The reaction mixture was then extracted with ethyl acetate (3 × 10 mL). The combined organic layers were washed with saturated NaHCO_3_ and brine, dried over anhydrous Na_2_SO_4_, and concentrated under reduced pressure. The residue was purified by silica gel column chromatography using *n*-hexane/EtOAc (4:1) as the eluent, affording the corresponding intermediates (**5a**–**f**).

**General procedure D**: The appropriate intermediates (**5a**–**f**) (0.5 mmol, 1.0 equiv.) were dissolved in 4.0 mL of toluene under a nitrogen atmosphere, followed by the dropwise addition of methylamine (0.6 mmol, 1.2 equiv.) and triethylamine (0.6 mmol, 1.2 equiv.). The reaction mixture was stirred at room temperature for 1 h, after which the precipitate was collected by vacuum filtration, washed with water and ethanol, and dried to afford intermediates (**6a**–**f**).

**General procedure E**: To a solution of intermediates (**6a**–**f**) (0.3 mmol, 1.0 equiv.) in EtOH/EtOAc (1:1) (2.0 mL) under a nitrogen atmosphere, 48% HBr was added dropwise. The reaction mixture was stirred at 45 °C for 4 h, then allowed to continue stirring at room temperature overnight. The solvent was then evaporated, and the crude product was purified by flash column chromatography on silica (MeOH/DCM 1:10), affording the final compounds (**7a**–**f**).

**General procedure F**: A 25 mL sealed tube was charged with imidazole starting materials (8, 10, 12, 14, 16a–c, and 18) (0.4 mmol, 1.0 equiv.) and MeI (1.5 mL). The reaction mixture was sealed and stirred at 70 °C for 8 h. The MeI was evaporated by a stream of nitrogen, and the residue was filtered and washed with EtOAc under vacuum, yielding the salt compounds (**9**, **11**, **13**, **15**, **17a**–**c**, and **19**).

#### 3.1.2. Synthesis of Intermediates

**2-Chloro-3-(methylamino)naphthalene-1,4-dione** (**4a**) [47]. Following general procedure B (2 mmol scale), **4a** (397.9 mg, 1.80 mmol, 90% yield) was obtained as a red solid. ^1^H NMR (400 MHz, CDCl_3_) *δ* 8.06 (dd, *J* = 7.6, 1.3 Hz, 1H), 7.95 (dd, *J* = 7.6, 1.3 Hz, 1H), 7.69–7.62 (m, 1H), 7.56 (td, *J* = 7.6, 1.3 Hz, 1H), 3.38 (s, 3H). ^13^C NMR (101 MHz, CDCl_3_) *δ* 180.4, 145.0, 134.9, 132.7, 132.3, 129.7, 126.7, 126.7, 32.5. MS (+ESI) calcd for C_11_H_8_ClN_2_ [M + H]^+^, 222.03; found, 222.10.

**3-Chloro-2-(methylamino)-5-nitronaphthalene-1,4-dione** (**4b**) [50]. Following general procedure B (2 mmol scale), **4b** (409.6 mg, 1.54 mmol, 77% yield) was obtained as a red solid. ^1^H NMR (400 MHz, DMSO-*d*_6_) *δ* 8.23–8.14 (m, 1H), 8.07–7.98 (m, 1H), 7.90 (t, *J* = 7.9 Hz, 1H), 7.75 (d, *J* = 5.8 Hz, 1H), 3.29 (d, *J* = 5.6 Hz, 3H). ^13^C NMR (101 MHz, DMSO-*d*_6_) *δ* 178.0, 148.2, 136.3, 134.2, 133.6, 129.1, 128.6, 128.5, 126.7, 32.8. MS (+ESI) calcd for C_11_H_8_ClN_2_O_4_ [M + H]^+^, 267.02; found, 267.10.

**3-Chloro-2-(methylamino)-6-nitronaphthalene-1,4-dione** (**4c**) [50]. Following general procedure B (2 mmol scale), **4c** (430.9 mg, 1.62 mmol, 81% yield) was obtained as a red solid. ^1^H NMR (400 MHz, CDCl_3_) *δ* 8.86 (d, *J* = 2.3 Hz, 1H), 8.56 (dd, *J* = 8.5, 2.3 Hz, 1H), 8.37 (d, *J* = 8.5 Hz, 1H), 6.24 (s, 1H), 3.52 (dd, *J* = 5.8 Hz, 3H). ^13^C NMR (101 MHz, CDCl_3_) *δ* 178.8, 150.2, 129.0, 128.6, 126.7, 121.9, 29.7. HRMS (+ESI) calcd for C_11_H_7_ClN_2_NaO_4_ [M + Na]^+^, 288.9987; found, 288.9987.

**3-Chloro-6-methoxy-2-(methylamino)naphthalene-1,4-dione** (**4d**). Following general procedure B (2 mmol scale), **4d** (411.6 mg, 1.64 mmol, 82% yield) was obtained as a red solid. MP: 226–227 °C. ^1^H NMR (400 MHz, CDCl_3_) *δ* 7.99 (d, *J* = 8.6 Hz, 1H), 7.63 (d, *J* = 2.7 Hz, 1H), 7.08 (dd, *J* = 8.6, 2.7 Hz, 1H), 6.24 (s, 1H), 3.97 (s, 3H), 3.47 (s, 3H). ^13^C NMR (101 MHz, CDCl_3_) *δ* 179.1, 165.3, 145.1, 135.4, 129.5, 129.1, 122.9, 120.9, 118.8, 110.7, 77.4, 77.0, 76.7, 56.0, 32.5. IR (ATR): ν_max_ 3332, 2943, 1665, 1590, 1572, 1516, 1442, 1350, 1300, 1270, 1245, 1081, 836, 745 cm^−1^. HRMS (+ESI) calcd for C_12_H_10_ClNO_3_ [M + H]^+^, 252.0422; found, 252.0423.

**2-Chloro-6-methyl-3-(methylamino)naphthalene-1,4-dione** (**4e**). Following general procedure B (2 mmol scale), **4e** (376.0 mg, 1.60 mmol, 80% yield) was obtained as a red solid. MP: 191–192 °C. ^1^H NMR (400 MHz, CDCl_3_) *δ* 8.16–7.77 (m, 2H), 7.57–7.39 (m, 1H), 6.13 (s, 1H), 3.46 (d, *J* = 3.4 Hz, 3H), 2.49 (d, *J* = 9.4 Hz, 3H). ^13^C NMR (101 MHz, CDCl_3_) *δ* 180.8, 180.2, 146.5, 143.3, 135.6, 133.1, 127.4, 127.2, 127.1, 127.0, 77.4, 77.0, 76.7, 32.6, 22.0. IR (ATR): ν_max_ 3315, 3298, 2938, 1677, 1589, 1563, 1512, 1415, 1306, 1259, 1132, 1071, 838, 738 cm^−1^. HRMS (+ESI) calcd for C_12_H_10_ClNNaO_2_ [M + Na]^+^, 258.0292; found, 258.0293.

**7-Chloro-6-(methylamino)isoquinoline-5,8-dione** (**4f**) [51]. Following general procedure B (2 mmol scale), **4f** (351.4 mg, 1.44 mmol, 72% yield) was obtained as a red solid. ^1^H NMR (400 MHz, CDCl_3_) *δ* 9.41 (d, *J* = 0.9 Hz, 1H), 9.00 (d, *J* = 4.9 Hz, 1H), 7.83 (dd, *J* = 5.0, 0.8 Hz, 1H), 6.05 (s, 1H), 3.49 (d, *J* = 5.8 Hz, 3H). ^13^C NMR (101 MHz, CDCl_3_) *δ* 180.4, 179.9, 156.4, 154.4, 148.7, 148.3, 119.3, 118.2. MS (+ESI) calcd for C_12_H_11_ClN_3_O_2_ [M + MeCN + H]^+^, 264.05; found, 264.10.

***N*-(3-Chloro-1,4-dioxo-1,4-dihydronaphthalen-2-yl)-*N*-methylacetamide** (**5a**) [47]. Following general procedure C (1 mmol scale), **5a** (241.6 mg, 0.92 mmol, 92% yield) was obtained as a brown solid. ^1^H NMR (400 MHz, CDCl_3_) *δ* 8.26–8.10 (m, 2H), 7.91–7.75 (m, 2H), 3.18 (s, 3H), 2.13 (s, 3H). ^13^C NMR (101 MHz, CDCl_3_) *δ* 179.7, 179.2, 177.8, 134.9, 134.7, 134.2, 133.7, 133.3, 131.5, 131.3, 127.6, 35.1, 21.9. MS (+ESI) calcd for C_13_H_11_ClNO_3_ [M + H]^+^, 264.04; found, 264.10.

***N*-(3-chloro-5-nitro-1,4-dioxo-1,4-dihydronaphthalen-2-yl)-*N*-methylacetamide** (**5b**). Following general procedure C (1 mmol scale), **5b** (200.1 mg, 0.65 mmol, 65% yield) was obtained as a yellow solid. MP: 95–96 °C. ^1^H NMR (400 MHz, DMSO-*d*_6_) δ 8.38–8.32 (m, 1H), 8.29–8.09 (m, 2H), 3.01 (s, 3H), 1.90 (s, 3H). ^13^C NMR (101 MHz, DMSO-*d*_6_) δ 176.5, 176.4, 148.3, 142.0, 136.4, 136.0, 132.7, 132.5, 128.4, 122.1, 23.3, 22.1. IR (ATR): ν_max_ 3408, 1679, 1599, 1539, 1375, 1323, 1267, 1141, 1002, 689 cm^−1^. HRMS (+ESI) calcd for C_13_H_9_ClN_2_NaO_5_ [M + Na]^+^, 331.0092; found, 331.0093.

***N*-(3-Chloro-6-methoxy-1,4-dioxo-1,4-dihydronaphthalen-2-yl)-*N*-methylacetamide** (**5d**). Following general procedure C (1 mmol scale), **5d** (222.7 mg, 0.76 mmol, 76% yield) was obtained as a yellow solid. MP: 135–136 °C. ^1^H NMR (400 MHz, DMSO-*d*_6_) *δ* 8.09–8.02 (m, 1H), 7.53 (d, *J* = 2.7 Hz, 1H), 7.46–7.42 (m, 1H), 3.97 (s, 3H), 3.03 (d, *J* = 13.6 Hz, 3H), 1.86 (s, 3H). ^13^C NMR (101 MHz, DMSO-*d*_6_) *δ* 178.2, 164.5, 131.9, 130.0, 124.6, 123.2, 121.4, 120.8, 111.4, 110.1, 102.8, 56.7, 33.2, 22.1. IR (ATR): ν_max_ 3259, 1672, 1586, 1295, 1140, 1074, 996, 745 cm^−1^. HRMS (+ESI) calcd for C_14_H_12_ClNNaO_4_ [M + Na]^+^, 316.0347; found, 316.0348.

***N*-(3-Chloro-7-methyl-1,4-dioxo-1,4-dihydronaphthalen-2-yl)-*N*-methylacetamide** (**5e**). Following general procedure C (1 mmol scale), **5e** (114.8 mg, 0.41 mmol, 41% yield) was obtained as a yellow solid. MP: 175–176 °C. ^1^H NMR (400 MHz, CDCl_3_) *δ* 8.13–8.07 (m, 1H), 8.02–7.96 (m, 1H), 7.64 (d, *J* = 7.9 Hz, 1H), 3.21 (s, 3H), 2.56 (d, *J* = 2.5 Hz, 3H), 1.96 (s, 3H). ^13^C NMR (101 MHz, CDCl_3_) *δ* 178.1, 177.6, 135.6, 131.2, 129.1, 128.0, 127.8, 21.9, 21.9. IR (ATR): ν_max_ 3258, 2919, 1659, 1592, 1501, 1372, 1288, 1240, 1135, 1038, 839, 740 cm^−1^. HRMS (+ESI) calcd for C_14_H_12_ClNNaO_3_ [M + Na]^+^, 300.0398; found, 300.0397.

***N*-Methyl-*N*-(3-(methylamino)-1,4-dioxo-1,4-dihydronaphthalen-2-yl)acetamide** (**6a**) [47]. Following general procedure D (0.5 mmol scale), **6a** (73.1 mg, 0.28 mmol, 57% yield) was obtained as a red solid. ^1^H NMR (400 MHz, CDCl_3_) *δ* 8.16 (d, *J* = 7.7, Hz, 1H), 8.09 (d, *J* = 7.7 Hz, 1H), 7.82–7.75 (m, 1H), 7.71–7.63 (m, 1H), 6.29 (s, 1H), 3.18–3.08 (m, 6H), 2.00 (s, 3H). ^13^C NMR (101 MHz, CDCl_3_) *δ* 182.2, 179.3, 172.3, 144.1, 135.4, 132.7, 132.6, 130.1, 126.8, 126.7, 118.3, 77.4, 77.1, 76.7, 37.4, 30.6, 21.9. MS (+ESI) calcd for C_14_H_15_N_2_O_3_ [M + H]^+^, 259.11; found, 259.15.

***N*-Methyl-*N*-(3-(methylamino)-5-nitro-1,4-dioxo-1,4-dihydronaphthalen-2-yl)acetamide** (**6b**). Following general procedure D (0.5 mmol scale), **6b** (60.0 mg, 0.22 mmol, 45% yield) was obtained as a red solid. MP: 170–171 °C. ^1^H NMR (400 MHz, DMSO-*d*_6_) *δ* 8.22 (dd, *J* = 6.5, 2.5 Hz, 1H), 8.10–8.00 (m, 2H), 7.92 (t, *J* = 7.9 Hz, 1H), 2.92 (s, 6H), 1.85 (s, 3H). ^13^C NMR (101 MHz, DMSO-*d*_6_) *δ* 171.9, 171.8, 163.5, 148.5, 148.1, 136.5, 134.1, 133.9, 128.9, 128.6, 126.5, 37.1, 30.4, 22.0. IR (ATR): ν_max_ 3315, 1672, 1605, 1532, 1379, 1337, 1289, 1107, 698 cm^−1^. HRMS (+ESI) calcd for C_14_H_13_N_3_NaO_5_ [M + Na]^+^, 326.0747; found, 326.0747.

***N*-(6-Methoxy-3-(methylamino)-1,4-dioxo-1,4-dihydronaphthalen-2-yl)-*N*-methylacetamide** (**6d**). Following general procedure D (0.5 mmol scale), **6d** (80.6 mg, 0.28 mmol, 56% yield) was obtained as a red solid. MP: 167–168 °C. ^1^H NMR (400 MHz, CDCl_3_) *δ* 8.07 (d, *J* = 8.6 Hz, 1H), 7.51 (d, *J* = 2.7 Hz, 1H), 7.24 (dd, *J* = 8.6, 2.7 Hz, 1H), 6.18 (d, *J* = 7.5 Hz, 1H), 3.93 (s, 3H), 3.09 (d, *J* = 5.7 Hz, 6H). ^13^C NMR (101 MHz, CDCl_3_) *δ* 182.4, 179.0, 172.3, 163.1, 143.9, 131.7, 128.9, 125.9, 121.4, 118.0, 110.3, 55.9, 37.5, 30.5, 21.9. IR (ATR): ν_max_ 3305, 2931, 1659, 1597, 1568, 1515, 1423, 1349, 1311, 1246, 1067, 1022, 756 cm^−1^. HRMS (+ESI) calcd for C_15_H_16_N_2_NaO_4_ [M + Na]^+^, 311.1002; found, 311.1002.

***N*-Methyl-*N*-(7-methyl-3-(methylamino)-1,4-dioxo-1,4-dihydronaphthalen-2-yl)acetamide** (**6e**). Following general procedure D (0.5 mmol scale), **6e** (54.4 mg, 0.20 mmol, 40% yield) was obtained as an orange solid. MP: 142–143 °C. ^1^H NMR (400 MHz, CDCl_3_) *δ* 8.02–7.94 (m, 2H), 7.46 (dd, *J* = 8.0, 1.8 Hz, 1H), 3.14–3.09 (m, 6H), 2.51 (s, 3H), 1.99 (s, 3H). ^13^C NMR (101 MHz, CDCl_3_) *δ* 181.8, 179.5, 172.3, 147.0, 144.09, 133.2, 132.6, 127.7, 127.3, 127.0, 37.4, 30.5, 22.1, 21.9. IR (ATR): ν_max_ 3312, 1669, 1585, 1571, 1515, 1423, 1313, 1079, 751 cm^−1^. HRMS(+ESI) calcd for C_15_H_16_N_2_NaO_3_ [M + Na]^+^, 295.1053; found, 295.1053.

#### 3.1.3. Synthesis of Final Compounds

**4-(2-((1,4-Dioxo-1,4-dihydronaphthalen-2-yl)amino)ethyl)-4-methylmorpholin-4-ium iodide** (**2a**). Following general procedure A (1.0 mmol scale), **2a** (304.1 mg, 0.71 mmol, 71% yield) was obtained as an orange solid. MP: 253–254 °C. ^1^H NMR (400 MHz, DMSO-*d*_6_) *δ* 8.05–7.93 (m, 2H), 7.86–7.77 (m, 2H), 7.43 (s, 1H), 4.19 (q, *J* = 6.5 Hz, 2H), 3.97 (t, *J* = 4.8 Hz, 4H), 3.76 (t, *J* = 6.4 Hz, 2H), 3.54 (t, *J* = 5.1 Hz, 4H), 3.27 (s, 3H). ^13^C NMR (101 MHz, DMSO-*d*_6_) *δ* 180.3, 175.8, 148.0, 135.2, 133.4, 131.7, 130.9, 127.2, 126.5, 63.1, 60.2, 59.8, 47.4., 38.0. IR (ATR): ν_max_ 3269, 2970, 1662, 1622, 1602, 1571, 1501, 1337, 1303, 1253, 1124, 881, 724 cm^−1^. HRMS(+ESI) calcd for C_17_H_21_IN_2_O_3_ [M–I]^+^, 301.1547; found, 301.1539.

**1-(3-((1,4-Dioxo-1,4-dihydronaphthalen-2-yl)amino)propyl)-1-methylpiperidin-1-ium iodide** (**2b**). Following general procedure A (1.0 mmol scale), **2b** (327.0 mg, 0.74 mmol, 74% yield) was obtained as a red solid. MP: 225–226 °C. ^1^H NMR (400 MHz, DMSO-*d*_6_) *δ* 8.01–7.99 (m, 2H), 7.88–7.74 (m, 2H), 7.61 (s, 1H), 3.80 (d, *J* = 6.8 Hz, 2H), 3.43–3.36 (m, 2H), 3.32–3.26 (m, 2H), 3.00 (s, 3H), 2.10–2.00 (m, 2H), 1.81–1.74 (m, 4H), 1.58–1.46 (m, 2H). ^13^C NMR (101 MHz, DMSO-*d*_6_) *δ* 180.6, 176.0, 135.5, 133.3, 132.4, 130.5, 127.0, 126.3, 60.6, 60.3, 47.5, 41.6, 23.8, 21.1, 19.7. IR (ATR): ν_max_ 3352, 3002, 2943, 2887, 1671, 1593, 1568, 1510, 1468, 1357, 1275, 1251, 769, 727 cm^−1^. HRMS(+ESI) calcd for C_19_H_25_IN_2_O_2_ [M–I]^+^, 313.1911; found, 313.1906.

**2-((3-Chloro-1,4-dioxo-1,4-dihydronaphthalen-2-yl)amino)-*N*,*N*,*N*-trimethylethan-1-aminium iodide** (**2c**). Following general procedure A (1.0 mmol scale), **2c** (289.8 mg, 0.69 mmol, 69% yield) was red as a yellow solid. MP: 225–226 °C. ^1^H NMR (400 MHz, DMSO-*d*_6_) *δ* 8.00 (dd, *J* = 7.5, 1.4 Hz, 2H), 7.88–7.77 (m, 2H), 7.58 (t, *J* = 6.9 Hz, 1H), 4.15 (q, *J* = 6.4 Hz, 2H), 3.60 (t, *J* = 6.0 Hz, 2H), 3.34 (s, 9H). ^13^C NMR (101 MHz, DMSO-*d*_6_) *δ* 180.6, 176.0, 145.5, 135.4, 133.5, 132.1, 130.7, 127.1, 126.3, 65.5, 65.4, 55.4, 53.2, 38.6, 15.7. IR (ATR): ν_max_ 3225, 3179, 3006, 1681, 1600, 1573, 1504, 1480, 1334, 1292, 1257, 1132, 924, 723 cm^−1^. HRMS(+ESI) calcd for C_15_H_18_ClIN_2_O_2_ [M–I]^+^, 293.1051; found, 293.1048.

**1-(2-((3-Chloro-1,4-dioxo-1,4-dihydronaphthalen-2-yl)amino)ethyl)-1-methylpiperidin-1-ium iodide** (**2d**). Following general procedure A (1.0 mmol scale), **2d** (345.5 mg, 0.75 mmol, 75% yield) was obtained as a red solid. MP: 212–213 °C. ^1^H NMR (400 MHz, DMSO-*d*_6_) *δ* 8.03–7.97 (m, 2H), 7.89–7.76 (m, 2H), 7.54 (t, *J* = 6.9 Hz, 1H), 4.16 (d, *J* = 6.7 Hz, 2H), 3.61 (t, *J* = 6.5 Hz, 2H), 3.50–3.38 (m, 4H), 3.14 (s, 3H), 1.82 (q, *J* = 5.9 Hz, 4H), 1.56 (t, *J* = 6.2 Hz, 2H). ^13^C NMR (101 MHz, DMSO-*d*_6_) *δ* 180.6, 176.0, 145.7, 135.4, 133.4, 132.1, 130.7, 127.1, 126.3, 62.2, 61.0, 48.1, 37.8, 21.0, 19.7. IR (ATR): ν_max_ 3312, 2995, 2945, 1673, 1602, 1572, 1526, 1337, 1252, 1137, 724 cm^−1^. HRMS(+ESI) calcd for C_18_H_22_ClIN_2_O_2_ [M–I]^+^, 333.1364; found, 333.1367.

**3-((3-Chloro-1,4-dioxo-1,4-dihydronaphthalen-2-yl)amino)-*N*,*N*,*N*-trimethylpropan-1-aminium iodide** (**2e**). Following general procedure A (1.0 mmol scale), **2e** (295.1 mg, 0.68 mmol, 68% yield) was obtained as a red solid. MP: 159–160 °C. ^1^H NMR (400 MHz, DMSO-*d*_6_) *δ* 8.00 (s, 1H), 7.98 (s, 1H), 7.87–7.81 (m, 1H), 7.78–7.72 (m, 1H), 7.64 (t, *J* = 6.2 Hz, 1H), 3.93 (dd, *J* = 6.5, 3.4 Hz, 4H), 3.61–3.50 (m, 2H), 3.49–3.38 (m, 4H), 3.30 (s, 2H), 3.16 (s, 3H), 2.08–1.97 (m, 2H). ^13^C NMR (101 MHz, DMSO-*d*_6_) *δ* 182.0, 181.9, 149.0, 135.4, 133.5, 132.8, 130.9, 126.4, 125.8, 100.3, 62.1, 60.3, 59.6, 46.7, 20.4. IR (ATR): ν_max_ 3325, 3008, 1678, 1596, 1566, 1513, 1454, 1336, 1299, 1249, 1135, 718 cm^−1^. HRMS(+ESI) calcd for C_16_H_20_ClIN_2_O_2_ [M–I]^+^, 307.1213; found,307.1211.

**4-(3-((3-Chloro-1,4-dioxo-1,4-dihydronaphthalen-2-yl)amino)propyl)-4-methylmorpholin-4-ium iodide** (**2f**). Following general procedure A (1.0 mmol scale), **2f** (385.5 mg, 0.81 mmol, 81% yield) was obtained as a red solid. MP: 217–218 °C. ^1^H NMR (400 MHz, DMSO-*d*_6_) *δ* 8.00 (d, *J* = 7.6 Hz, 2H), 7.84–7.78 (m, 2H), 7.61 (s, 1H), 4.03–3.87 (m, 4H), 3.82 (q, *J* = 6.5 Hz, 2H), 3.58–3.50 (m, 2H), 3.44 (t, *J* = 4.8 Hz, 4H), 3.15 (s, 3H), 2.14–2.02 (m, 2H). ^13^C NMR (101 MHz, DMSO-*d*_6_) *δ* 180.6, 176.0, 135.4, 133.3, 132.4, 130.5, 127.0, 126.3, 62.0, 60.3, 59.5, 46.4, 41.4, 23.5. IR (ATR): ν_max_ 3321, 2951, 2874, 1678, 1631, 1592, 1565, 1399, 1332, 1268, 1121, 845, 722 cm^−1^. HRMS(+ESI) calcd for C_18_H_22_ClIN_2_O_3_ [M–I]^+^, 349.1313; found, 349.1314.

**4-(2-((3-Bromo-1,4-dioxo-1,4-dihydronaphthalen-2-yl)amino)ethyl)-4-methylmorpholin-4-ium iodide** (**2g**). Following general procedure A (1.0 mmol scale), **2g** (283.9 mg, 0.56 mmol, 56% yield) was obtained as a red solid. MP: 192–193 °C. ^1^H NMR (400 MHz, DMSO-*d*_6_) *δ* 8.02–7.95 (m, 2H), 7.85–7.78 (m, 2H), 7.42 (t, *J* = 6.9 Hz, 1H), 4.19 (q, *J* = 6.6 Hz, 2H), 3.97 (t, *J* = 4.7 Hz, 4H), 3.77 (t, *J* = 6.3 Hz, 2H), 3.55 (q, J = 4.4 Hz, 4H), 3.29 (s, 3H). ^13^C NMR (101 MHz, DMSO-*d*_6_) *δ* 180.3, 175.8, 148.0, 135.2, 133.4, 131.7, 130.9, 127.2, 126.4, 63.2, 60.2, 59.9, 47.5, 38.0. IR (ATR): ν_max_ 3269, 3020, 2871, 1667, 1634, 1565, 1328, 1300, 1243, 965, 882, 794 cm^−1^. HRMS(+ESI) calcd for C_17_H_20_BrIN_2_O_3_ [M–I]^+^, 379.0652; found, 379.0659.

**4-Methyl-4-(2-((3-methyl-1,4-dioxo-1,4-dihydronaphthalen-2-yl)amino)ethyl)morpholin-4-ium iodide** (**2h**) [47]. Following general procedure A (1.0 mmol scale), **2h** (278.5 mg, 0.63 mmol, 63% yield) was obtained as a red solid. MP: 159–160 °C. ^1^H NMR (400 MHz, DMSO-*d*_6_) *δ* 7.95 (d, *J* = 7.8 Hz, 2H), 7.84–7.66 (m, 2H), 6.64 (t, *J* = 6.8 Hz, 1H), 4.07–3.89 (m, 6H), 3.71 (t, *J* = 6.6 Hz, 2H), 3.56–3.46 (m, 4H), 3.25 (s, 3H), 2.05 (s, 3H). ^13^C NMR (101 MHz, DMSO-*d*_6_) *δ* 182.7, 182.1, 146.7, 134.7, 132.8, 132.7, 131.2, 126.2, 125.7, 114.3, 60.3, 59.9, 38.1, 10.9. IR (ATR): ν_max_ 3310, 3267, 3012, 1667, 1593, 1567, 1352, 1224, 1126, 1024, 1003, 919, 718 cm^−1^. HRMS(+ESI) calcd for C_18_H_23_IN_2_O_3_ [M–I]^+^, 315.1703; found, 315.1707.

**1,2,3-Trimethyl-4,9-dioxo-4,9-dihydro-1*H*-naphtho [2,3-*d*]imidazol-3-ium bromide** (**7a**). Following general procedure E (0.3 mmol scale), **7a** (63.6 mg, 0.20 mmol, 66% yield) was obtained as a yellow solid. MP: 230–231 °C. ^1^H NMR (400 MHz, DMSO-*d*_6_) *δ* 8.20 (dd, *J* = 5.7, 3.3 Hz, 2H), 8.01 (dd, *J* = 5.7, 3.3 Hz, 2H), 4.17 (s, 6H), 2.86 (s, 3H). ^13^C NMR (101 MHz, DMSO-*d*_6_) *δ* 175.5, 153.6, 135.8, 132.1, 130.5, 127.3, 34.6, 10.5. IR (ATR): ν_max_ 3429, 3360, 3004, 1674, 1582, 1527, 1402, 1251, 963, 717 cm^−1^. HRMS (+ESI) calcd for C_14_H_13_BrN_2_O_2_ [M–Br]^+^, 241.0972; found, 241.0959.

**1,2,3-Trimethyl-5-nitro-4,9-dioxo-4,9-dihydro-1*H*-naphtho [2,3-*d*]imidazol-3-ium bromide** (**7b**). Following general procedure E (0.3 mmol scale), **7b** (49.5 mg, 0.15 mmol, 45% yield) was obtained as a dark brown solid. MP: 250–251 °C. ^1^H NMR (400 MHz, DMSO-*d*_6_) *δ* 8.42 (dd, *J* = 7.8, 1.3 Hz, 1H), 8.31 (dd, *J* = 8.0, 1.3 Hz, 1H), 8.21 (t, *J* = 7.9 Hz, 1H), 4.17 (s, 3H), 4.11 (s, 3H), 2.87 (s, 3H). ^13^C NMR (101 MHz, DMSO-*d*_6_) *δ* 173.6, 172.5, 154.3, 148.7, 137.3, 133.3, 130.3, 130.2, 129.6, 129.1, 122.3, 34.8, 34.7, 10.6. IR (ATR): ν_max_ 3336, 1685, 1532, 1380, 1251, 994 cm^−1^. HRMS(+ESI) calcd for C_14_H_12_BrN_3_O_4_ [M–Br]^+^, 286.0822; found, 286.0816.

**1,2,3-Trimethyl-6-nitro-4,9-dioxo-4,9-dihydro-1*H*-naphtho [2,3-*d*]imidazol-3-ium bromide** (**7c**). Following general procedure E (0.3 mmol scale), **7c** (45.5 mg, 0.12 mmol, 41% yield) was obtained as an orange solid. MP: 220–221 °C. ^1^H NMR (400 MHz, DMSO-*d*_6_) *δ* 8.78–8.70 (m, 2H), 8.48–8.41 (m, 1H), 4.20 (d, *J* = 3.8 Hz, 6H), 2.90 (s, 3H). ^13^C NMR (101 MHz, DMSO-*d*_6_) *δ* 174.0, 173.7, 154.2, 151.6, 135.9, 133.3, 130.9, 130.8, 129.7, 129.3, 121.7, 34.8, 34.8, 10.7, 10.3. IR (ATR): ν_max_ 3030, 1678, 1607, 1532, 1346, 1258, 988, 716 cm^−1^. HRMS(+ESI) calcd for C_14_H_12_BrN_3_O_4_ [M–Br]^+^, 286.0822; found, 286.0822.

**6-Methoxy-1,2,3-trimethyl-4,9-dioxo-4,9-dihydro-1*H*-naphtho [2,3-*d*]imidazol-3-ium bromide** (**7d**). Following general procedure E (0.3 mmol scale), **7d** (58.9 mg, 0.17 mmol, 56% yield) was obtained as a green solid. MP: 224–225 °C. ^1^H NMR (400 MHz, DMSO-*d*_6_) *δ* 8.15 (d, *J* = 8.7 Hz, 1H), 7.59 (d, *J* = 2.7 Hz, 1H), 7.50 (dd, *J* = 8.7, 2.7 Hz, 1H), 4.16 (d, *J* = 1.9 Hz, 6H), 3.99 (s, 3H), 2.84 (s, 3H). ^13^C NMR (101 MHz, DMSO-*d*_6_) *δ* 175.2, 174.6, 165.0, 153.4, 134.3, 130.6, 130.4, 130.2, 125.0, 120.5, 112.3, 56.9, 34.6, 34.5, 10.4. IR (ATR): ν_max_ 3030, 1671, 1580, 1527, 1264, 1228, 981 cm^−1^. HRMS(+ESI) calcd for C_15_H_15_BrN_2_O_3_ [M–Br]^+^, 271.1077; found, 271.1076.

**1,2,3,7-Tetramethyl-4,9-dioxo-4,9-dihydro-1*H*-naphtho [2,3-*d*]imidazol-3-ium bromide** (**7e**). Following general procedure E (0.3 mmol scale), **7e** (40.1 mg, 0.12 mmol, 40% yield) was obtained as a yellow solid. MP: 224–225 °C. ^1^H NMR (400 MHz, DMSO-*d*_6_) *δ* 8.09 (d, *J* = 7.8 Hz, 1H), 8.00 (d, *J* = 1.8 Hz, 1H), 7.81 (dd, *J* = 7.9, 1.7 Hz, 1H), 4.17 (s, 6H), 2.85 (s, 3H), 2.54 (s, 3H). ^13^C NMR (101 MHz, DMSO-*d*_6_) *δ* 175.6, 175.3, 153.5, 146.9, 136.1, 132.0, 130.5, 130.4, 129.8, 127.7, 127.6, 34.6, 34.6, 21.8, 10.5. IR (ATR): ν_max_ 3441, 3411, 2999, 1674, 1591, 1520, 1398, 1261, 983, 874, 731 cm^−1^. HRMS(+ESI) calcd for C_15_H_15_BrN_2_O_2_ [M–Br]^+^, 255.1128; found, 255.1127.

**1,2,3-Trimethyl-4,9-dioxo-4,9-dihydro-3*H*-imidazo [4,5-*g*]isoquinolin-1-ium bromide** (**7f**). Following general procedure E (0.3 mmol scale), **7f** (64.5 mg, 0.20 mmol, 67% yield) was obtained as a brown solid. MP: 198–199 °C. ^1^H NMR (400 MHz, DMSO-*d*_6_) *δ* 9.37 (d, *J* = 0.8 Hz, 1H), 9.25 (d, *J* = 5.0 Hz, 1H), 8.08 (dd, *J* = 5.0, 0.8 Hz, 1H), 4.18 (d, *J* = 4.3 Hz, 6H), 2.88 (s, 3H). ^13^C NMR (101 MHz, DMSO-*d*_6_) *δ* 175.2, 174.8, 157.3, 154.2, 148.3, 138.0, 130.6, 130.1, 125.1, 119.4, 34.7, 10.6. IR (ATR): ν_max_ 3004, 1675, 1580, 1532, 1413, 1270, 969 cm^−1^. HRMS(+ESI) calcd for C_13_H_12_BrN_3_O_2_ [M–Br]^+^, 242.0924; found, 242.0921.

**1,2,3-Trimethyl-1*H*-naphtho [2,3-*d*]imidazol-3-ium iodide** (**9**). Following general procedure F (0.4 mmol scale), **9** (63.5 mg, 0.19 mmol, 47% yield) was obtained as a brown solid. MP: 212–213 °C. ^1^H NMR (400 MHz, DMSO-*d*_6_) *δ* 8.53 (s, 2H), 8.20–8.14 (m, 2H), 7.71–7.62 (m, 2H), 4.06 (s, 6H), 2.95 (s, 3H). ^13^C NMR (101 MHz, DMSO-*d*_6_) *δ* 157.1, 131.2, 131.1, 128.6, 126.8, 110.2, 32.3, 11.6. IR (ATR): ν_max_ 3457, 2978, 2869, 1545, 1508, 1222, 1039, 956, 875, 749 cm^−1^. HRMS (+ESI) calcd for C_10_H_13_IN_2_ [M–I]^+^, 211.1230; found, 211.1229.

**1,2,3-Trimethyl-1*H*-benzo[*d*]imidazol-3-ium iodide** (**11**) [52]. Following general procedure F (0.4 mmol scale), **11** (74.9 mg, 0.26 mmol, 65% yield) was obtained as a pink solid. MP: 186–187 °C. ^1^H NMR (400 MHz, DMSO-*d*_6_) *δ* 7.99 (d, *J* = 9.4 Hz, 2H), 7.65 (d, *J* = 9.4 Hz, 2H), 4.00 (s, 6H), 2.87 (s, 3H). ^13^C NMR (101 MHz, DMSO-*d*_6_) *δ* 152.8, 131.8, 126.3, 113.2, 32.1, 11.0. IR (ATR): ν_max_ 3353, 3011, 2973, 1539, 1469, 1358, 1127, 1047, 762 cm^−1^. HRMS (+ESI) calcd for C_10_H_13_IN_2_ [M–I]^+^, 161.1073; found, 161.1064.

**1,3-Dimethyl-1*H*-benzo[*d*]imidazol-3-ium iodide** (**13**) [53]. Following general procedure F (0.4 mmol scale), **13** (43.8 mg, 0.16 mmol, 40% yield) was obtained as a beige solid. MP: 127–128 °C. ^1^H NMR (400 MHz, DMSO-*d*_6_) *δ* 9.65 (s, 1H), 8.03 (dd, *J* = 6.2, 3.1 Hz, 2H), 7.72 (dd, *J* = 6.3, 3.1 Hz, 2H), 4.09 (s, 6H). ^13^C NMR (101 MHz, DMSO-*d*_6_) *δ* 143.6, 132.2, 126.9, 113.9, 33.7. IR (ATR): ν_max_ 3512, 3146, 3056, 1597, 1571, 1446, 755 cm^−1^. HRMS (+ESI) calcd for C_9_H_11_IN_2_ [M–I]^+^, 147.0917; found, 147.0907.

**1,2,3-Trimethyl-4,9-dioxo-4,9-dihydro-1*H*-naphtho [2,3-*d*]imidazol-3-ium iodide** (**15**). Following general procedure F (4.0 mmol scale), **15** (55.9 mg, 0.15 mmol, 38% yield) was obtained as a black solid. MP: 205–206 °C. ^1^H NMR (400 MHz, DMSO-*d*_6_) *δ* 8.20 (dd, *J* = 5.7, 3.3 Hz, 2H), 8.01 (dd, *J* = 5.8, 3.3 Hz, 2H), 4.17 (s, 6H), 2.85 (s, 3H). ^13^C NMR (101 MHz, DMSO-*d*_6_) δ 175.5, 153.6, 135.8, 134.4, 132.1, 130.5, 127.3, 126.8, 34.6, 10.4. IR (ATR): ν_max_ 3051, 1676, 1584, 1525, 1251, 1209, 960, 708 cm^−1^. HRMS (+ESI) calcd for C_14_H_13_IN_2_O_2_ [M–I]^+^, 241.0972; found, 241.0973.

**4-(2-(4-((4,9-Dioxo-2-(trifluoromethyl)-4,9-dihydro-1*H*-naphtho [2,3-*d*]imidazol-1-yl)methyl)-1*H*-1,2,3-triazol-1-yl)ethyl)-4-methylmorpholin-4-ium iodide** (**17a**). Following general procedure A (0.2 mmol scale), **17a** (46.9 mg, 0.08 mmol, 39% yield) was obtained as a yellow solid. MP: 196–197 °C. ^1^H NMR (400 MHz, DMSO-*d*_6_) *δ* 8.33 (s, 1H), 8.20–8.09 (m, 2H), 8.00–7.89 (m, 2H), 5.93 (d, *J* = 2.9 Hz, 2H), 4.98 (t, *J* = 6.8 Hz, 2H), 4.04 (t, *J* = 6.9 Hz, 2H), 3.99–3.83 (m, 4H), 3.55–3.41 (m, 4H), 3.18 (s, 3H). ^13^C NMR (101 MHz, DMSO-*d*_6_) *δ* 178.2, 176.6, 141.9, 141.7, 140.4, 135.4, 135.4, 134.9, 134.2, 133.1, 132.8, 127.3, 127.1, 125.0, 120.0, 63.8, 61.9, 60.1, 59.9, 52.2, 46.9, 43.0, 42.9, 26.7. IR (ATR): ν_max_ 2961, 1684, 1592, 1479, 1258, 1213, 1124, 1054, 706 cm^−1^. HRMS(+ESI) calcd for C_22_H_22_F_3_IN_6_O_3_ [M–I]^+^, 475.1700; found, 475.1705.

**1-(2-(4-((4,9-Dioxo-2-(trifluoromethyl)-4,9-dihydro-1*H*-naphtho [2,3-*d*]imidazol-1-yl)methyl)-1*H*-1,2,3-triazol-1-yl)ethyl)-1-methylpiperidin-1-ium iodide** (**17b**). Following general procedure A (0.2 mmol scale), **17b** (42.0 mg, 0.07 mmol, 35% yield) was obtained as a yellow solid. MP: 213–214 °C. ^1^H NMR (400 MHz, DMSO-*d*_6_) *δ* 8.36 (s, 1H), 8.22–8.03 (m, 2H), 8.02–7.82 (m, 2H), 5.93 (s, 2H), 4.97 (t, *J* = 6.8 Hz, 2H), 3.92 (t, *J* = 6.8 Hz, 2H), 3.38–3.28 (m, 4H), 3.06 (s, 3H), 1.78–1.69 (m, 4H), 1.60–1.39 (m, 2H). ^13^C NMR (101 MHz, DMSO-*d*_6_) *δ* 178.1, 176.6, 141.9, 141.7, 140.4, 135.4, 134.9, 134.2, 133.1, 132.8, 127.3, 127.1, 125.0, 120.0, 61.1, 60.9, 47.8, 43.3, 43.0, 31.2, 20.9, 19.7. IR (ATR): ν_max_ 3113, 2950, 1688, 1593, 1468, 1259, 1214, 1193, 1128, 1056, 705 cm^−1^. HRMS(+ESI) calcd for C_23_H_24_F_3_IN_6_O_2_ [M–I]^+^, 473.1907; found, 473.1909.

**1-(3-(4-((4,9-Dioxo-2-(trifluoromethyl)-4,9-dihydro-1*H*-naphtho [2,3-*d*]imidazol-1-yl)methyl)-1*H*-1,2,3-triazol-1-yl)propyl)-1-methylpiperidin-1-ium iodide** (**17c**). Following general procedure A (0.2 mmol scale), **17c** (49.1 mg, 0.08 mmol, 42% yield) was obtained as a yellow solid. MP: 240–241 °C. ^1^H NMR (400 MHz, DMSO-*d*_6_) *δ* 8.26 (s, 1H), 8.20–8.05 (m, 2H), 7.99–7.86 (m, 2H), 5.91 (s, 2H), 4.45 (t, *J* = 6.9 Hz, 2H), 3.36–3.25 (m, 6H), 2.96 (s, 3H), 2.34–2.21 (m, 2H), 1.84–1.60 (m, 4H), 1.59–1.40 (m, 2H). ^13^C NMR (101 MHz, DMSO-*d*_6_) *δ* 178.2, 176.6, 141.6, 141.6, 140.4, 135.3, 134.9, 134.2, 133.1, 132.8, 127.2, 127.1, 124.3, 120.0, 60.6, 48.0, 47.2, 43.0, 22.7, 21.0, 19.7. IR (ATR): ν_max_ 3399, 3069, 2951, 1684, 1591, 1464, 1258, 1214, 1185, 1131, 1052, 1024, 707 cm^−1^. HRMS (+ESI) calcd for C_24_H_26_F_3_IN_6_O_2_ [M–I]^+^, 487.2064; found, 487.2063.

**1-Methyl-1-(3-(2-methyl-4,9-dioxo-4,9-dihydro-1*H*-naphtho [2,3-*d*]imidazol-1-yl)propyl) piperidin-1-ium iodide** (**19**). Following general procedure A (0.2 mmol scale), **19** (47.9 mg, 0.10 mmol, 50% yield) was obtained as an orange solid. MP: 264–265 °C. ^1^H NMR (400 MHz, DMSO-*d*_6_) *δ* 8.10–8.03 (m, 2H), 7.86 (dd, *J* = 5.7, 3.3 Hz, 2H), 4.45 (t, *J* = 7.2 Hz, 2H), 3.52–3.44 (m, 2H), 3.40–3.30 (m, 4H), 3.01 (s, 3H), 2.58 (s, 3H), 2.24 (d, *J* = 3.9 Hz, 2H), 1.79 (d, *J* = 6.0 Hz, 4H), 1.60–1.46 (m, 2H). ^13^C NMR (101 MHz, DMSO-*d*_6_) *δ* 178.8, 176.1, 153.9, 142.9, 134.5, 134.3, 133.2, 132.8, 132.8, 126.8, 126.6, 60.6, 47.5, 42.8, 22.7, 21.1, 19.8, 13.6. IR (ATR): ν_max_ 3490, 2867, 1667, 1585, 1540, 1512, 1468, 1428, 1203, 986, 721 cm^−1.^ HRMS(+ESI) calcd for C_21_H_26_IN_3_O_2_ [M–I]^+^, 352.2020; found, 352.2017.

### 3.2. In Vitro Cell Viability Assay

Reagents and equipment: phosphate-buffered saline (PBS) (Sigma Aldrich, St. Louis, MO, USA), 10× Trypsin-EDTA solution (Sigma Aldrich, 59418C), Dulbecco’s Modified Eagle’s Medium (Sigma Aldrich), foetal bovine serum (FBS), and 100× penicillin-streptomycin solution (Sigma Aldrich P4333). The MTT reagent was prepared by dissolving 5 mg/mL MTT powder in sterile PBS, followed by filtration, whereas the MTT solvent contained 4 mM HCl and 0.1% Nonidet P-40 in isopropanol. Dimethyl sulfoxide (DMSO) was also sourced from Sigma Aldrich. Absorbance measurements were acquired using Kaleido 1.0 software coupled with an EnSight™ HH3400 Multimode Plate Reader (PerkinElmer, Waltham, MA, USA). HEC-1-A endometrial cancer cells were obtained from ATCC, while MAD11 endometrial stromal cells were provided by Dr. Hui Li (University of Virginia). MAD11 cells, derived from non-cancerous donors, were immortalized via human telomerase reverse transcriptase (hTERT).

Method: The methods were adapted from Byrne et al. (2014) [20]. Cells were cultured in DMEM supplemented with 10% FBS and 1% Pen-Strep solution (100 U/mL penicillin and 0.1 mg/mL streptomycin). Upon reaching ~70% confluence, the cells were washed with sterile PBS and detached using a 1× trypsin-EDTA solution. The cells were then resuspended in culture media, counted, and seeded into sterile 96-well tissue culture plates (2500 cells per well for MAD11; 5000 cells per well for HEC-1-A). The cells were incubated at 37 °C with 5% CO_2_ for 24 h prior to drug treatment. Drugs were serially diluted in culture medium to final concentrations of 100, 50, 25, 12.5, 6.3, 3.1, 1.6, and 0.8 µM per well and applied in triplicate. For compounds **7a**–**f** and **15**, as their IC₅₀ values were below 0.8 µM, an additional set of three biological replicates was conducted using a lower concentration range (500, 250, 125, 63, 31, 16, 8, and 4 nM). To minimize variations due to DMSO (vehicle), both drug dilutions and control wells received the same final concentration of DMSO. After 48 h of incubation at 37 °C with 5% CO_2_, cell viability was assessed by adding 20 µL/well of MTT reagent for 3 h. Supernatants were aspirated, and formazan crystals were dissolved in MTT solvent before absorbance was measured at 590/620 nm using an EnSight™ HH3400 Multimode Plate Reader. Cell viability was expressed as a percentage of the vehicle (DMSO-treated) control, with results reported as mean ± SEM from three independent experiments. IC_50_ values were determined using GraphPad Prism 9.

### 3.3. In Silico Molecular Docking Study

Molecular docking studies were performed using Maestro 14.1 (Schrödinger, Inc., New York, NY, USA) in extra-precision mode. The X-ray crystal structure of Keap1 (PDB: 4XMB) was imported into Maestro and prepared using the Protein Preparation Workflow. Ligand structures (**BH10** and **7a**–**f**) were optimized using LigPrep. Receptor grid generation and ligand docking were carried out using default parameters. The resulting protein–ligand complexes were analyzed with Discovery Studio Visualizer (Accelrys Inc., San Diego, CA, USA) and PyMOL (Schrödinger, Inc., New York, NY, USA).

## 4. Conclusions

In this study, a library of salt compounds was synthesized based on **BH10**, and their structure–activity relationship was carefully explored. The 1,4-naphthoquinone series of salt compounds did not exhibit significant improvement and even lost their potency. However, the naphthoimidazole series of salt compounds demonstrated promising potency and remarkable selectivity. In particular, compound **7b** achieved the balance of potency and selectivity, with an IC_50_ of 22.97 nM and an outstanding selectivity of 41.43, and can be regarded as the most promising candidate for further research. In future work, we will expand the cell line panel and proceed with in vivo studies to assess both the anticancer activity and pharmacokinetic properties of the compounds.

## Figures and Tables

**Table 1:** Cytotoxicity data of **BH10** and the synthesized analogues against HEC1A and MAD11 cell lines ^a^.

Compound	Mean IC_50_ (μM)		Selectivity Ratio
	HEC1A ± SD	MAD11 ± SD	
**BH10**	11.84 ± 5.19	28.18 ± 12.20	2.38
**2a**	21.12 ± 2.99	>100	>4.73
**2b**	>100	>100	-
**2c**	97.25 ± 8.20	>100	>1.23
**2d**	58.77 ± 3.01	>100	>1.70
**2e**	>100	>100	-
**2f**	>100	>100	-
**2g**	23.83 ± 4.17	>100	>4.20
**2h**	95.53 ± 14.67	>100	-
**7a**	0.00953 ± 0.0007	0.06569 ± 0.00641	6.89
**7b**	0.02297 ± 0.0033	0.9516 ± 0.2148	41.43
**7c**	0.02882 ± 0.0014	0.06846 ± 0.0061	2.37
**7d**	0.02562 ± 0.0021	0.19390 ± 0.0191	7.57
**7e**	0.01492 ± 0.0012	0.1009 ± 0.0124	6.76
**7f**	0.4724 ± 0.0378	4.8 ± 0.3274	10.16
**9**	23.88 ± 2.97	64.56 ± 13.98	2.70
**11**	>100	>100	-
**13**	>100	>100	-
**15**	0.01347 ± 0.0009	0.1539 ± 0.0109	11.43
**17a**	11.17 ± 2.63	110.9 ± 45.0	9.93
**17b**	6.79 ± 1.0	>100	>14.73
**17c**	5.62 ± 1.43	>100	>17.79
**19**	6.35 ± 1.41	40.79 ± 14.12	6.42

^a^ Values were obtained from MTT cell viability assays (*n* = 3 biological replicates in HEC1A and MAD11 cell lines). SD: standard deviation. Selectivity ratio refers to the IC_50_ value from MAD11 cells divided by the IC_50_ value from HEC1A cells.

## Data Availability

The original contributions presented in this study are included in the article; further inquiries can be directed to the corresponding authors.

## Supplementary Materials

The following supporting information can be downloaded at: https://www.mdpi.com/article/10.3390/molecules30091938/s1, Table S1: Comparison with Selected Reported Glycolysis Inhibitors and salt anticancer agents; ^1^H & ^13^C NMR spectra, MS and IR data for all synthesized final compounds [31,33,39,40,41,42,43,44,45,46,47,48,49].
