# Supplementary material for: Design, Synthesis, and Biological Evaluation of Naphthoquinone Salts as Anticancer Agents
Source: Molecules. 2025 Apr 27;30(9):1938. doi: 10.3390/molecules30091938 (PMC12073298; doi:10.3390/molecules30091938)
Supplement: Supplementary file 1 [file molecules-30-01938-s001.zip › molecules-3581104-supplementary.pdf]

Design, Synthesis and Biological Evaluation of Naphthoquinone salts as Anticancer Agents

Yao Cheng[1], Tsz Tin Yu[1], Ellen M. Olzomer[2], Kyle L. Hoehn[2], Frances L. Byrne[2*] Naresh Kumar[1*] and David StC. Black[1*]

[1] School of Chemistry, University of New South Wales, Sydney, NSW 2052, Australia
[2] School of Biotechnology and Biomolecular Sciences, University of New South Wales, Sydney, NSW 2052, Australia
[*] Corresponding author: d.black@unsw.edu.au (D.B.)

Contents of Supporting Information

Table S1……………………………………………………………………..…………..…..2
NMR Spectra…………………………………………………………….……………..…..3
MS Spectra……………………………………………………………………………….25
IR Spectra……………………………………………………………...…………....36
References……………………………………………………………...…………...47

Table S1. Comparison with Selected Reported Glycolysis Inhibitors and salt anticancer agents

Compound	Class / Scaffold	Reported Target(s)	IC_{50} (μM or nM)	Selectivity Notes	Ref.
**7b**	Naphthoimidazole salt	Keap1 (proposed)	22.97 nM	High (41.43)	—
**BH10**	Quinone derivative	Keap1 (proposed)	11.84 μM	Low (2.38)	1
Lonidamine	Indazole derivative	Hexokinase II	170 μM	Low	2-4
Phloretin	Dihydrochalcone	GLUT1	~2 μM	Limited	5-6
Shikonin	Naphthoquinone	Pyruvate Kinase M2 (PKM2)	19.9 μM	Low	7-8
Lapachol	Naphthoquinone derivative	Indoleamine 2,3-dioxygenase 1 (IDO1)	25 μM	Limited	9-10
cRIPGBM	Quaternary ammonium salt	receptor-interacting protein kinase 2 (RIPK2)	27 nM	Variable, brain-specific	11-12
YM155	Quaternary ammonium salt	Survivin suppression (indirect)	13.7 nM	18	13

NMR Spectra

^{1}H NMR spectrum of compound **2a** (DMSO-d_6, 298 K, 400 MHz)

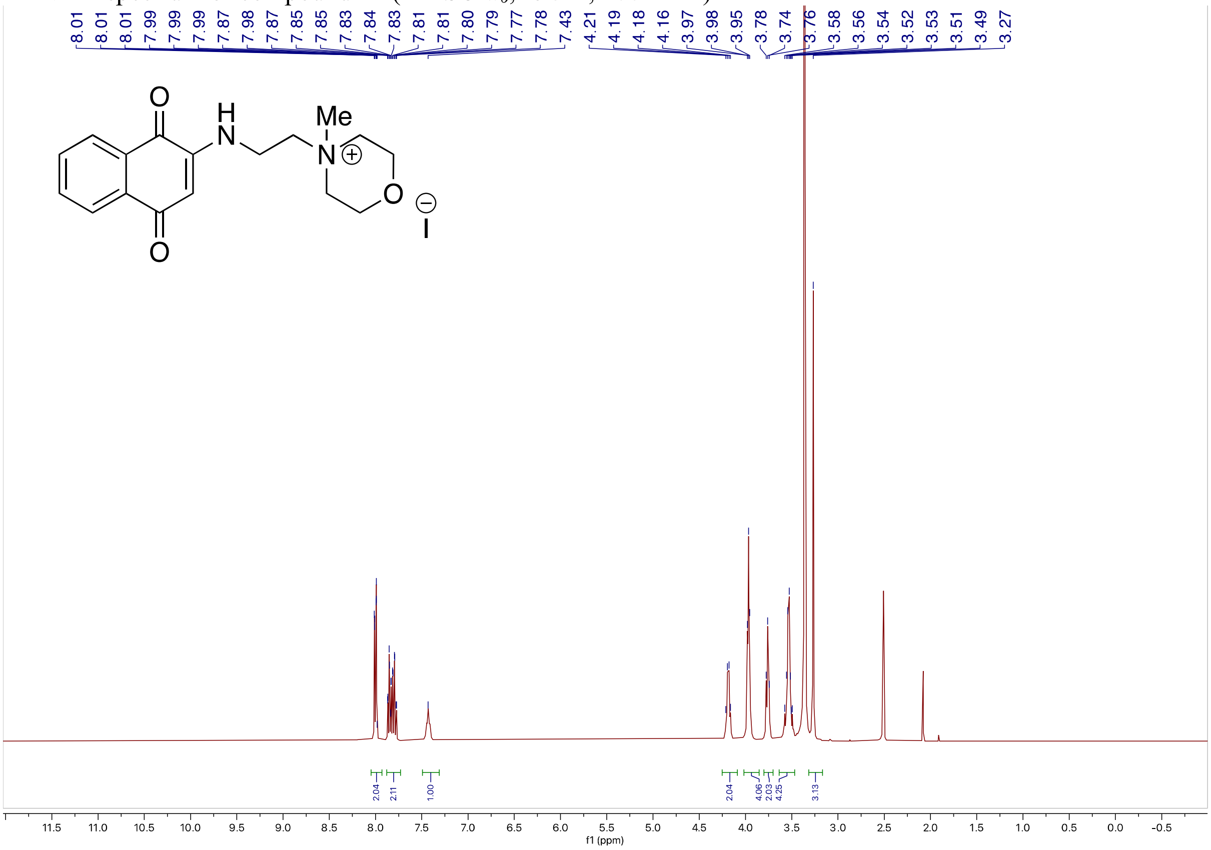

^{13}C NMR spectrum of compound **2a** (DMSO-d_6, 298 K, 101 MHz)

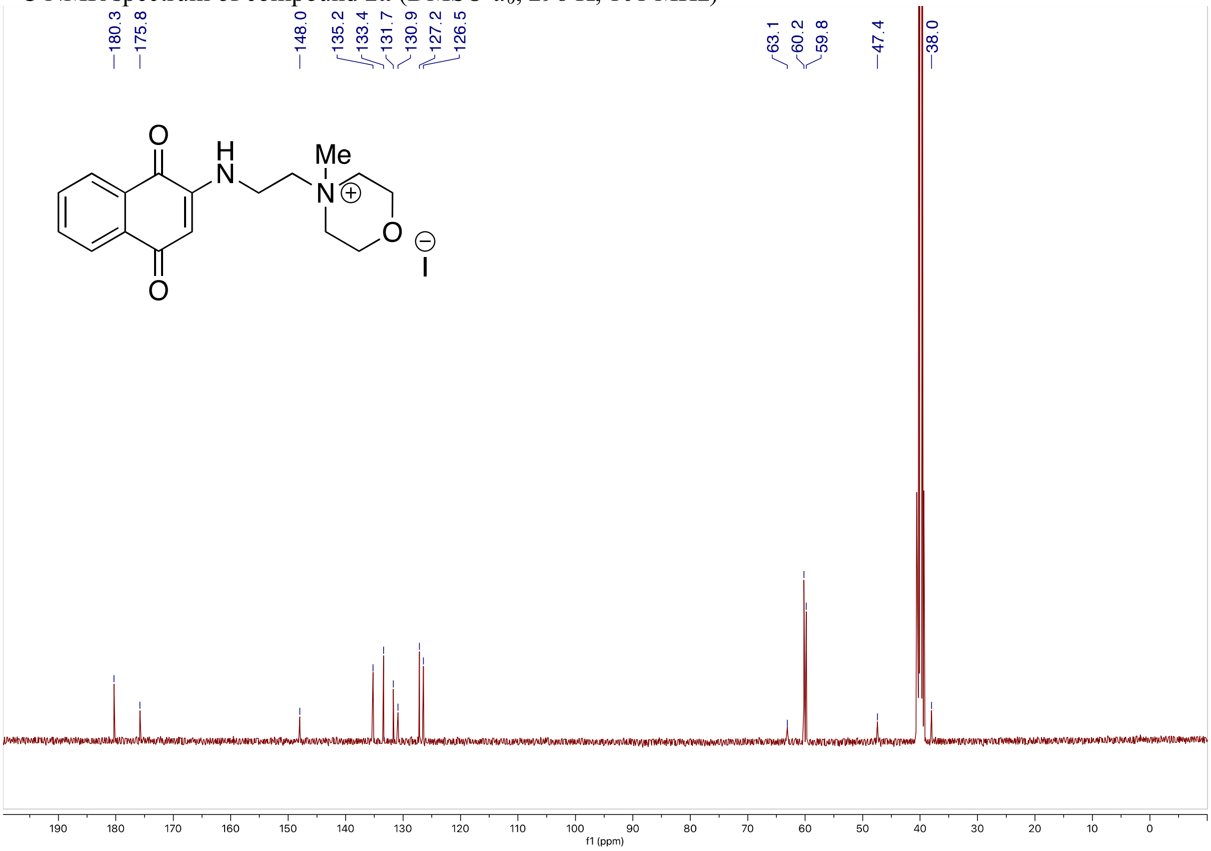

^{1}H NMR spectrum of compound **2b** (DMSO-d_6, 298 K, 400 MHz)

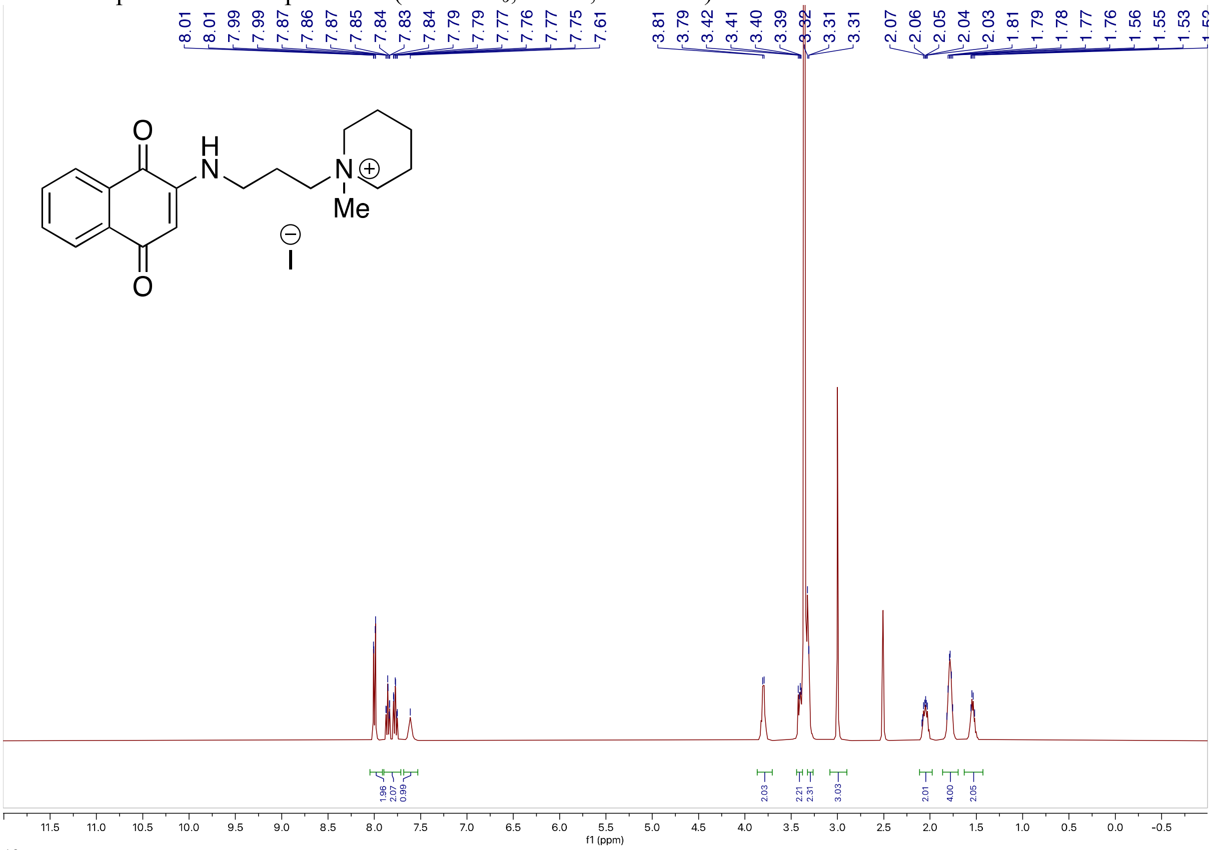

^{13}C NMR spectrum of compound **2b** (DMSO-d_6, 298 K, 101 MHz)

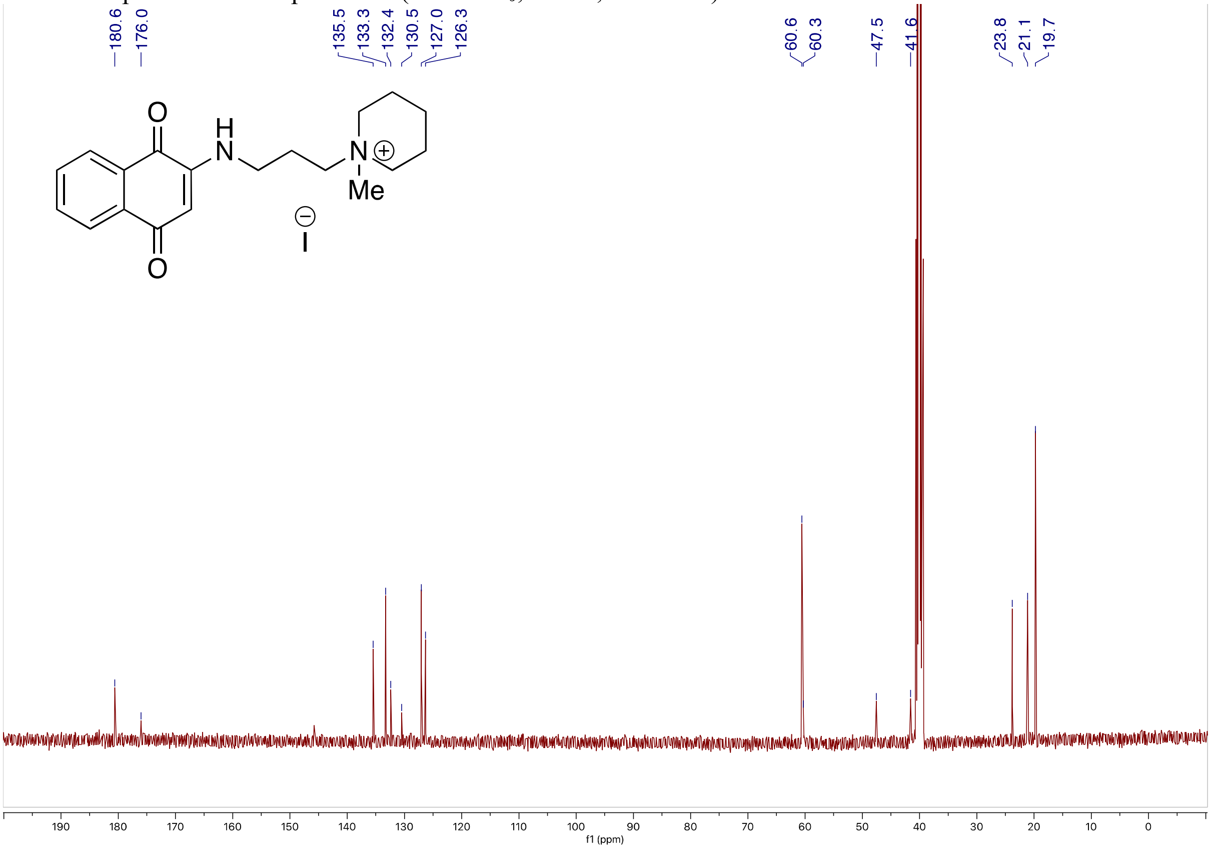

^{1}H NMR spectrum of compound **2c** (DMSO-d_6, 298 K, 400 MHz)

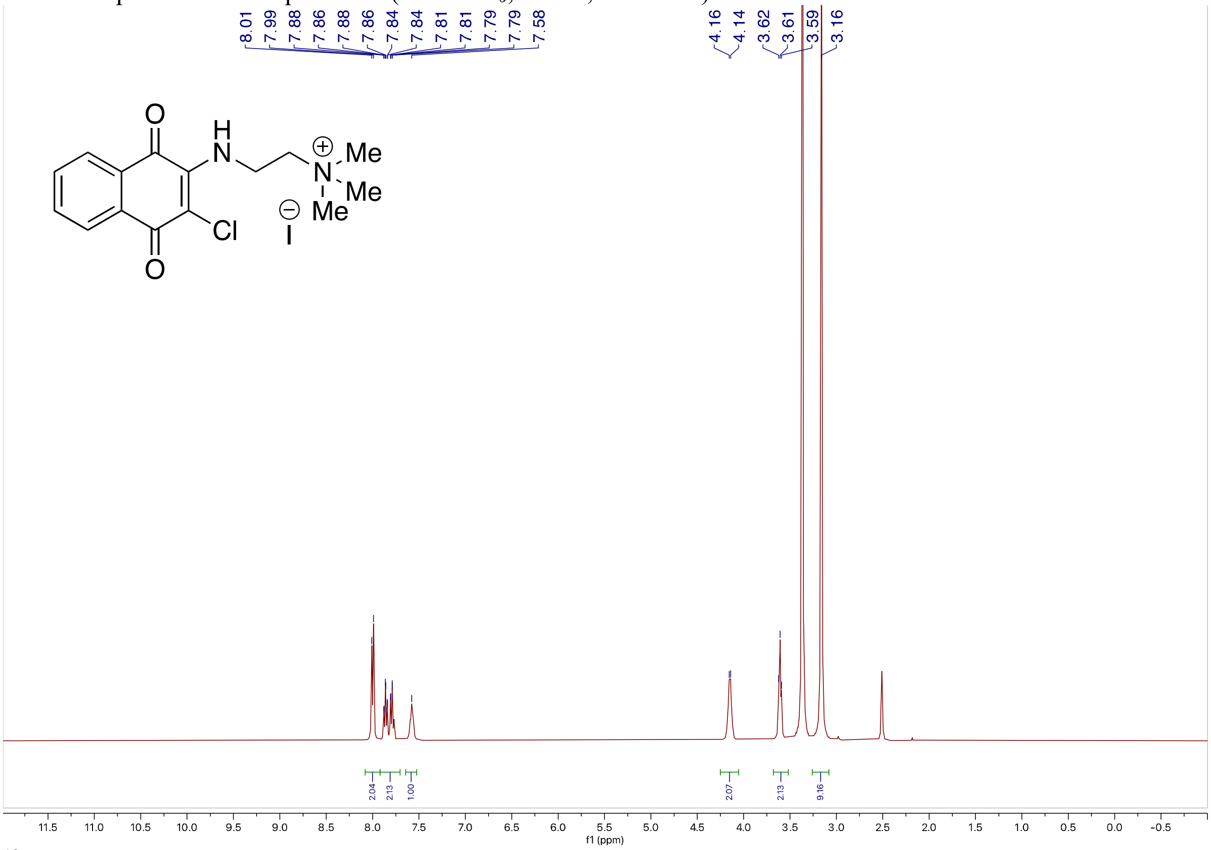

^{13}C NMR spectrum of compound **2c** (DMSO-d_6, 298 K, 101 MHz)

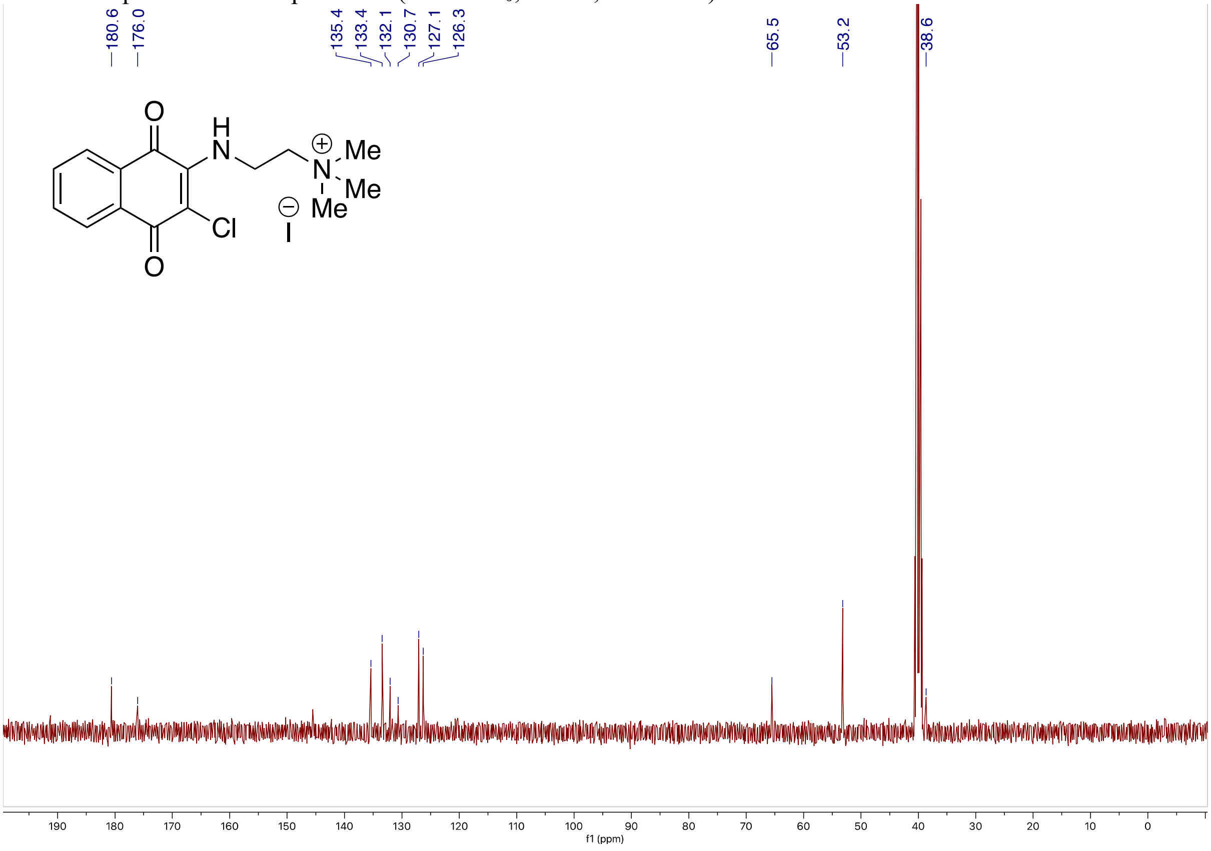

^{1}H NMR spectrum of compound **2d** (DMSO-d_6, 298 K, 400 MHz)

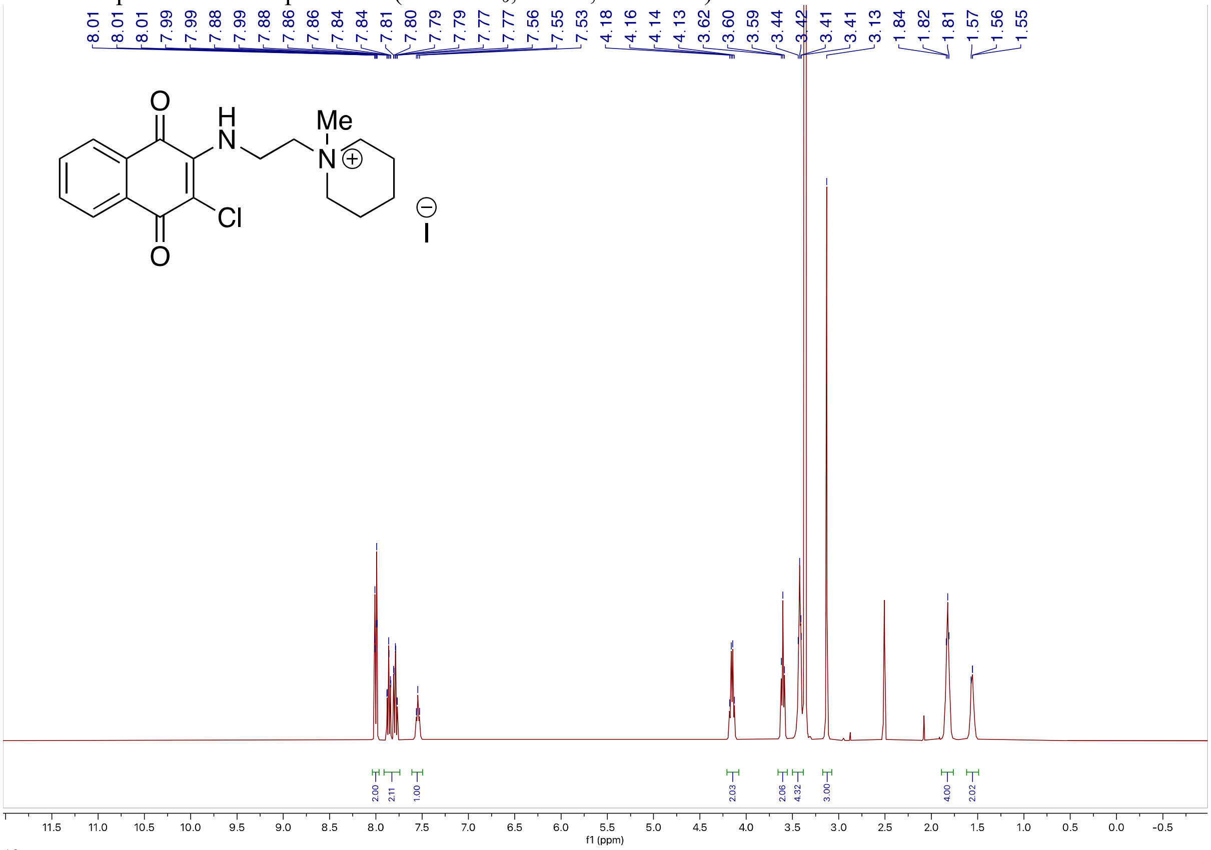

^{13}C NMR spectrum of compound **2d** (DMSO-d_6, 298 K, 101 MHz)

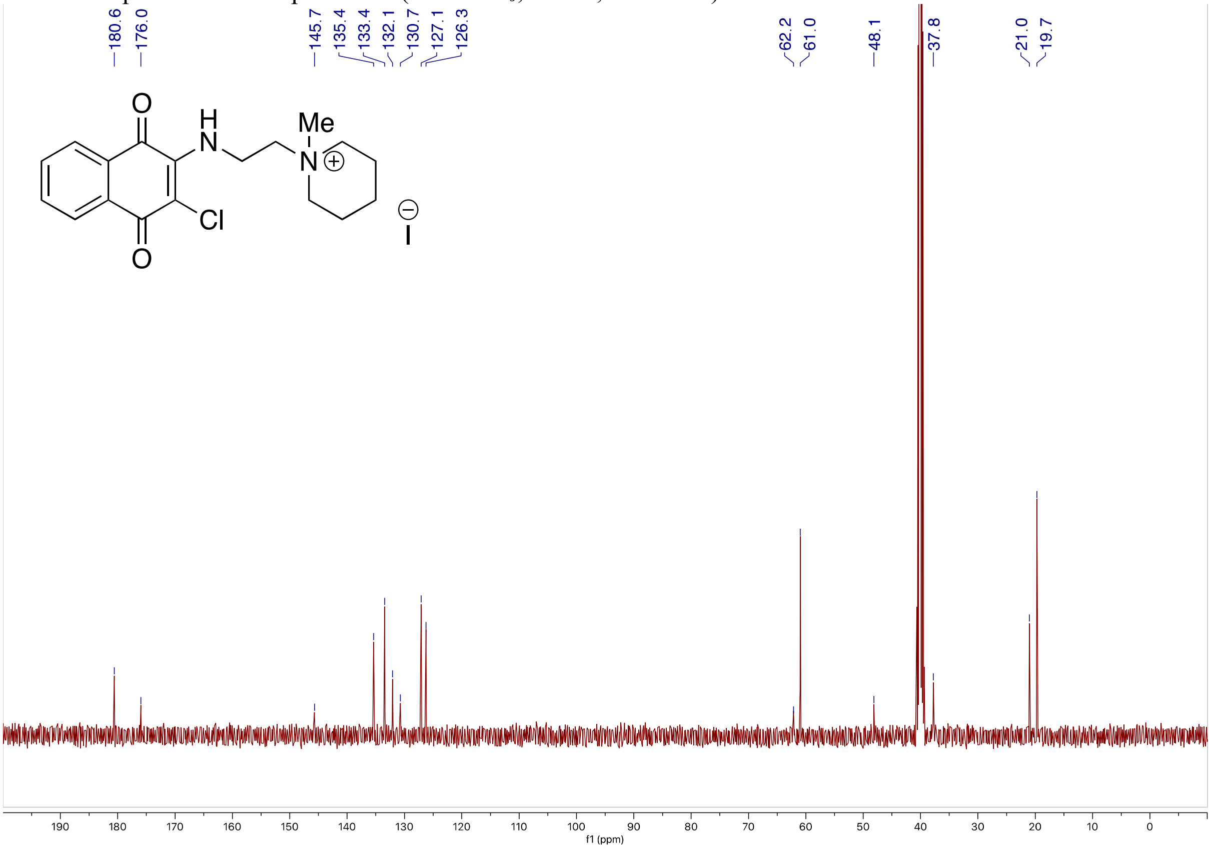

^{1}H NMR spectrum of compound **2e** (DMSO-d_6, 298 K, 400 MHz)

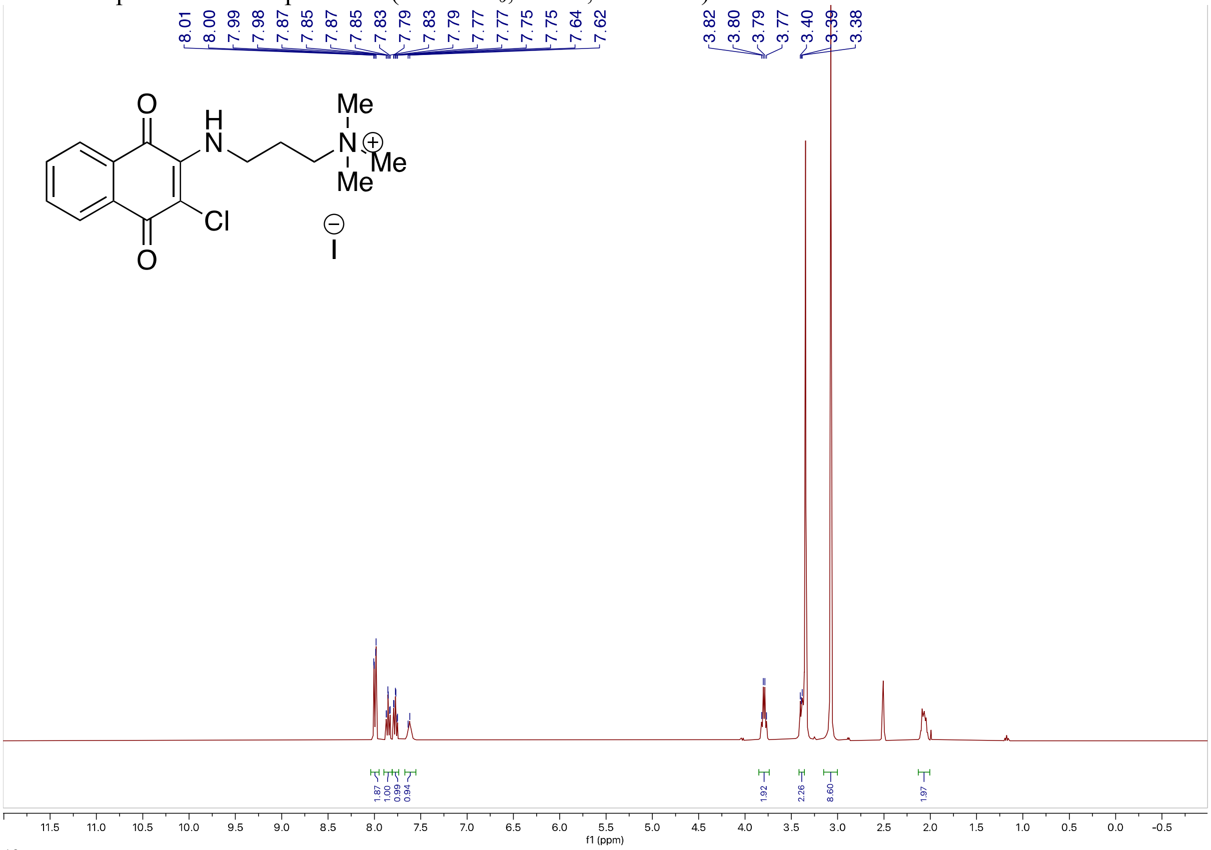

^{13}C NMR spectrum of compound **2e** (DMSO-d_6, 298 K, 101 MHz)

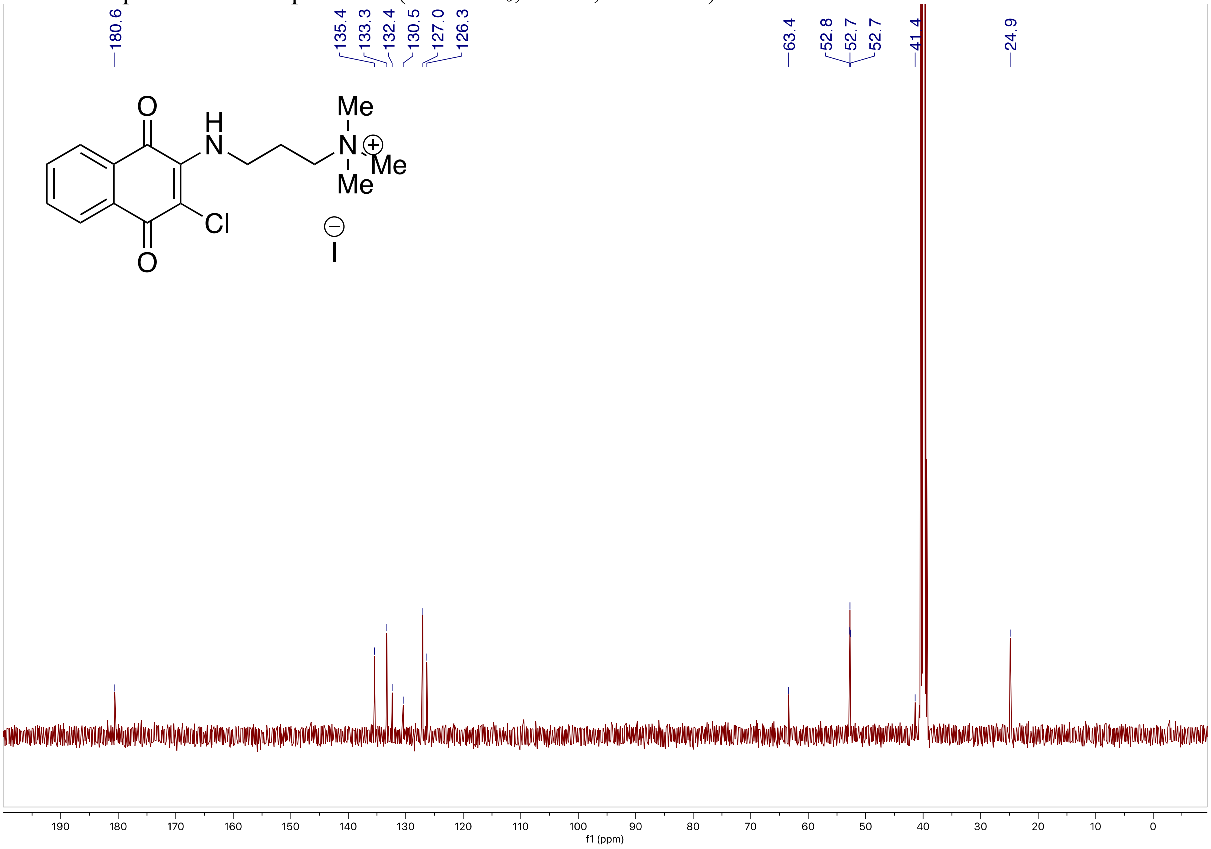

^{1}H NMR spectrum of compound **2f** (DMSO-d_6, 298 K, 400 MHz)

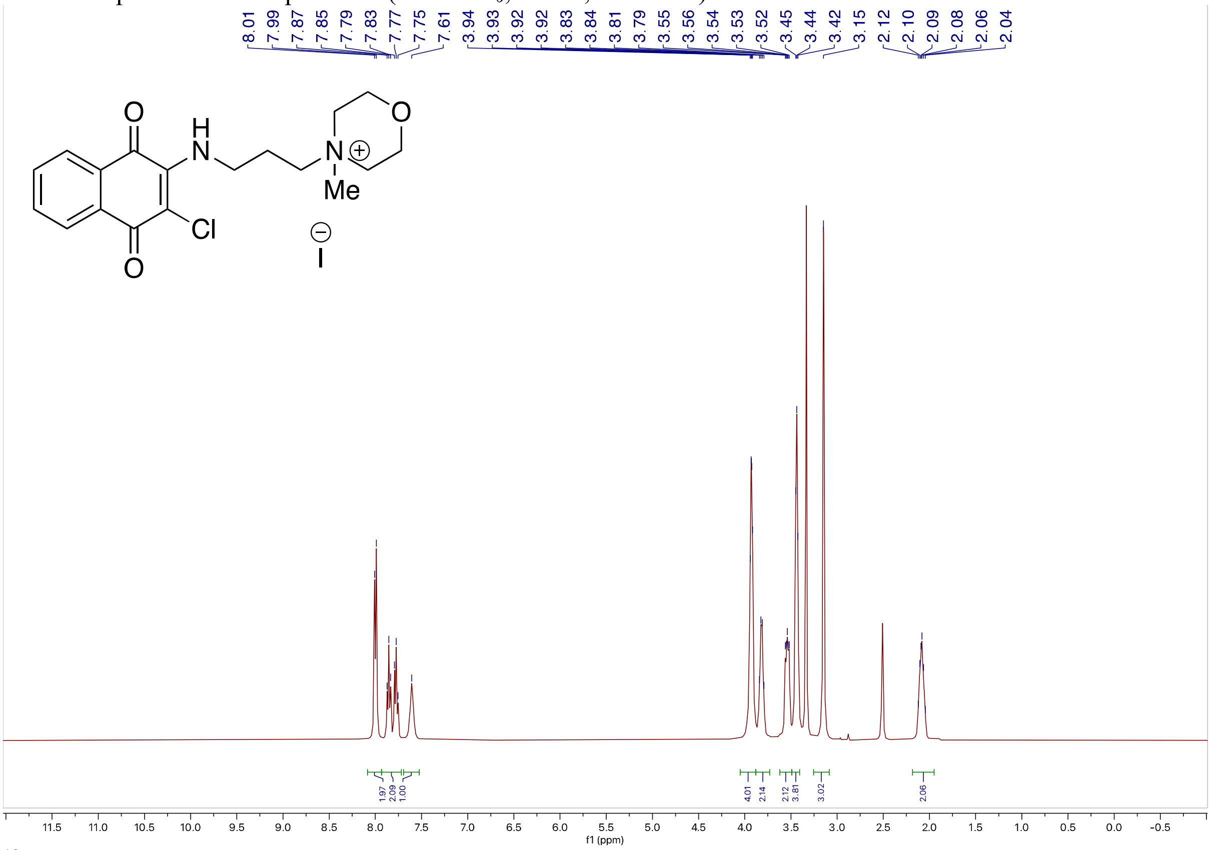

^{13}C NMR spectrum of compound **2f** (DMSO-d_6, 298 K, 101 MHz)

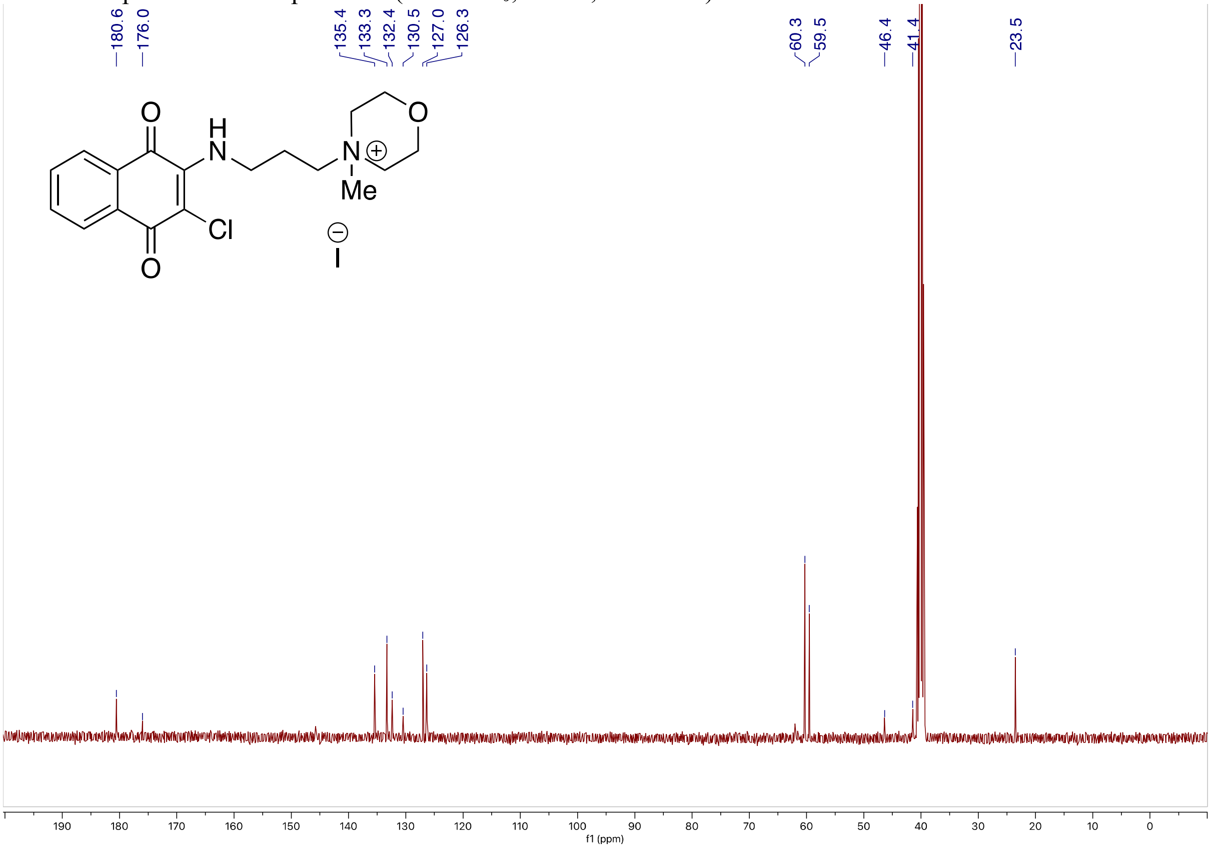

^{1}H NMR spectrum of compound **2g** (DMSO-d_6, 298 K, 400 MHz)

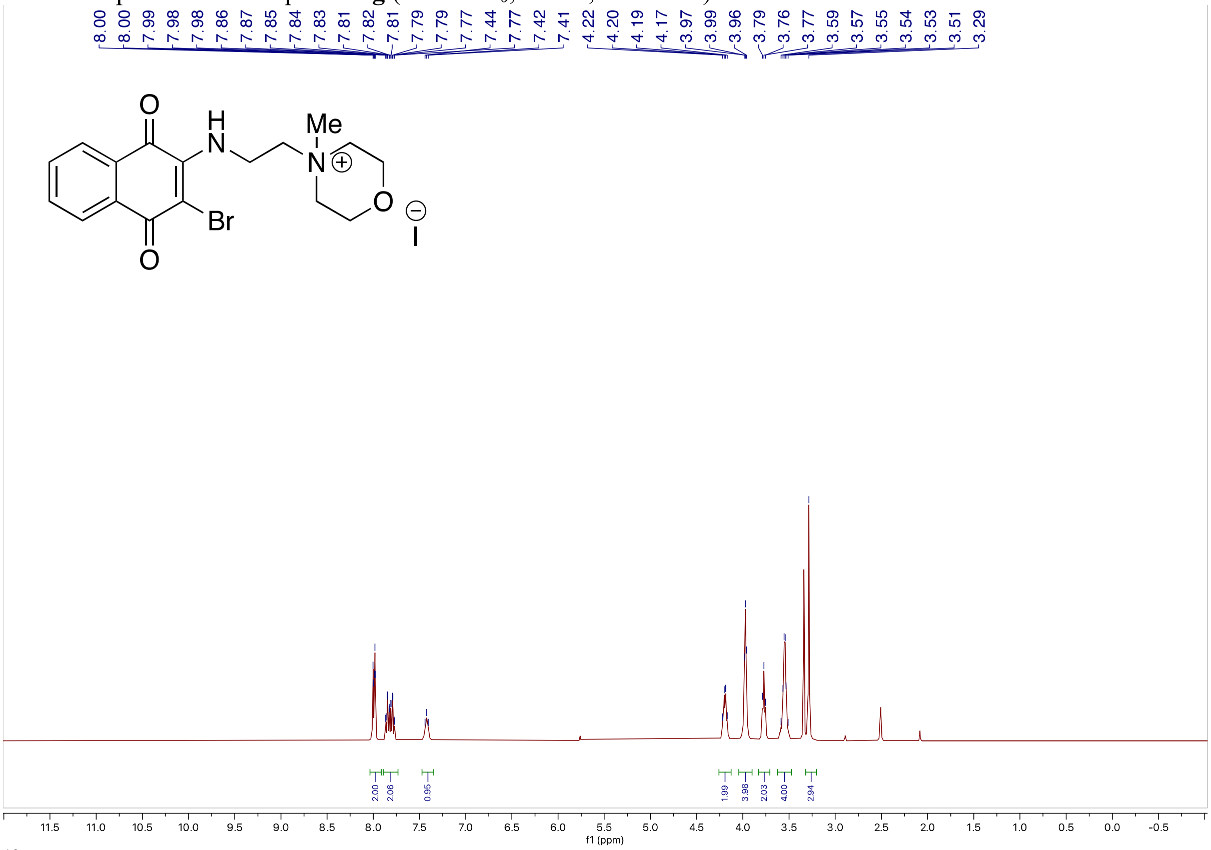

^{13}C NMR spectrum of compound **2g** (DMSO-d_6, 298 K, 101 MHz)

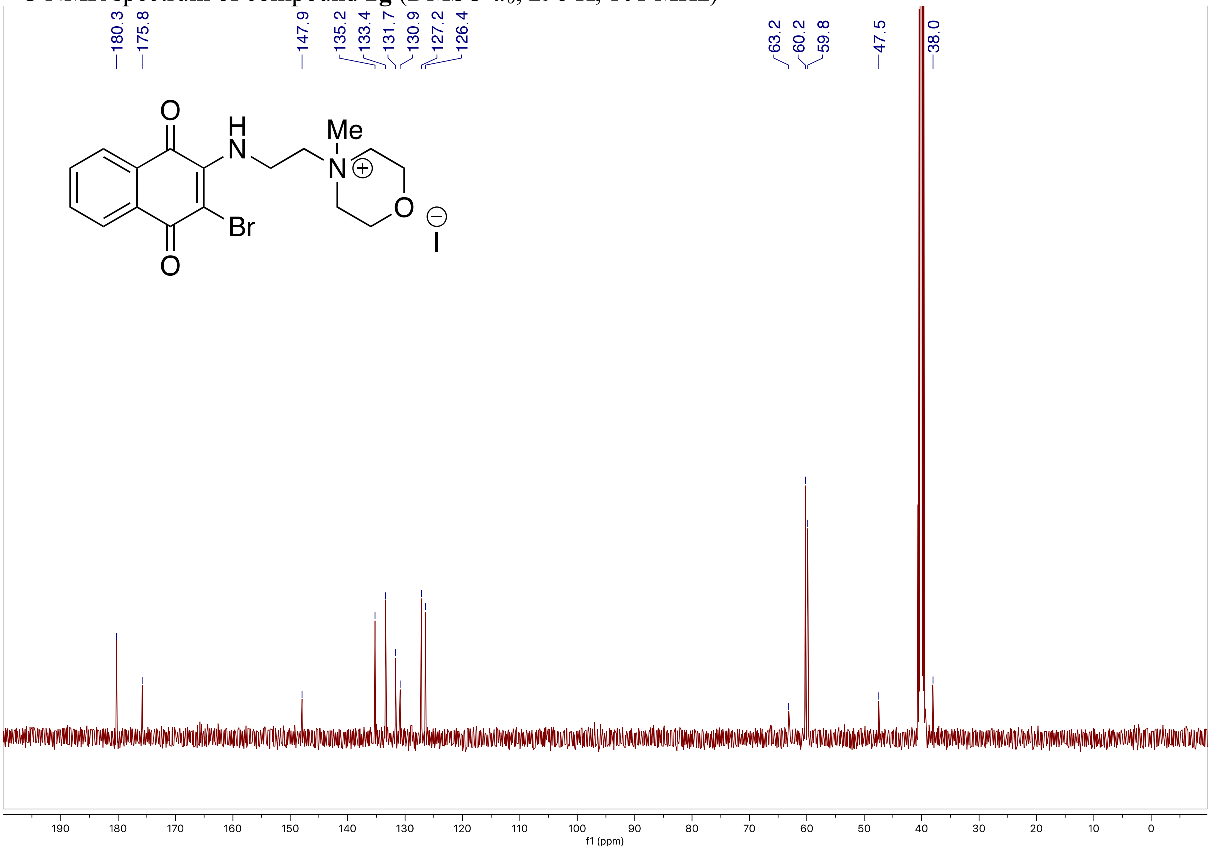

^{1}H NMR spectrum of compound **2h** (DMSO-d_6, 298 K, 400 MHz)

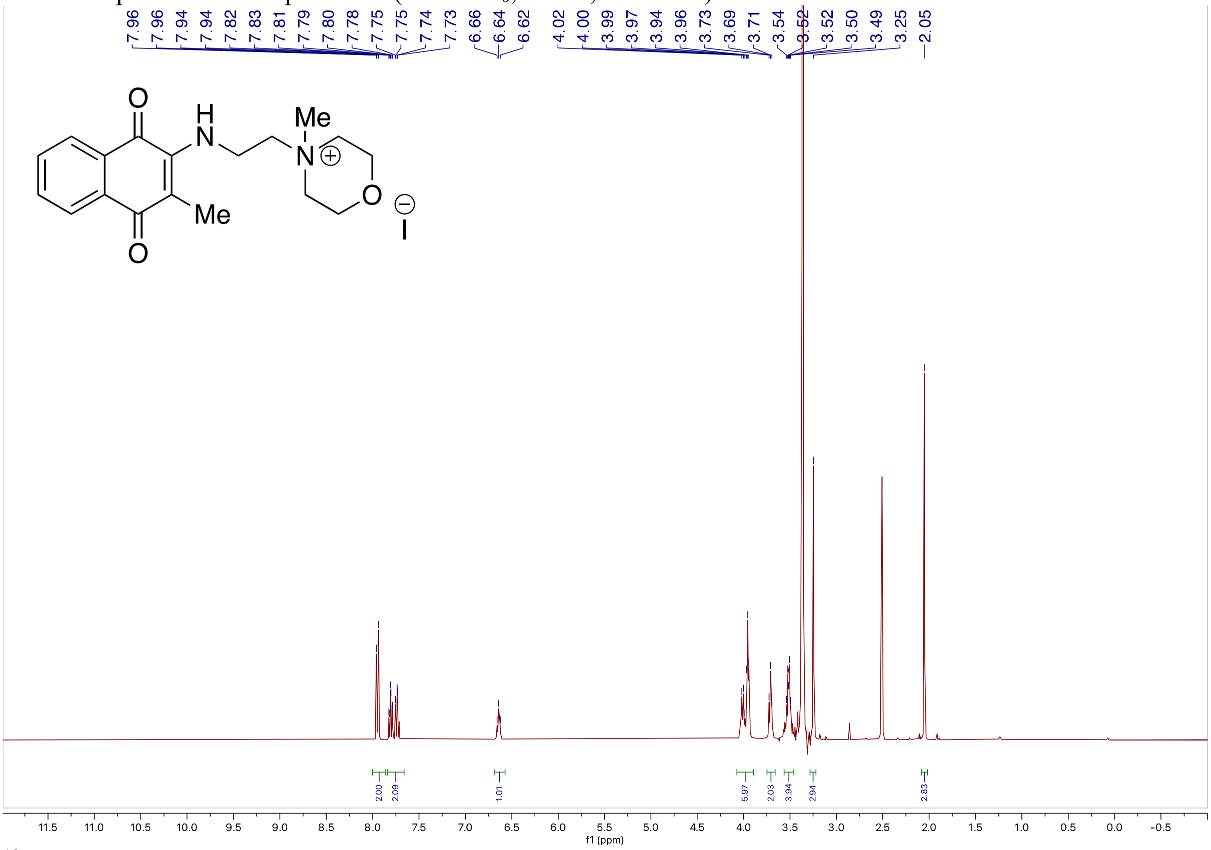

^{13}C NMR spectrum of compound **2h** (DMSO-d_6, 298 K, 101 MHz)

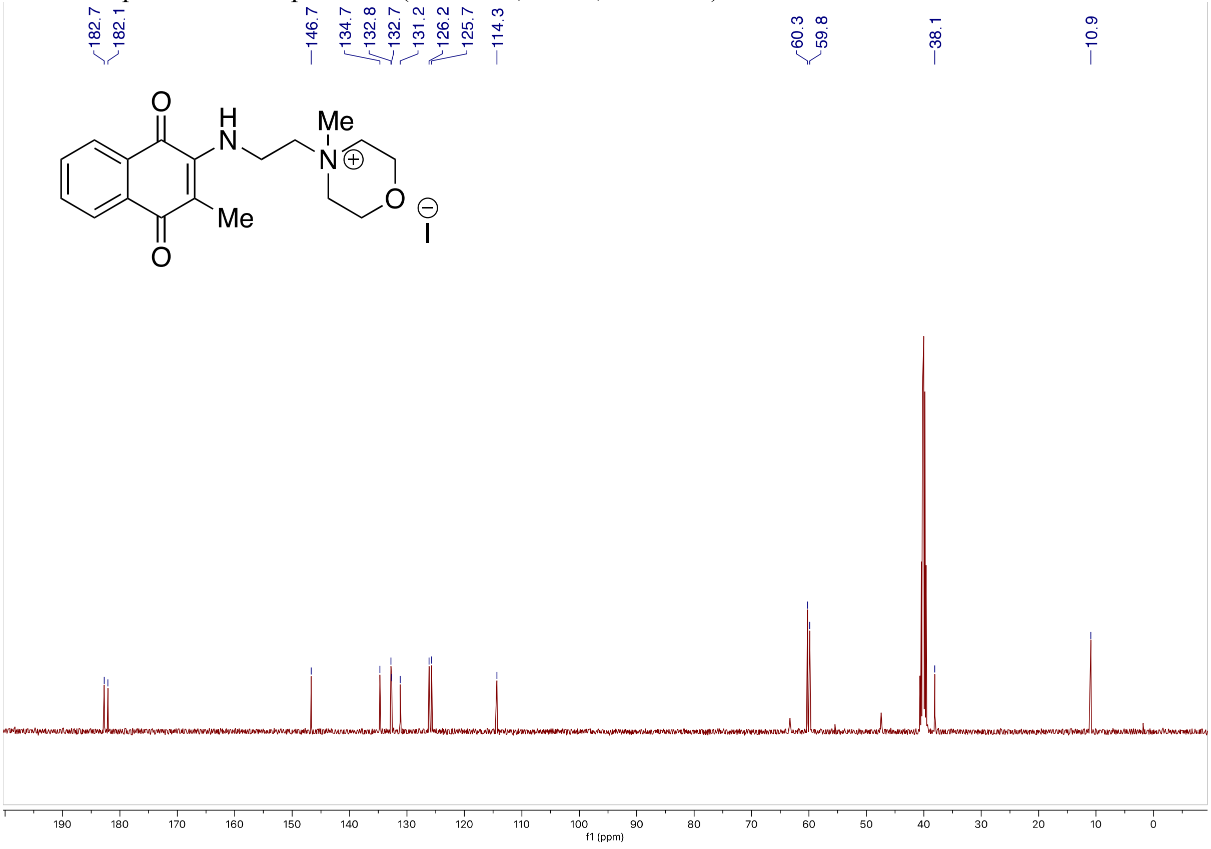

^{1}H NMR spectrum of compound **7a** (DMSO-d_6, 298 K, 400 MHz)

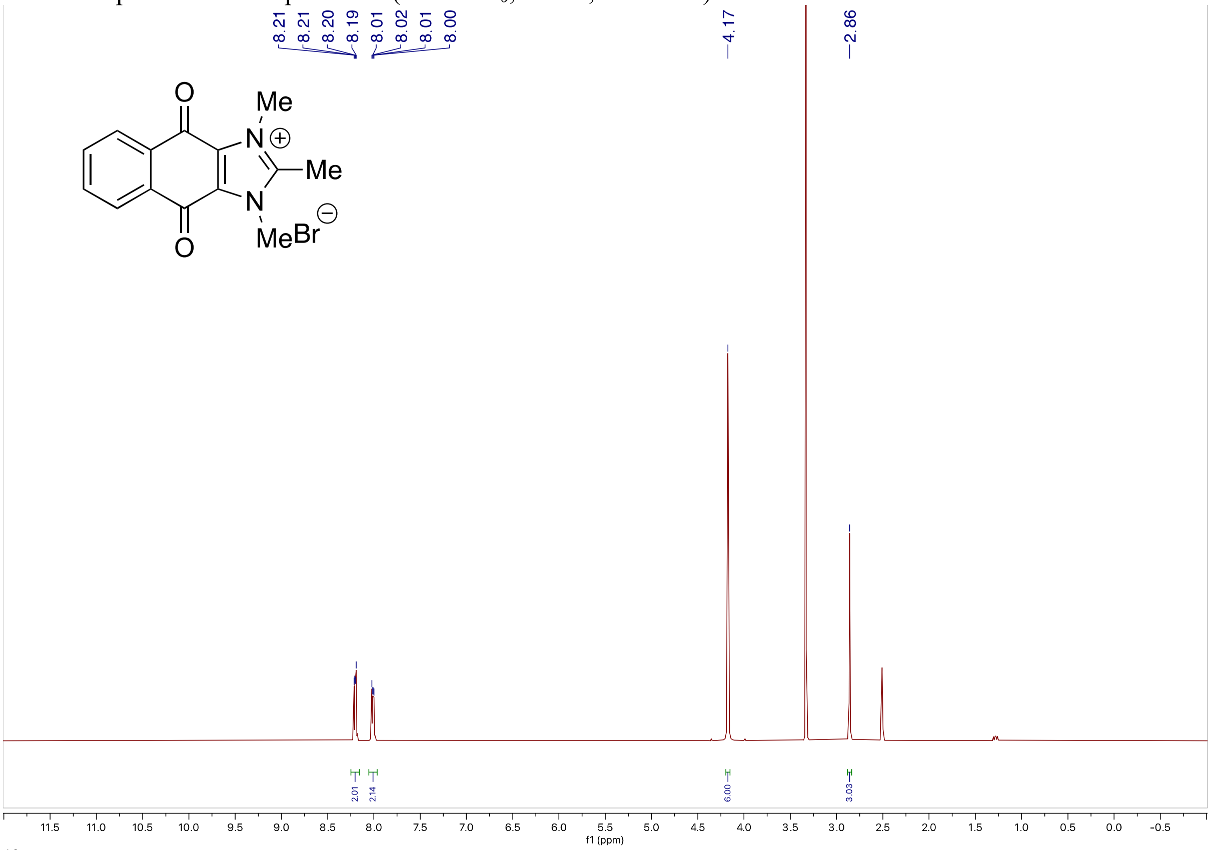

^{13}C NMR spectrum of compound **7a** (DMSO-d_6, 298 K, 101 MHz)

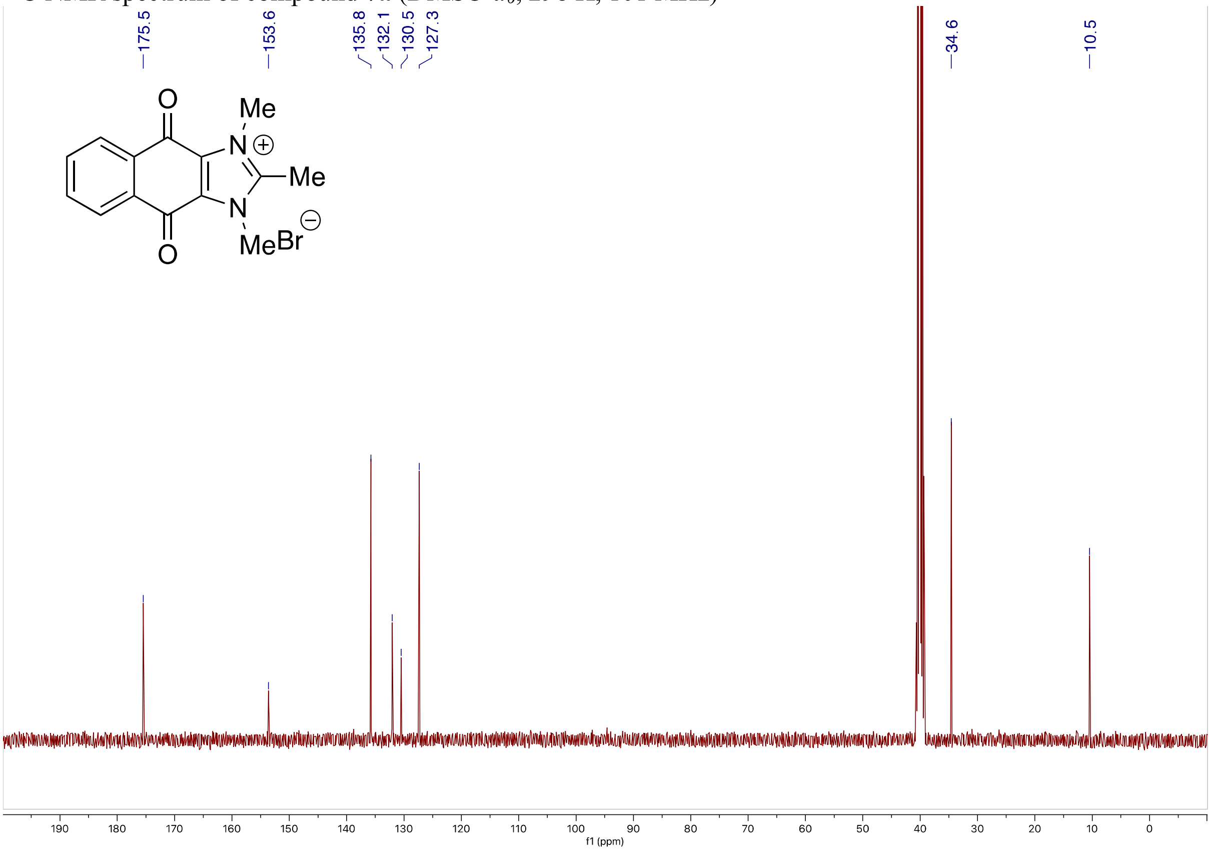

^{1}H NMR spectrum of compound **7b** (DMSO-d_6, 298 K, 400 MHz)

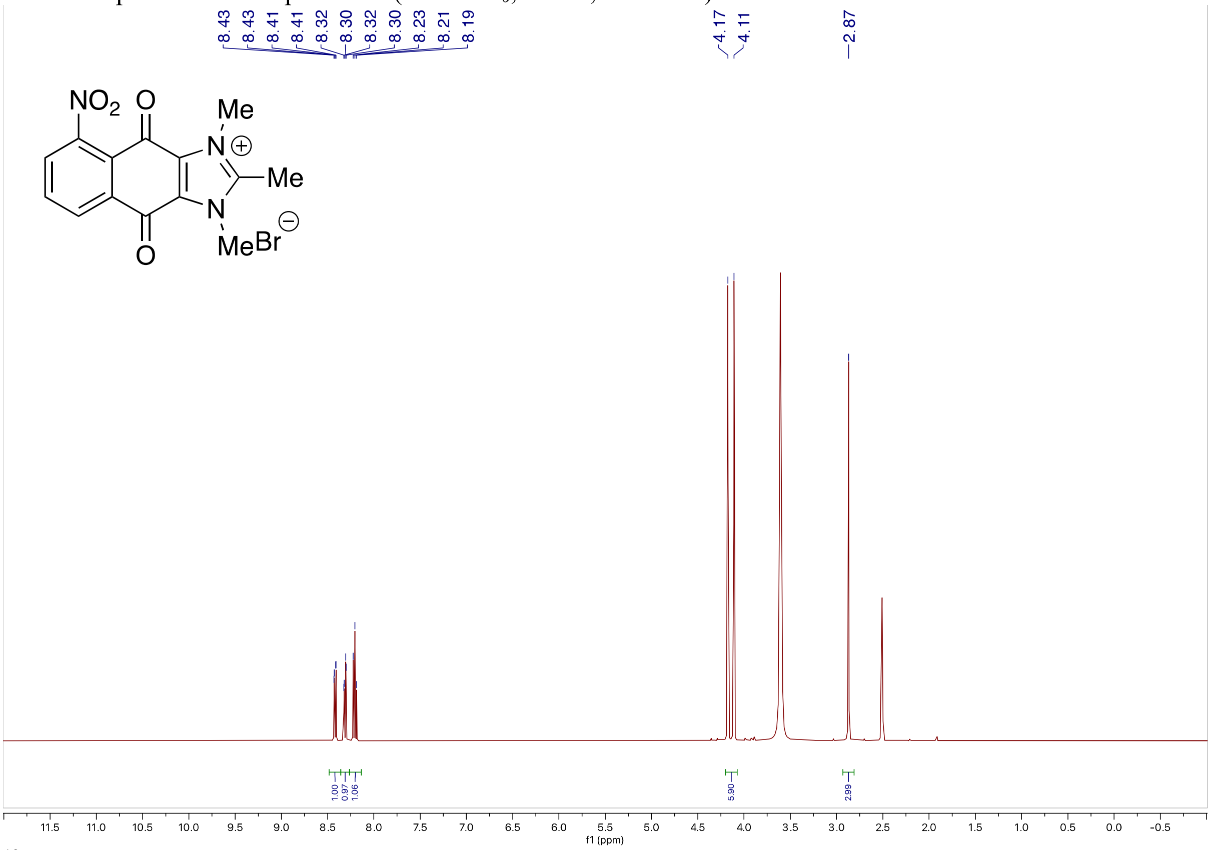

^{13}C NMR spectrum of compound **7b** (DMSO-d_6, 298 K, 101 MHz)

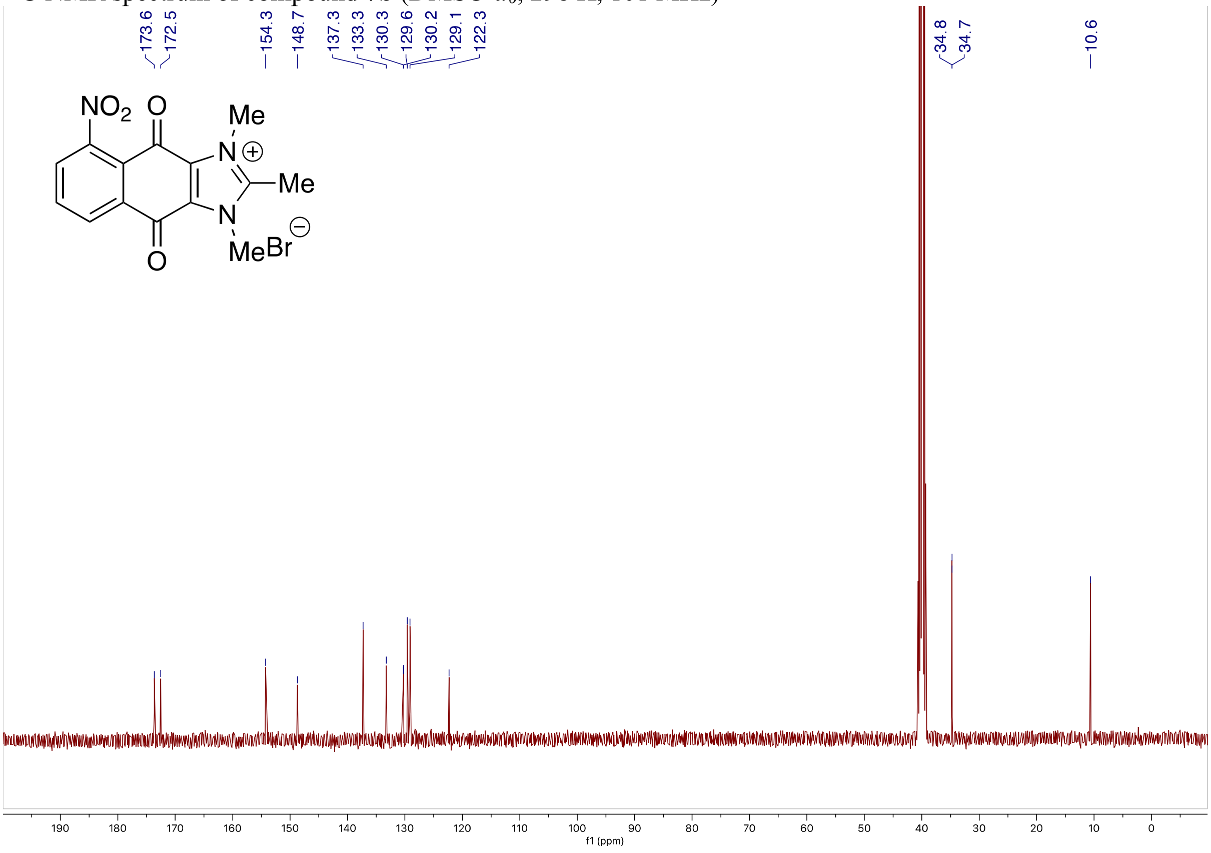

^{1}H NMR spectrum of compound **7c** (DMSO-d_6, 298 K, 400 MHz)

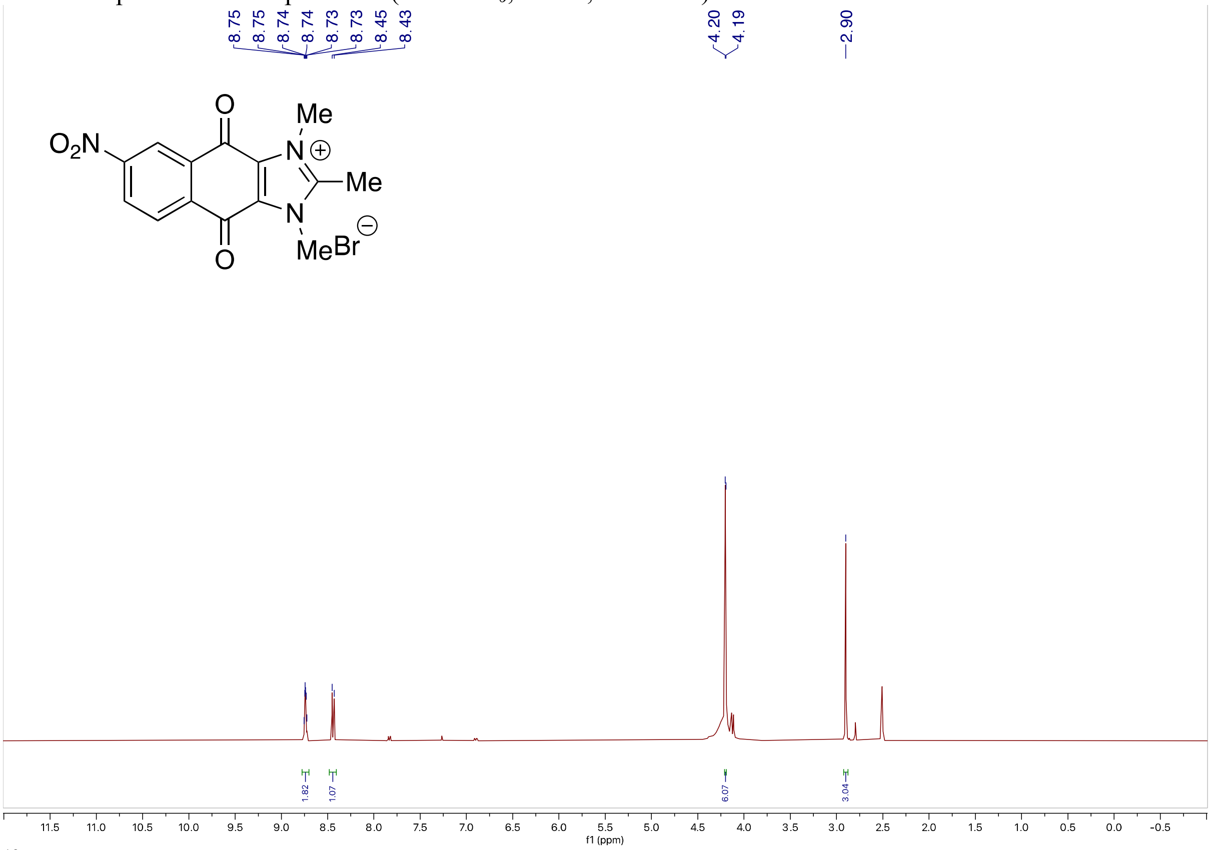

^{13}C NMR spectrum of compound **7c** (DMSO-d_6, 298 K, 101 MHz)

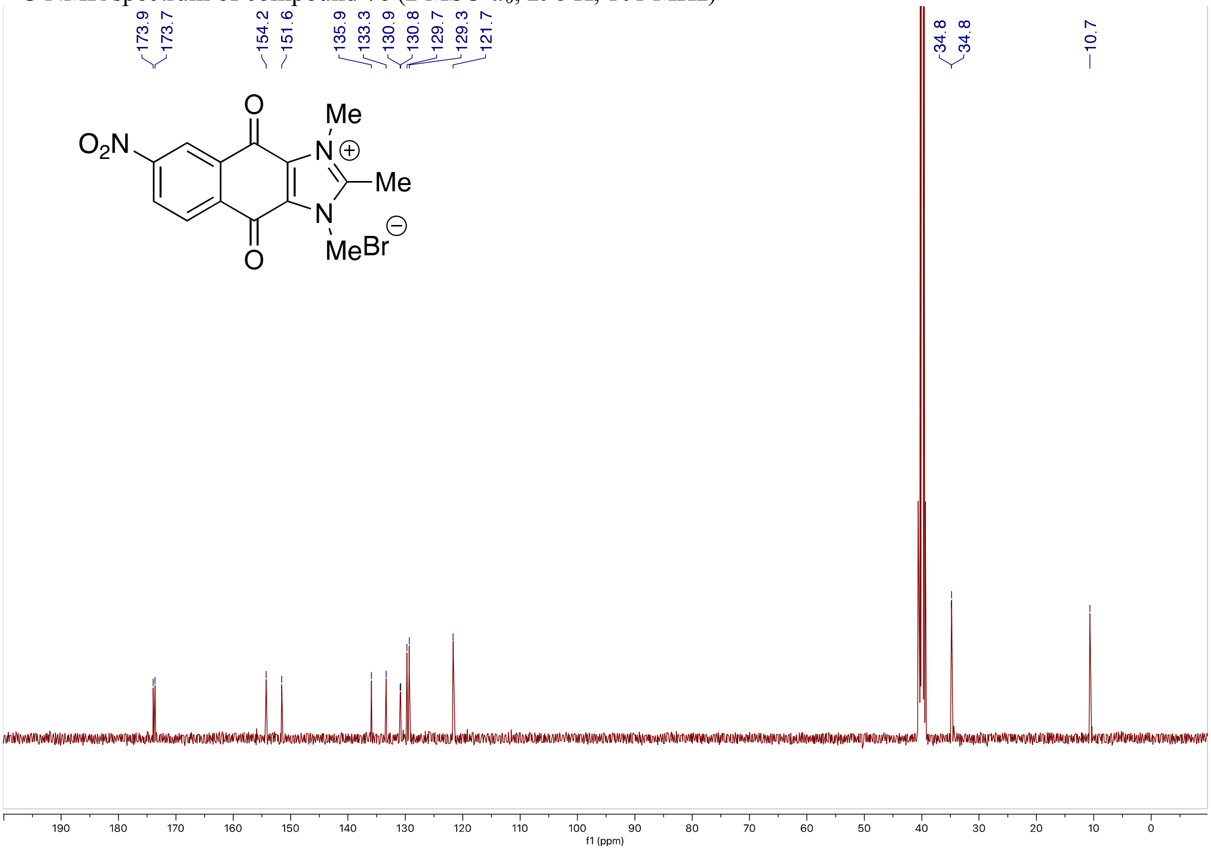

^{1}H NMR spectrum of compound **7d** (DMSO-d_6, 298 K, 400 MHz)

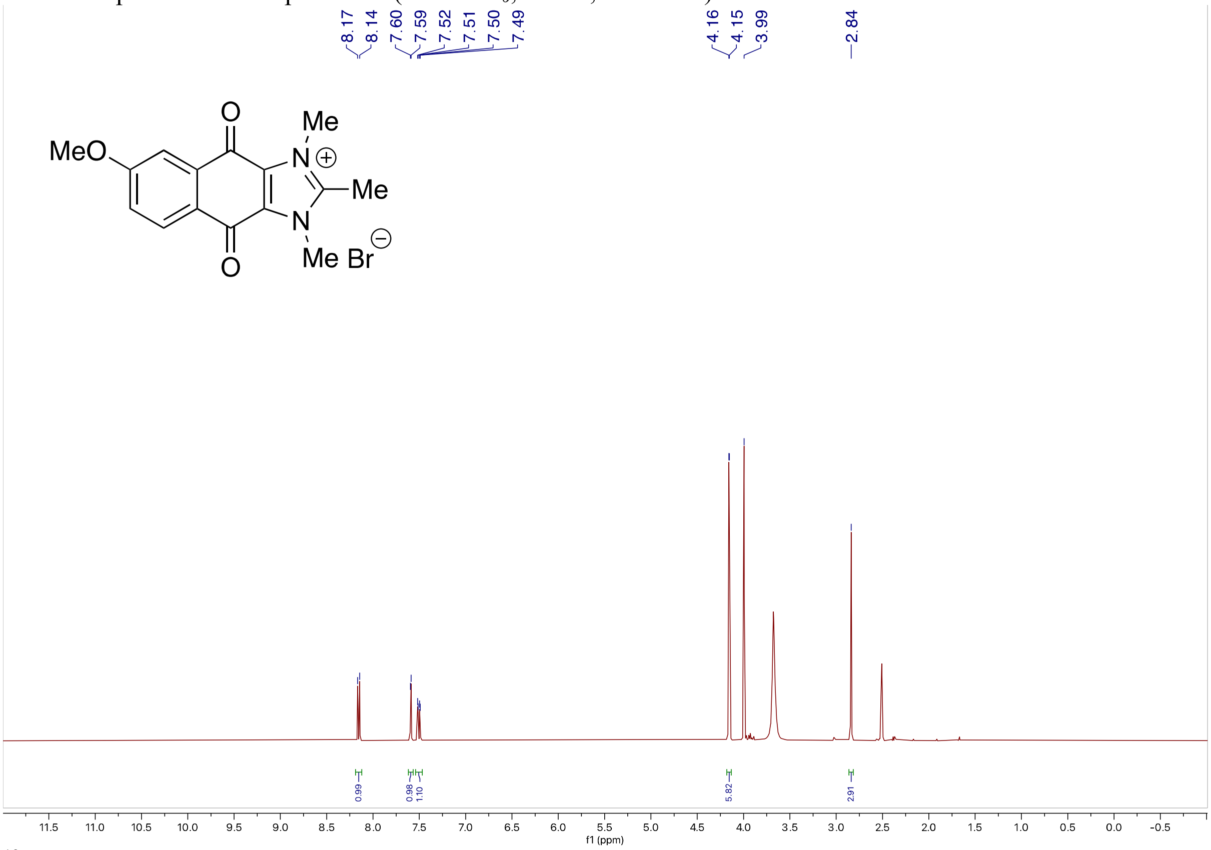

^{13}C NMR spectrum of compound **7d** (DMSO-d_6, 298 K, 101 MHz)

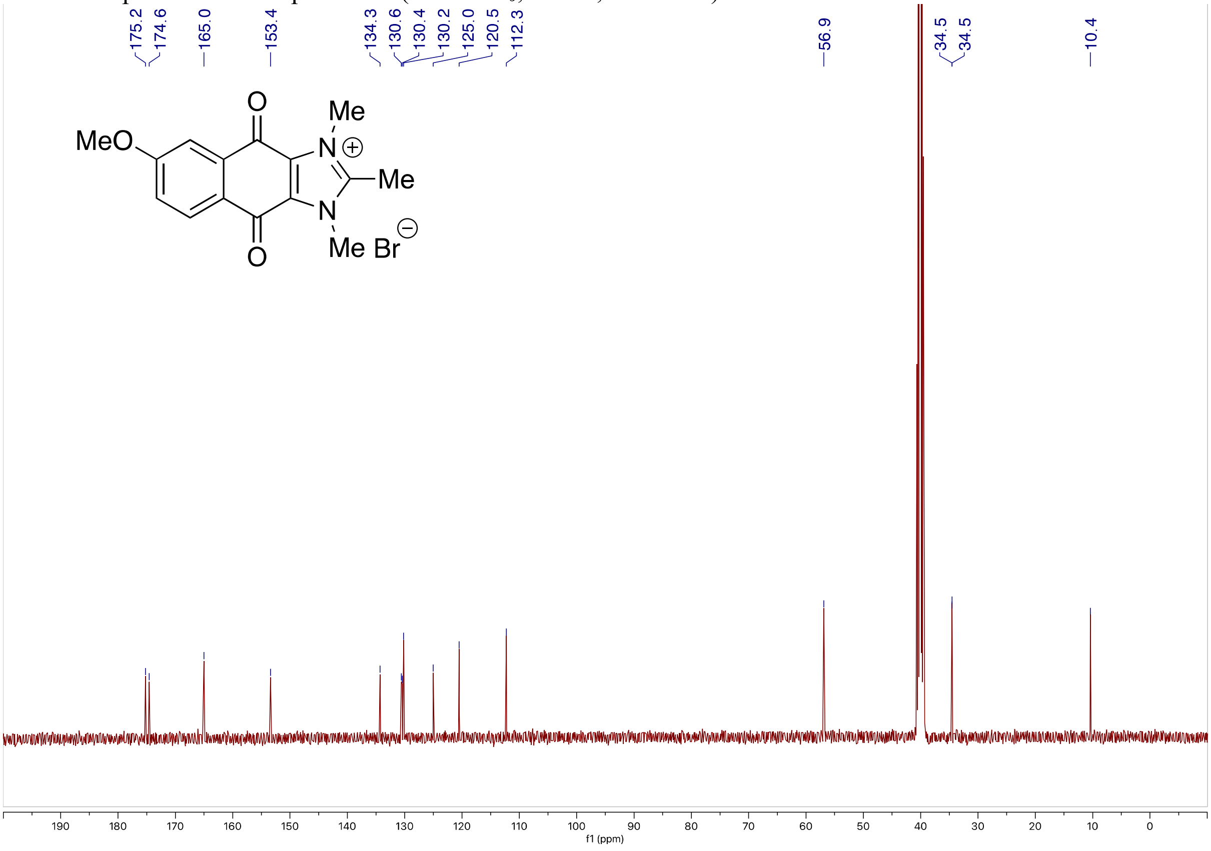

^{1}H NMR spectrum of compound **7e** (DMSO-d_6, 298 K, 400 MHz)

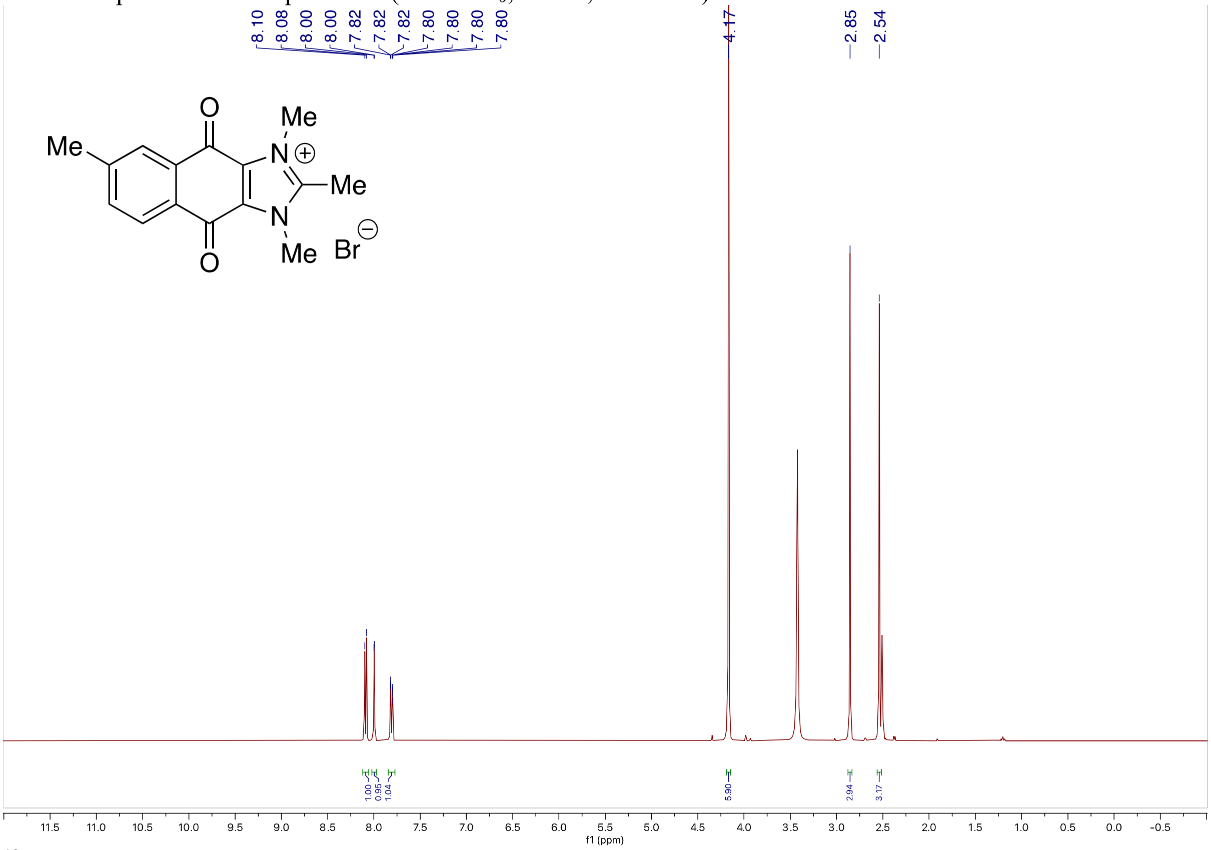

^{13}C NMR spectrum of compound **7e** (DMSO-d_6, 298 K, 101 MHz)

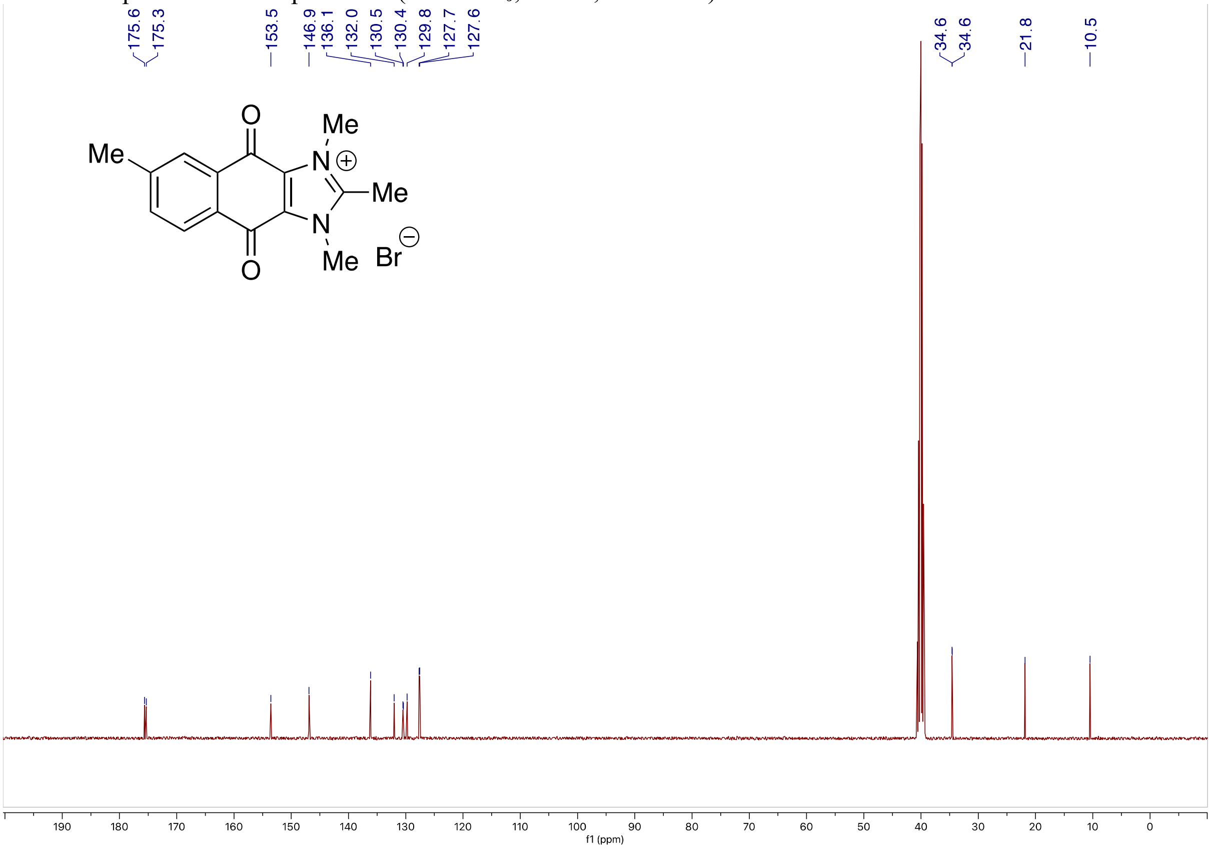

^{1}H NMR spectrum of compound **7f** (DMSO-d_6, 298 K, 400 MHz)

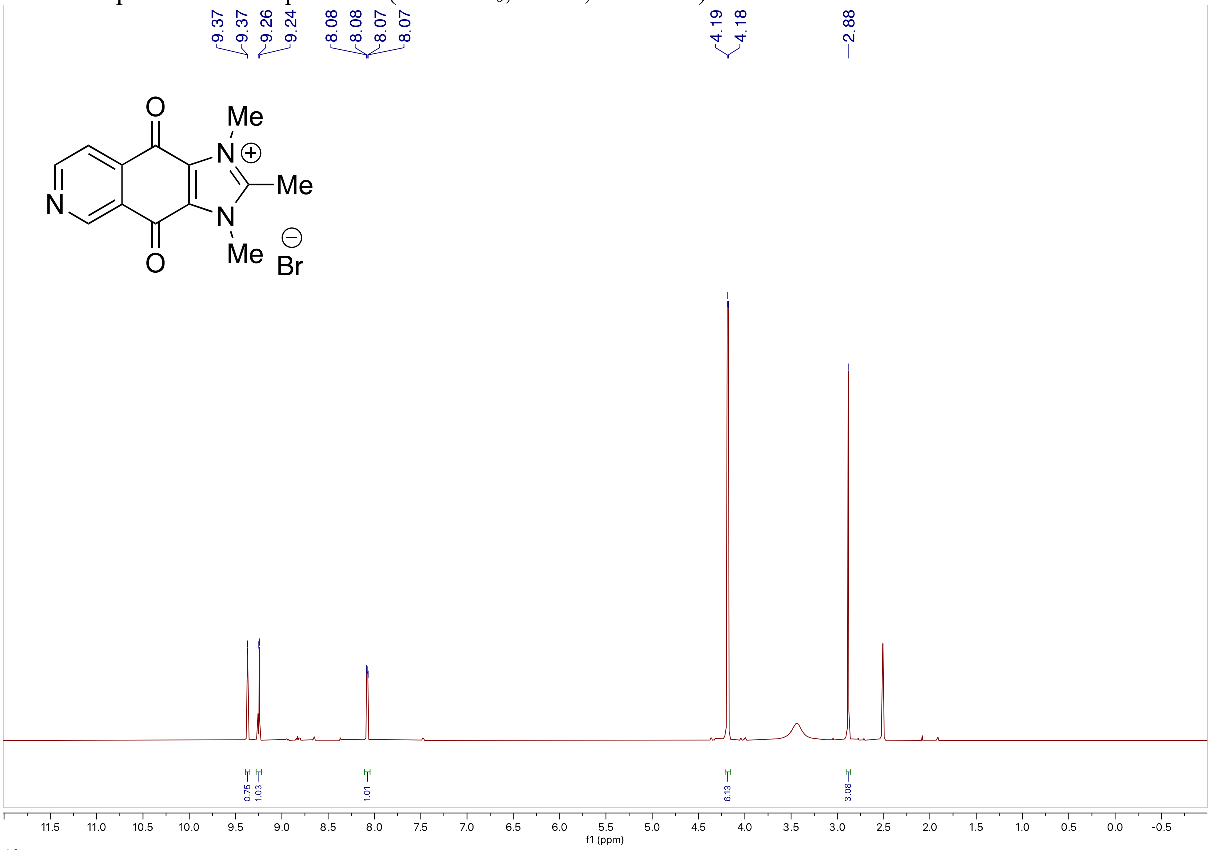

^{13}C NMR spectrum of compound **7f** (DMSO-d_6, 298 K, 101 MHz)

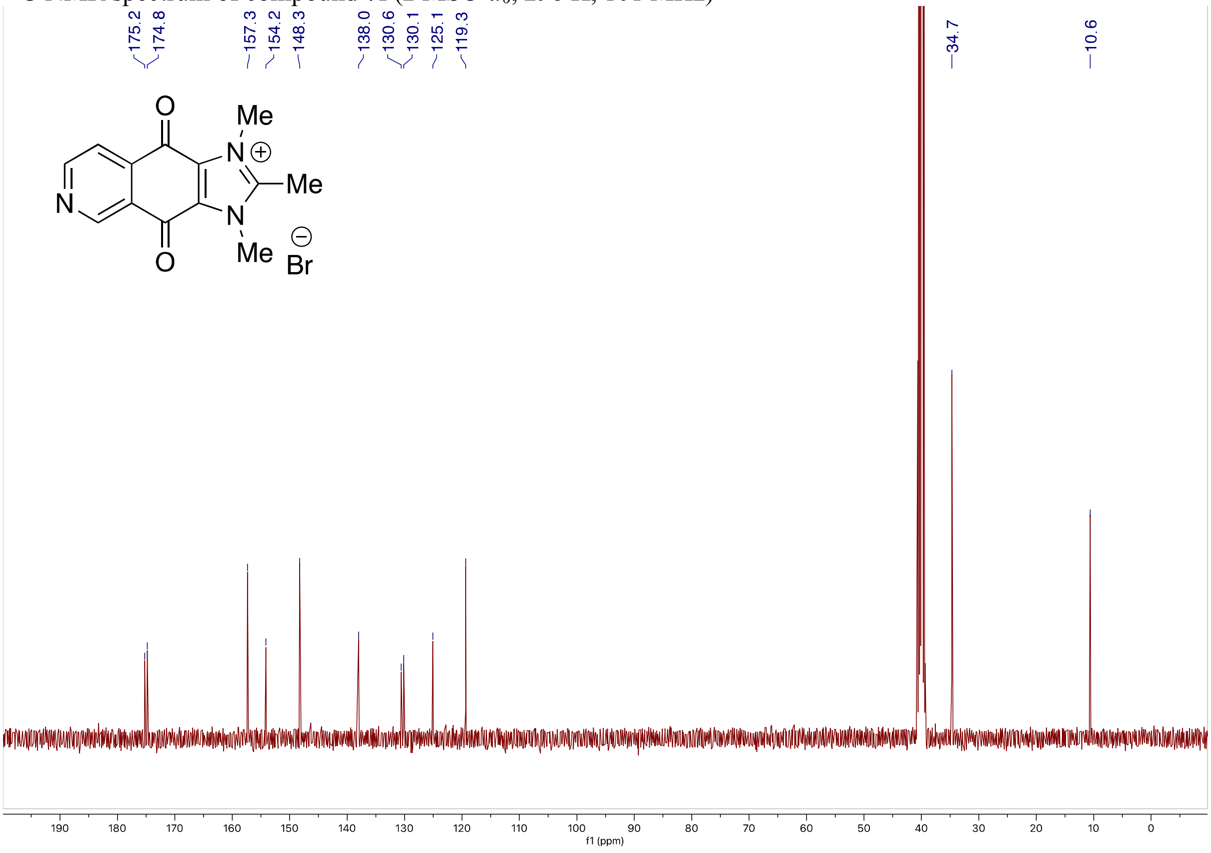

^{1}H NMR spectrum of compound **9** (DMSO-d_6, 298 K, 400 MHz)

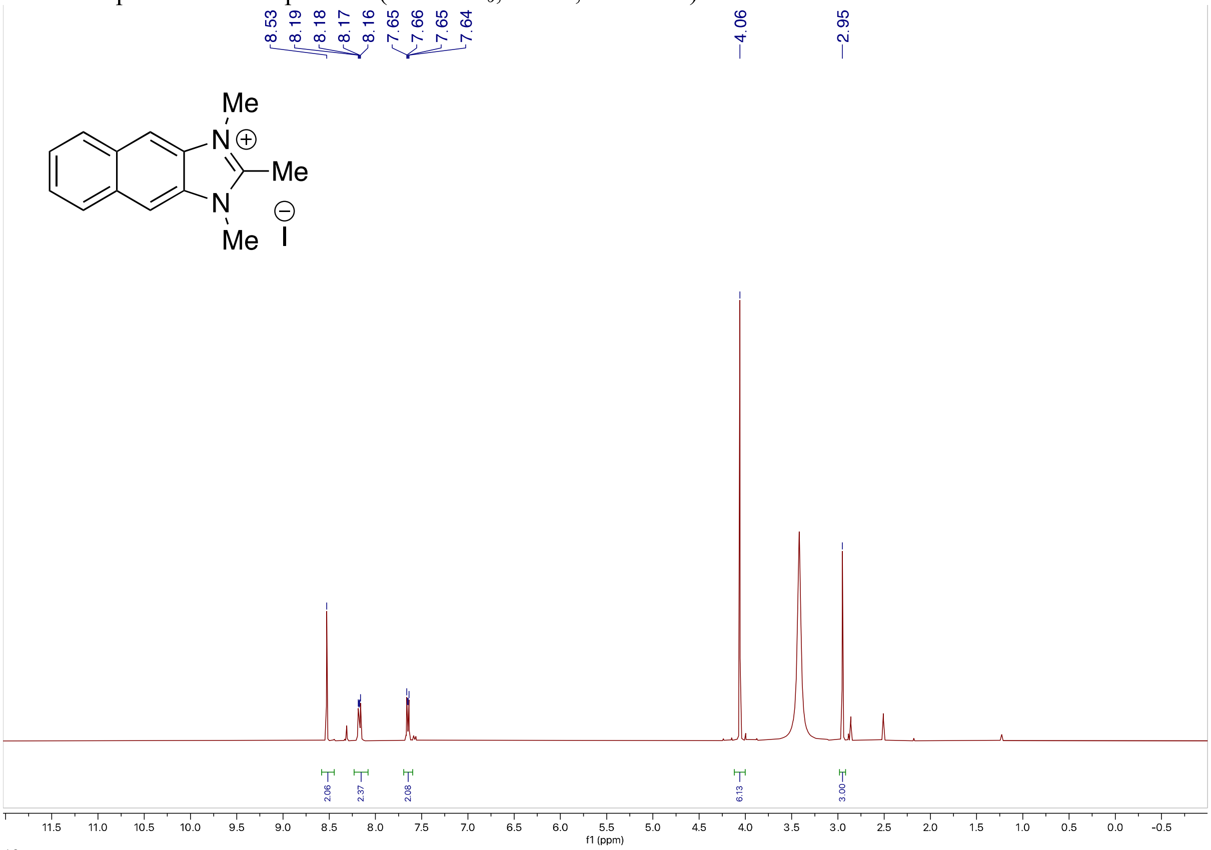

^{13}C NMR spectrum of compound **9** (DMSO-d_6, 298 K, 101 MHz)

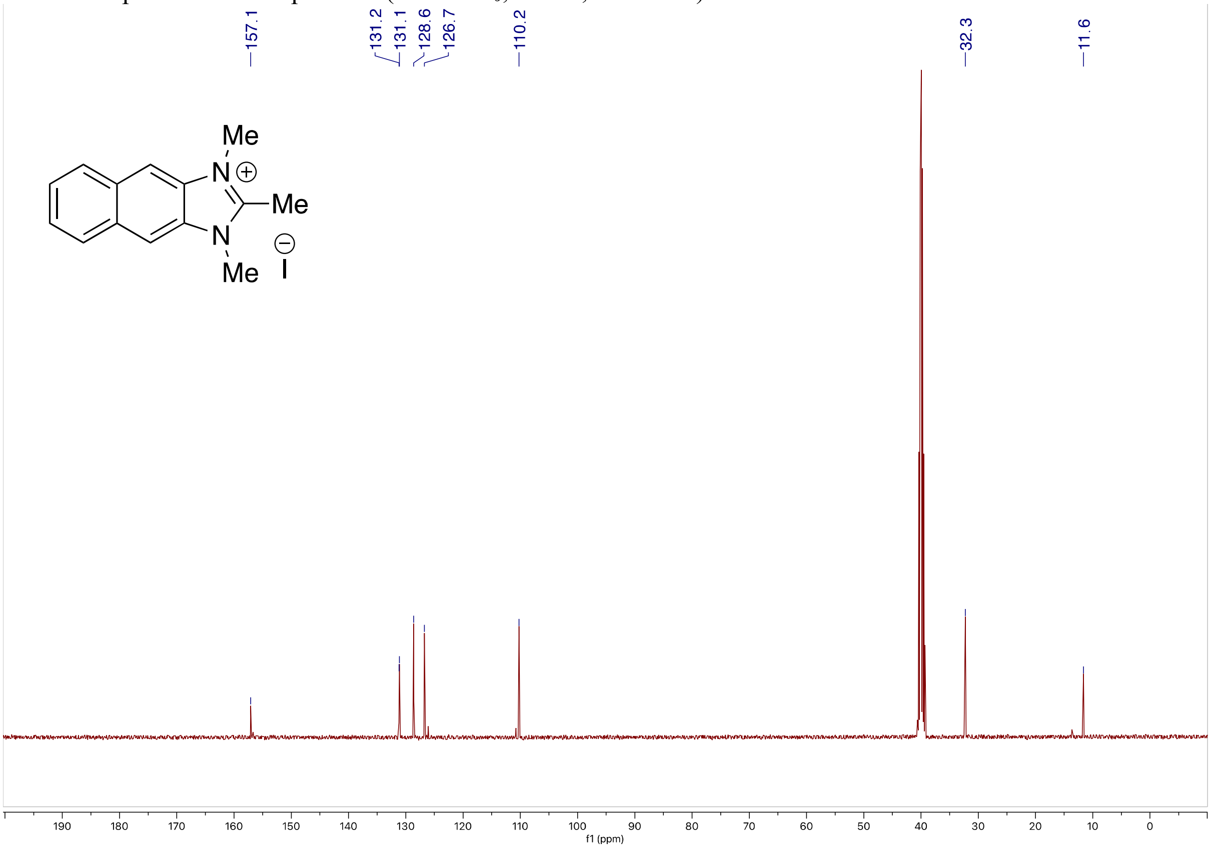

^{1}H NMR spectrum of compound **11** (DMSO-d_6, 298 K, 400 MHz)

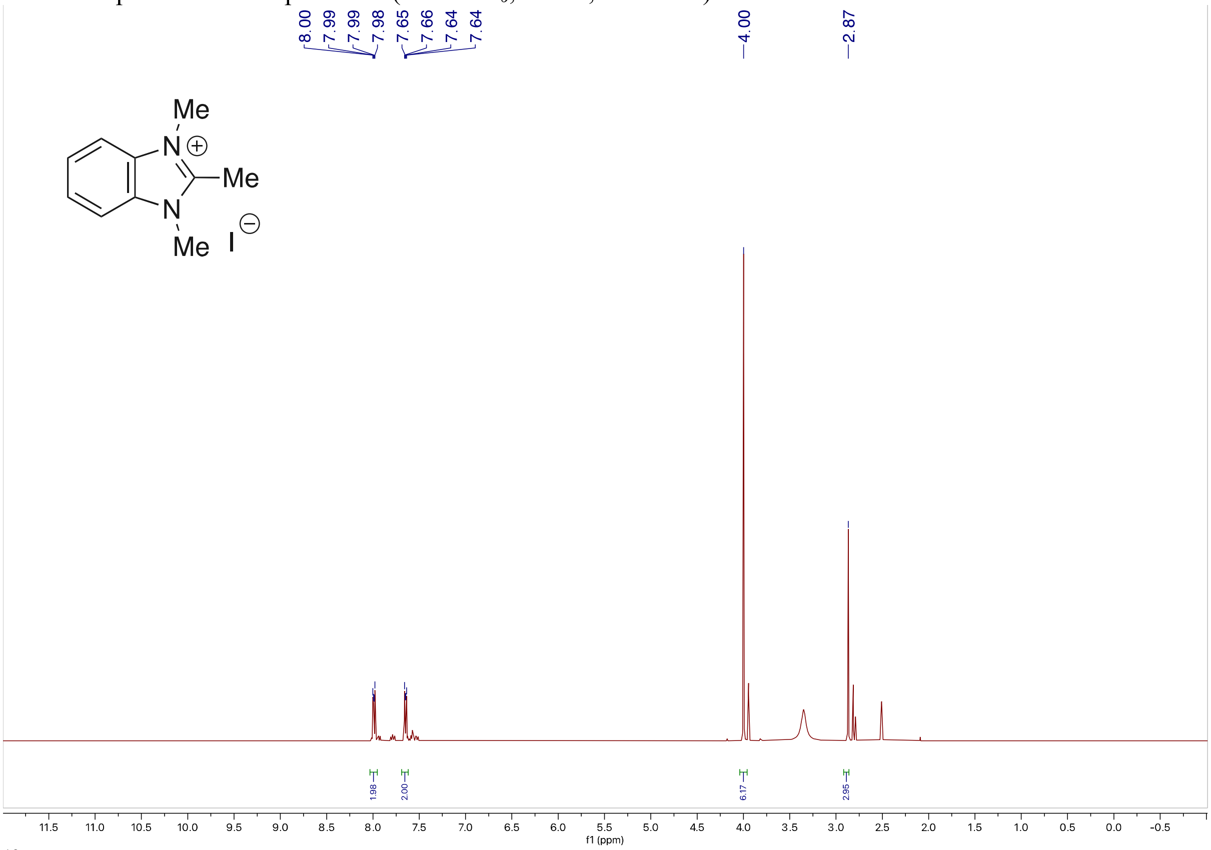

^{13}C NMR spectrum of compound **11** (DMSO-d_6, 298 K, 101 MHz)

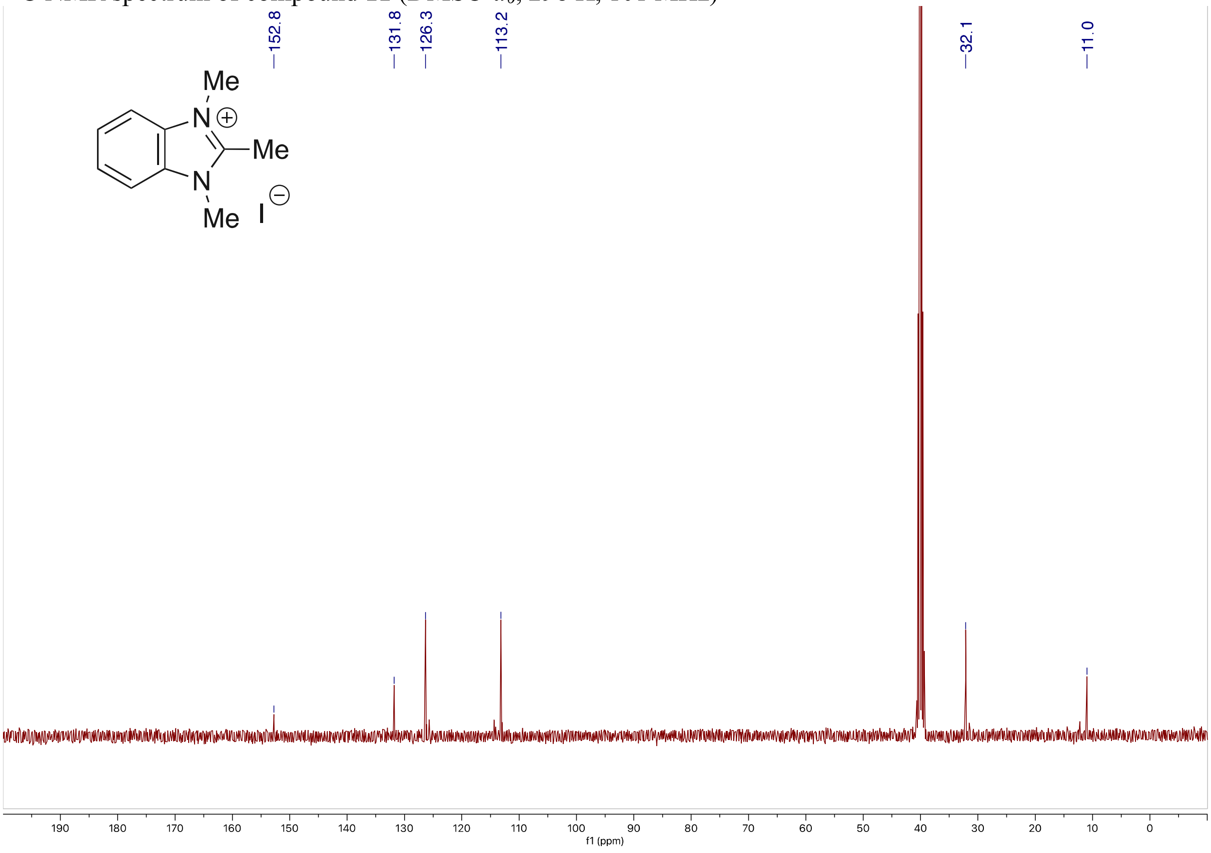

^{1}H NMR spectrum of compound **13** (DMSO-d_6, 298 K, 400 MHz)

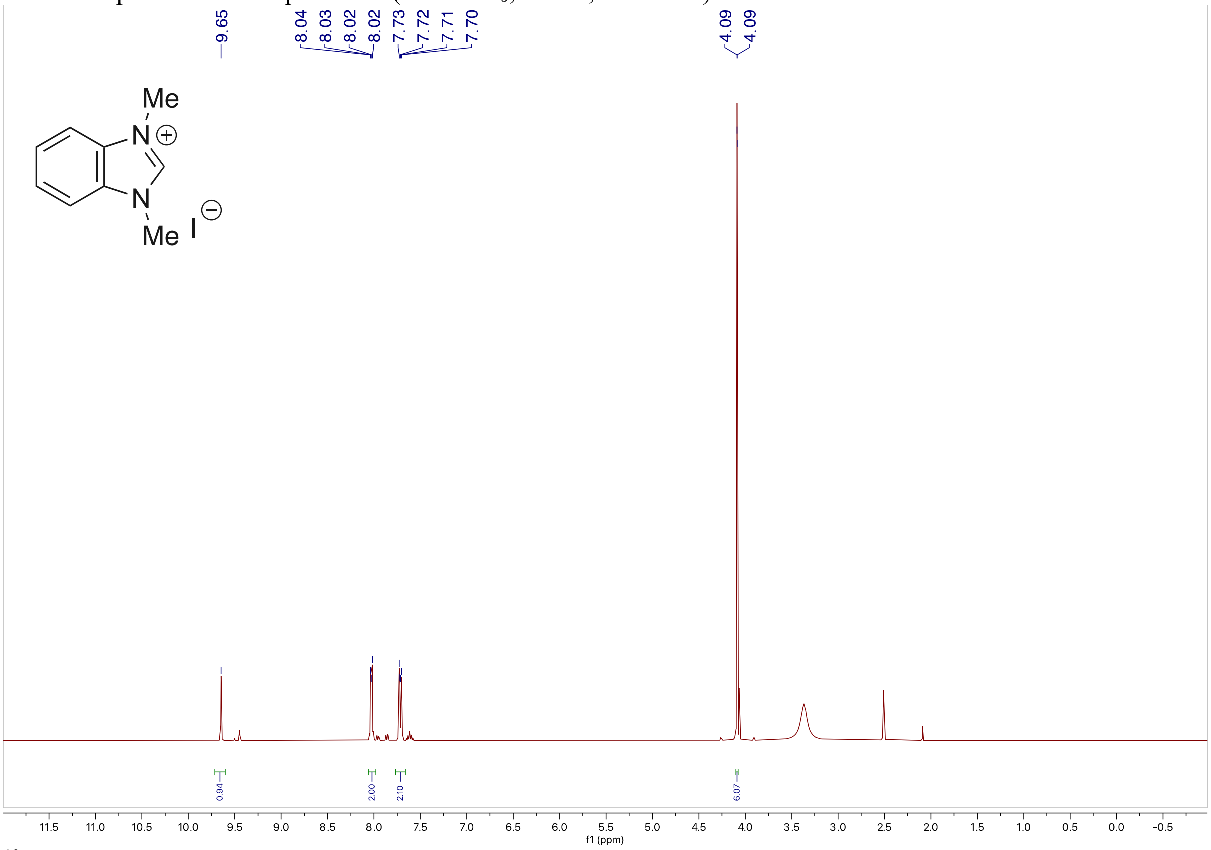

^{13}C NMR spectrum of compound **13** (DMSO-d_6, 298 K, 101 MHz)

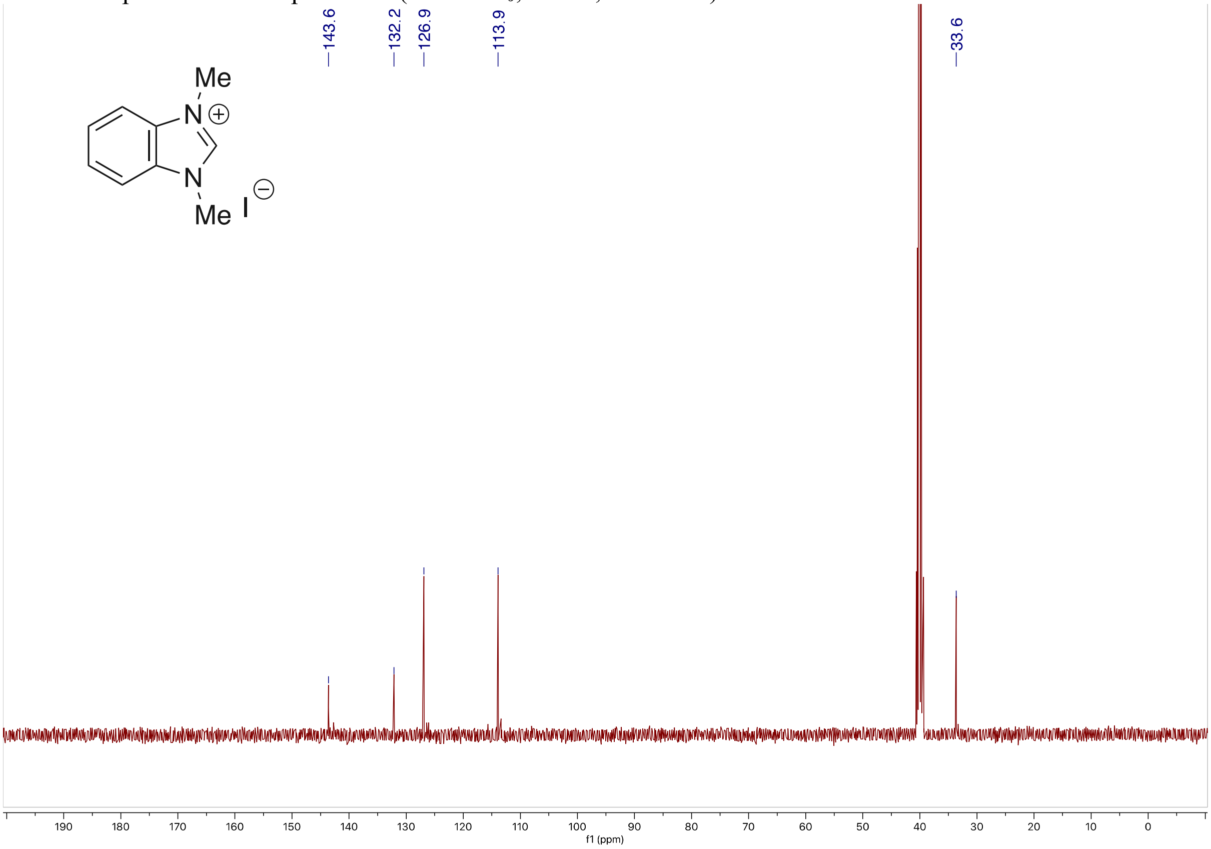

^{1}H NMR spectrum of compound **15** (DMSO-d_6, 298 K, 400 MHz)

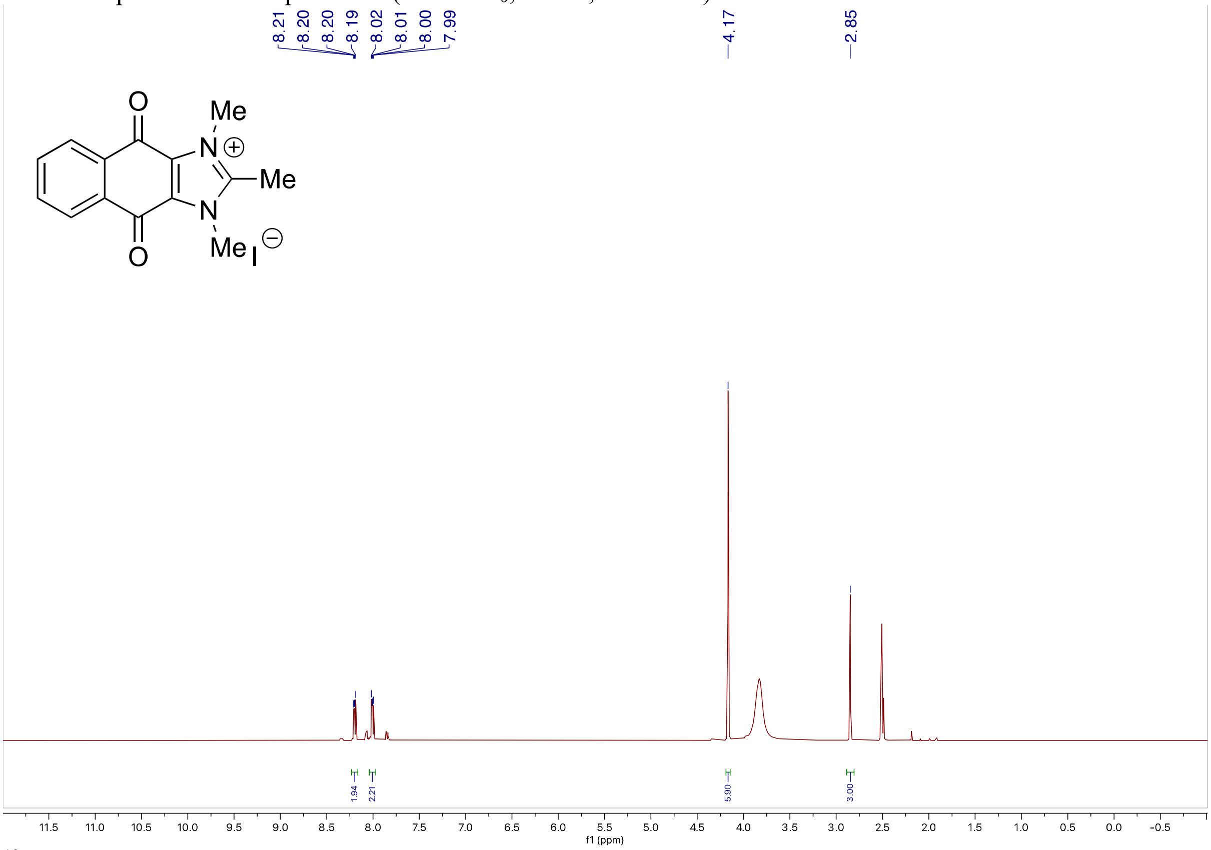

^{13}C NMR spectrum of compound **15** (DMSO-d_6, 298 K, 101 MHz)

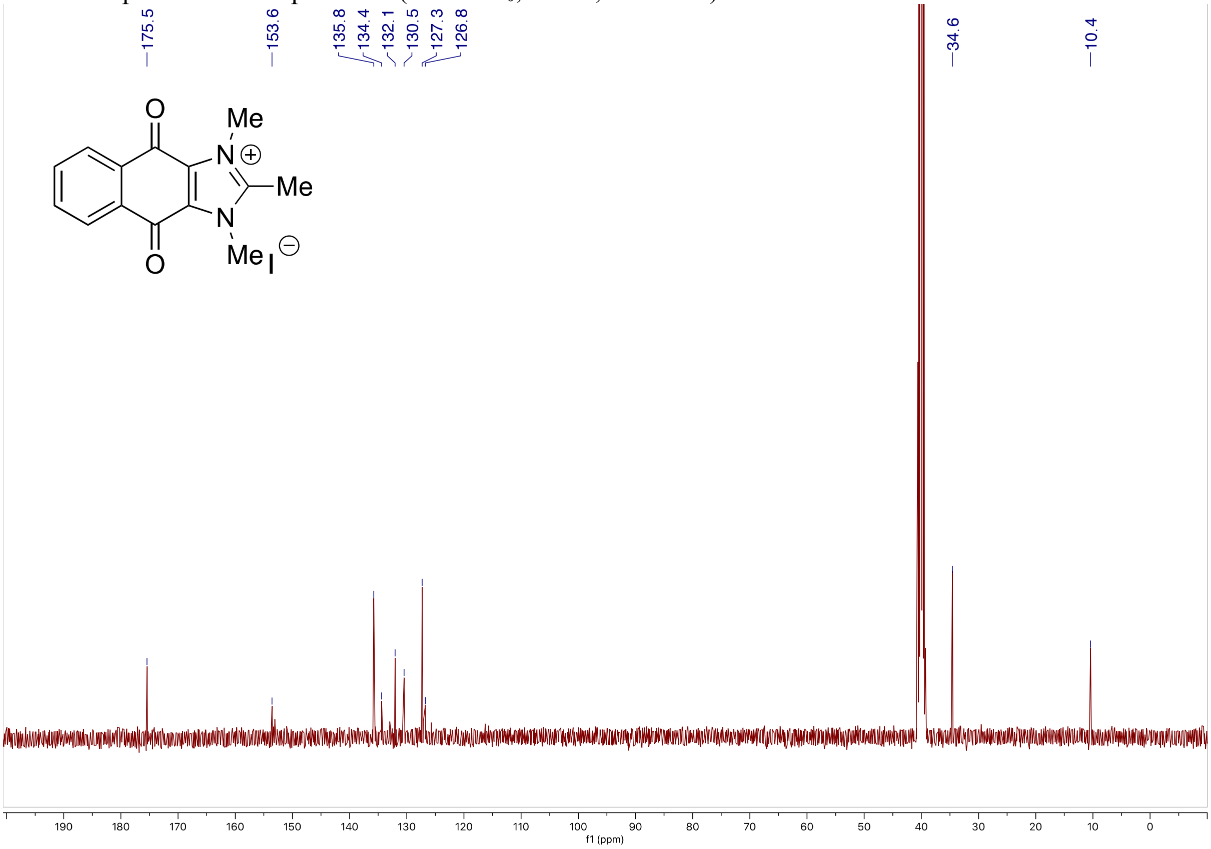

^{1}H NMR spectrum of compound **17a** (DMSO-d_6, 298 K, 400 MHz)

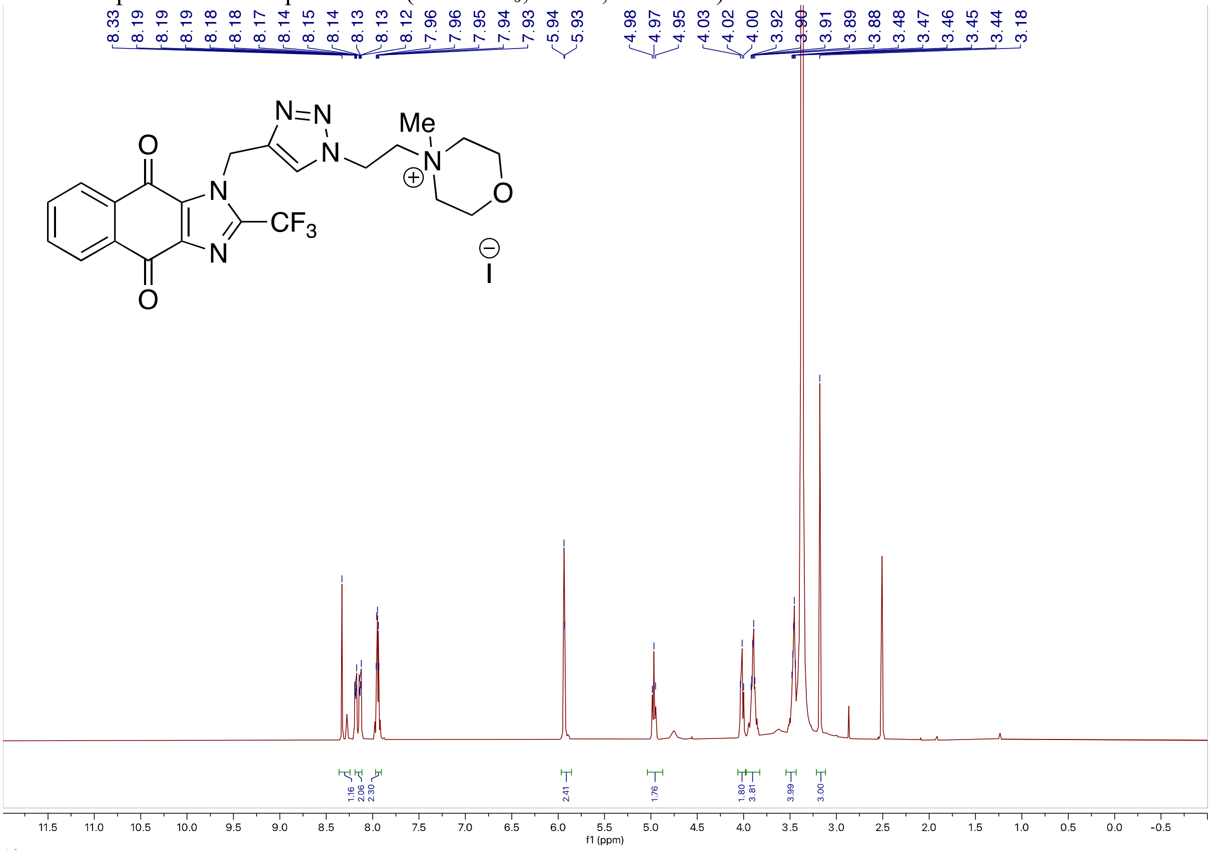

^{13}C NMR spectrum of compound **17a** (DMSO-d_6, 298 K, 101 MHz)

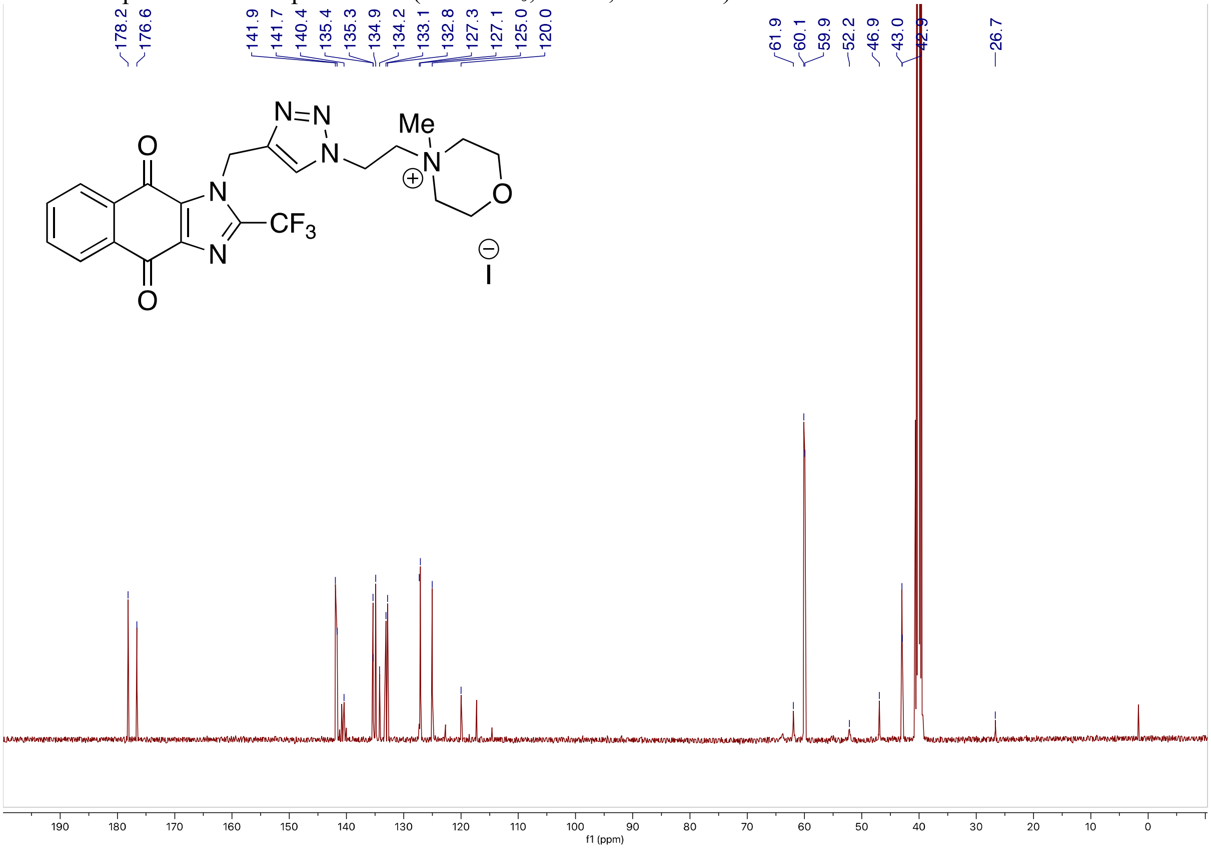

^{1}H NMR spectrum of compound **17b** (DMSO-d_6, 298 K, 400 MHz)

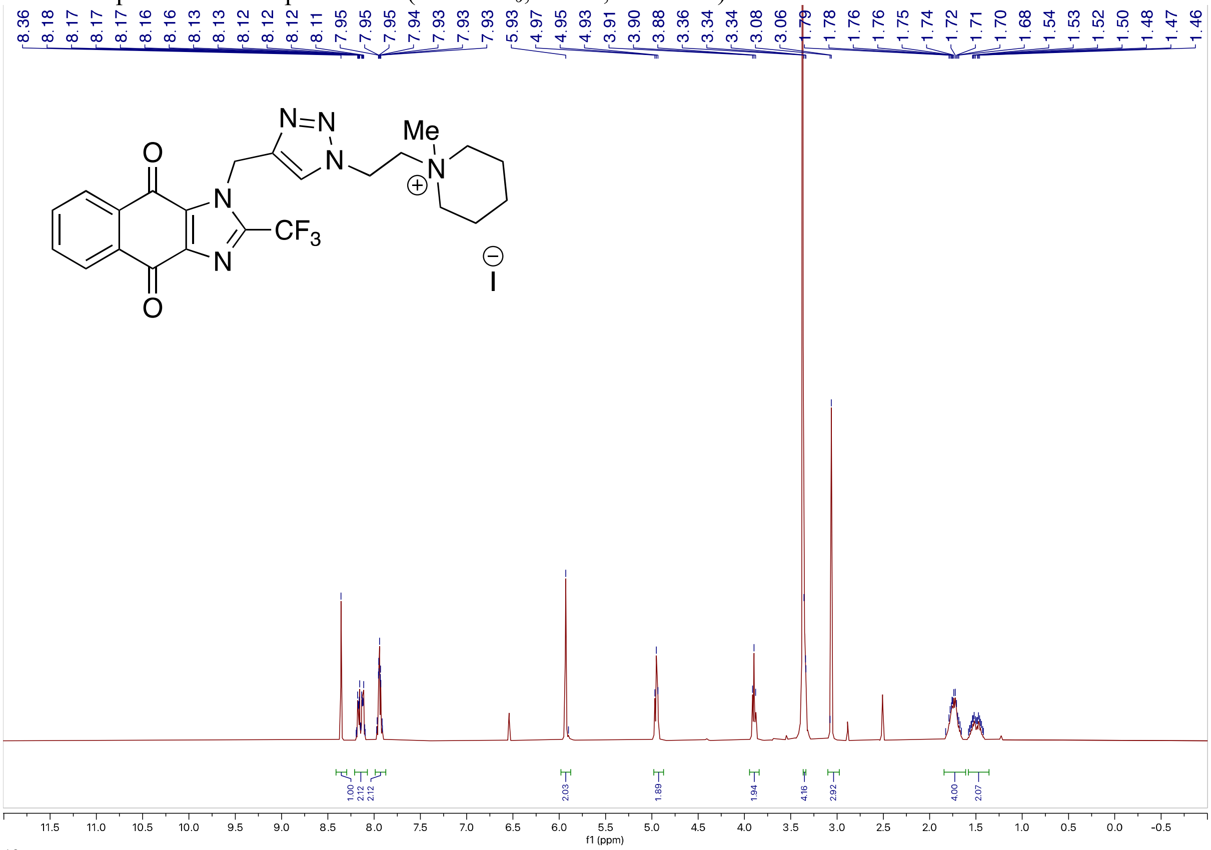

^{13}C NMR spectrum of compound **17b** (DMSO-d_6, 298 K, 101 MHz)

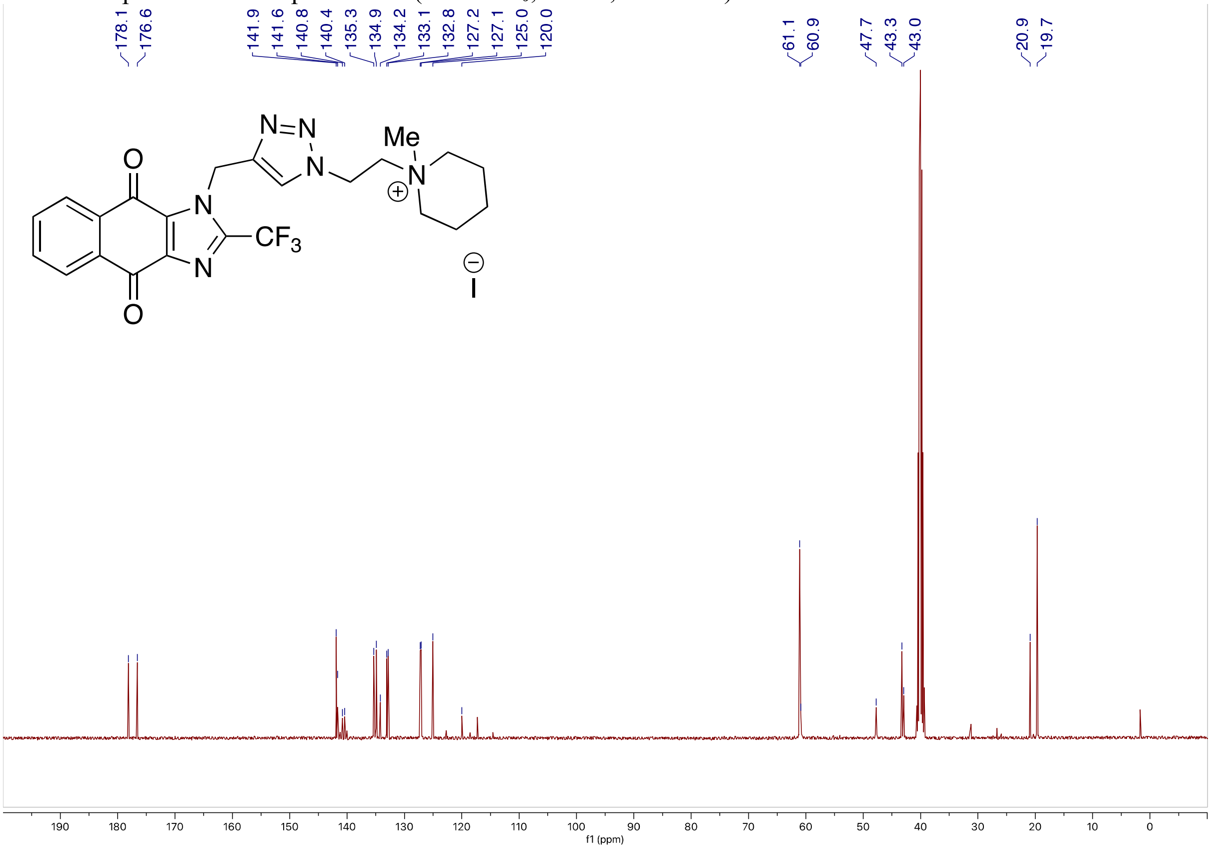

^{1}H NMR spectrum of compound **17c** (DMSO-d_6, 298 K, 400 MHz)

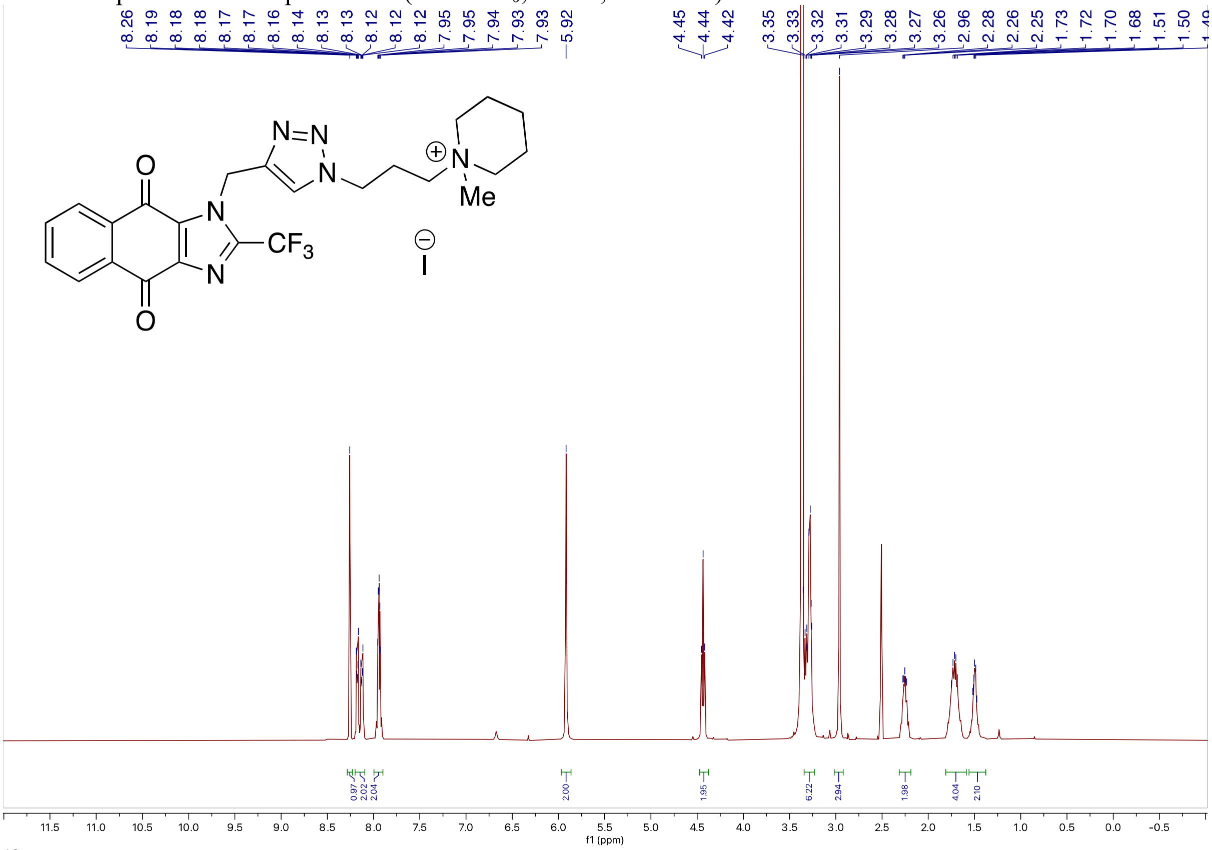

^{13}C NMR spectrum of compound **17c** (DMSO-d_6, 298 K, 101 MHz)

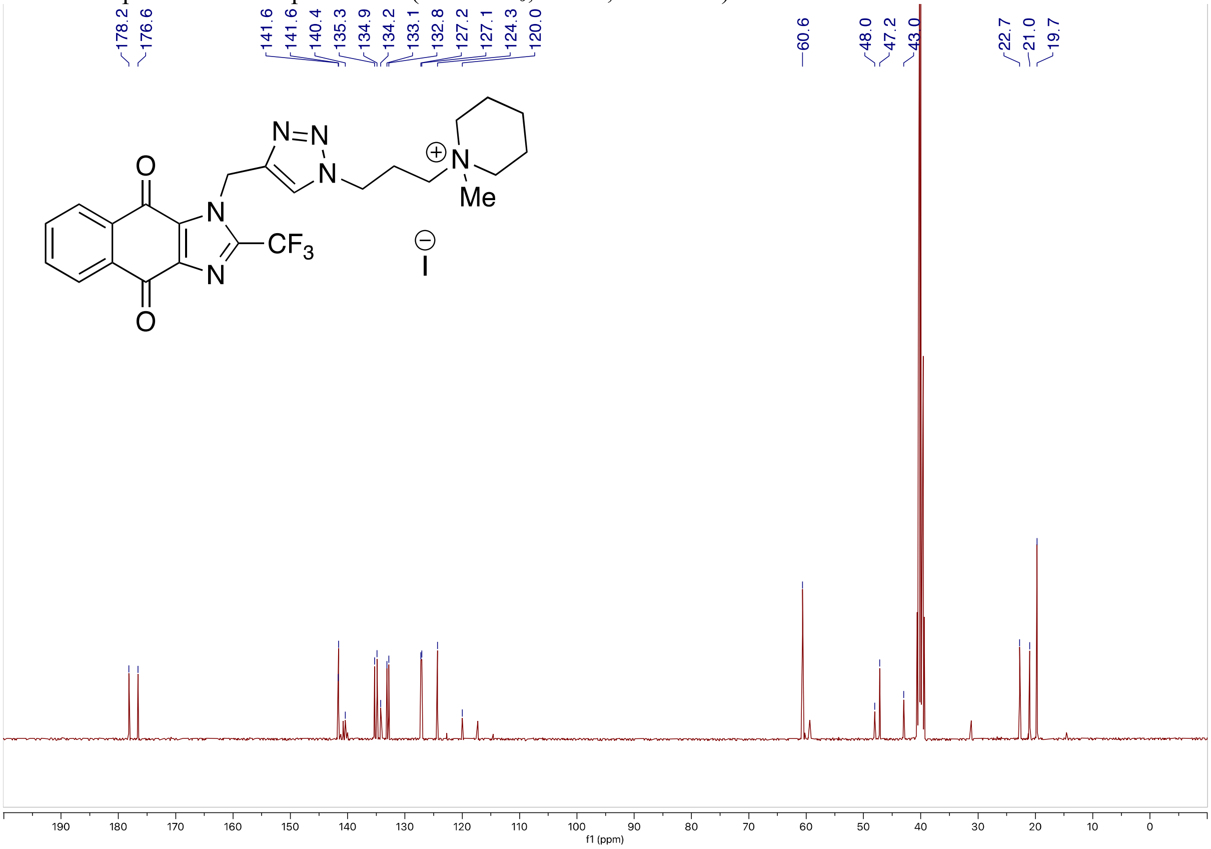

^{1}H NMR spectrum of compound **19** (DMSO-d_6, 298 K, 400 MHz)

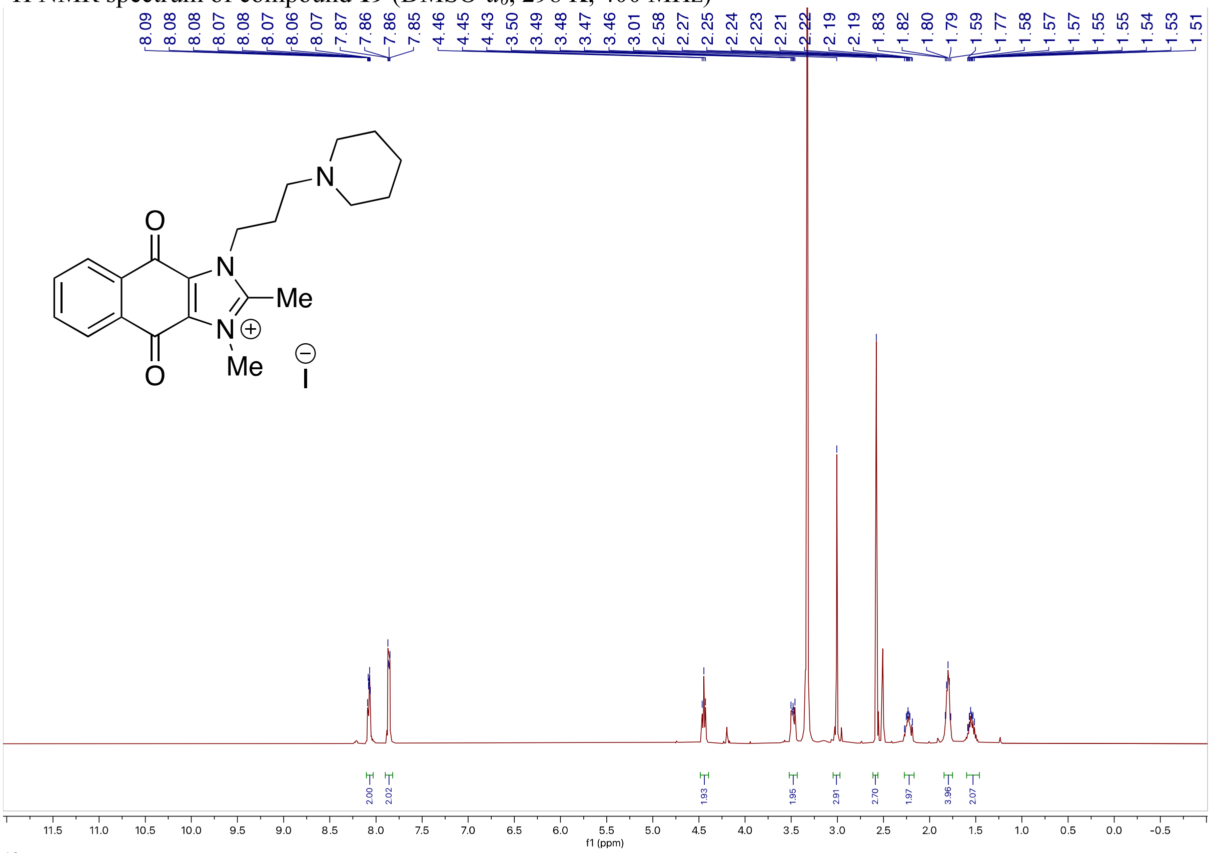

^{13}C NMR spectrum of compound **19** (DMSO-d_6, 298 K, 101 MHz)

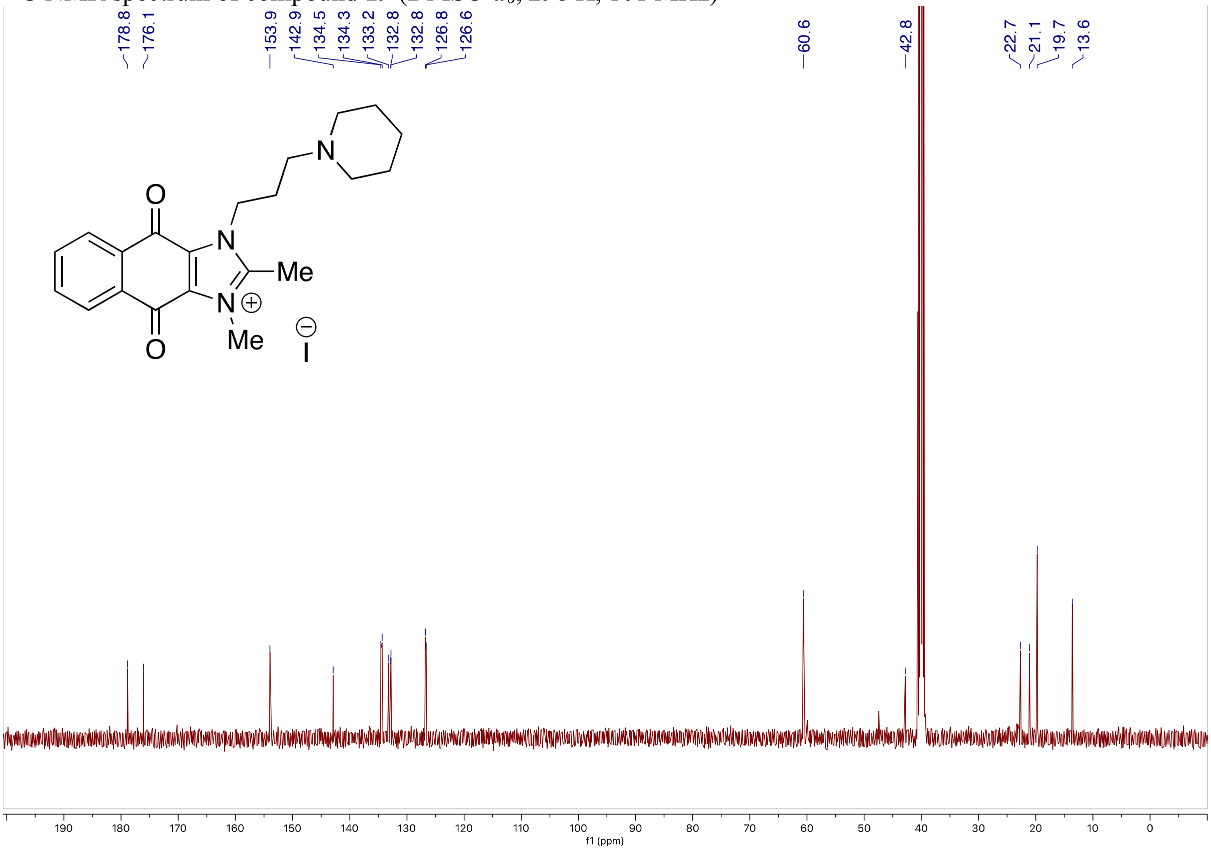

MS Spectra

HRMS spectrum of compound **2a**

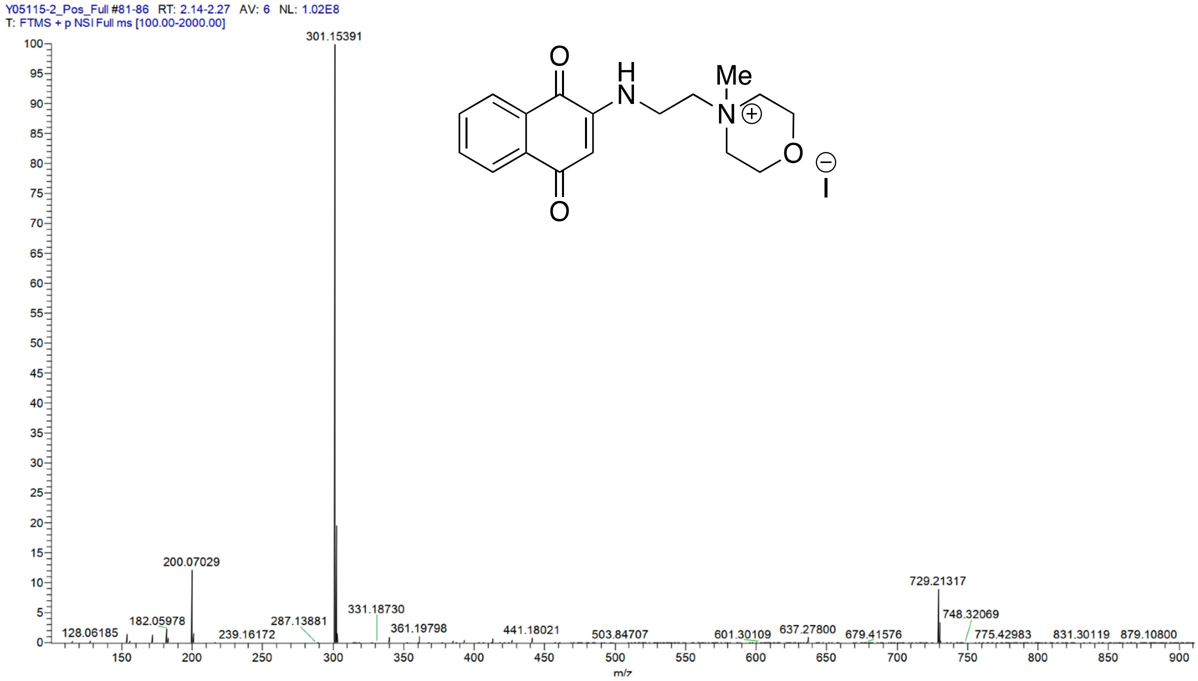

HRMS spectrum of compound **2b**

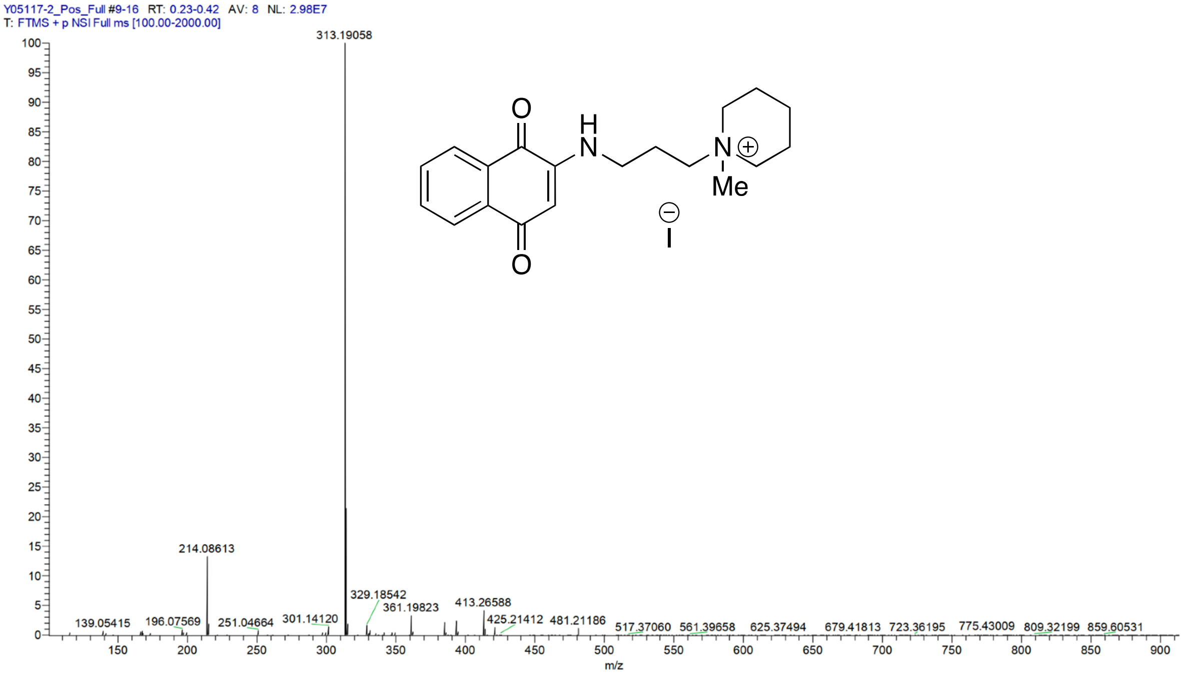

HRMS spectrum of compound **2c**

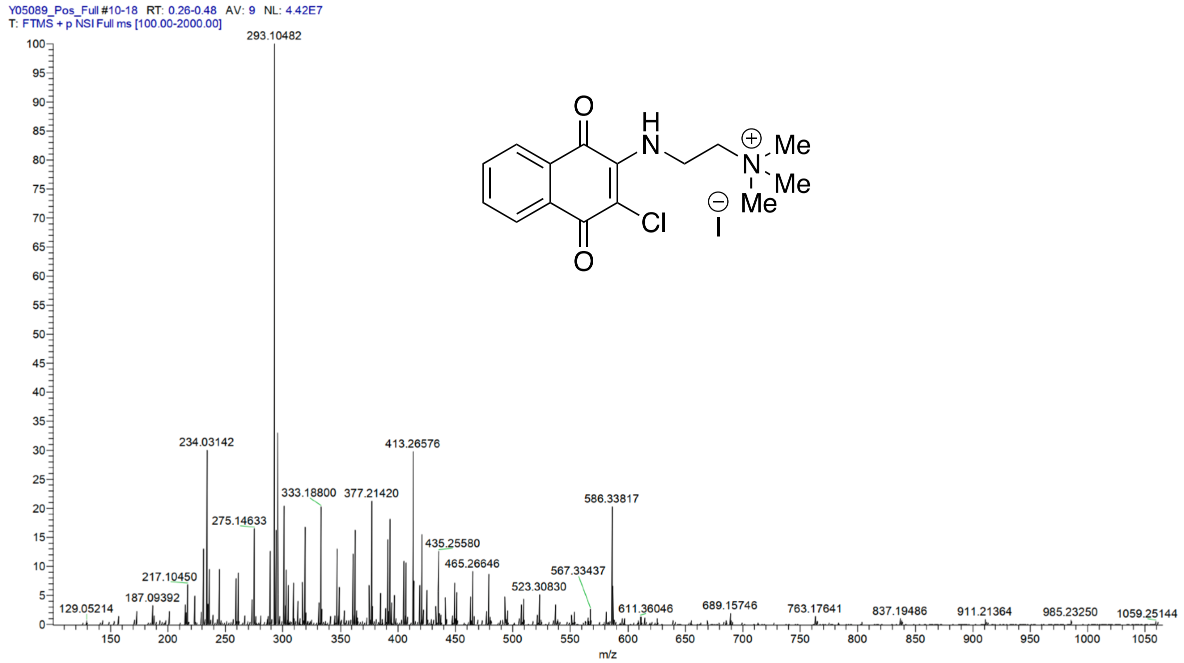

HRMS spectrum of compound **2d**

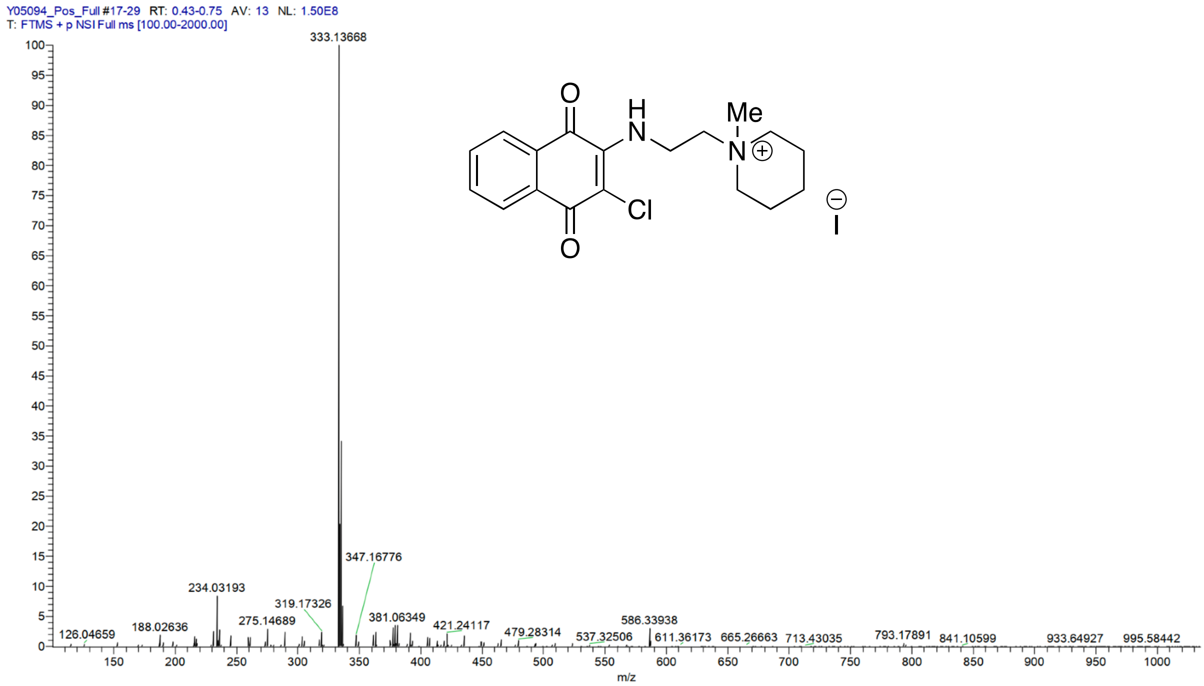

HRMS spectrum of compound **2e**

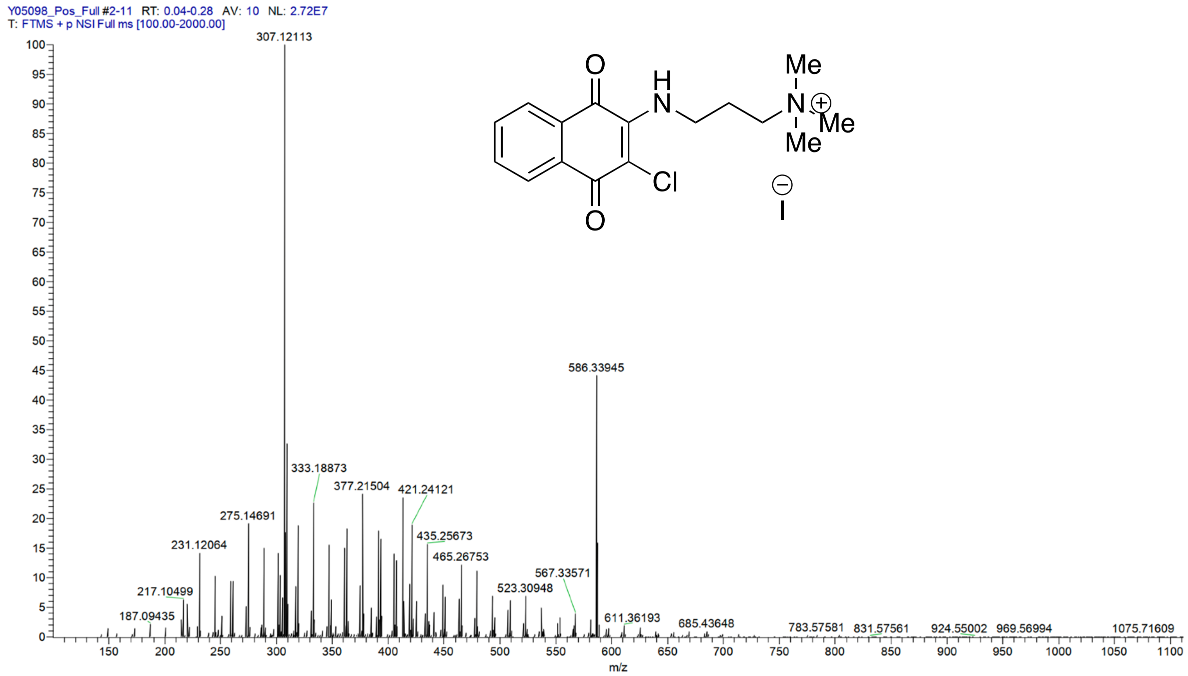

HRMS spectrum of compound **2f**

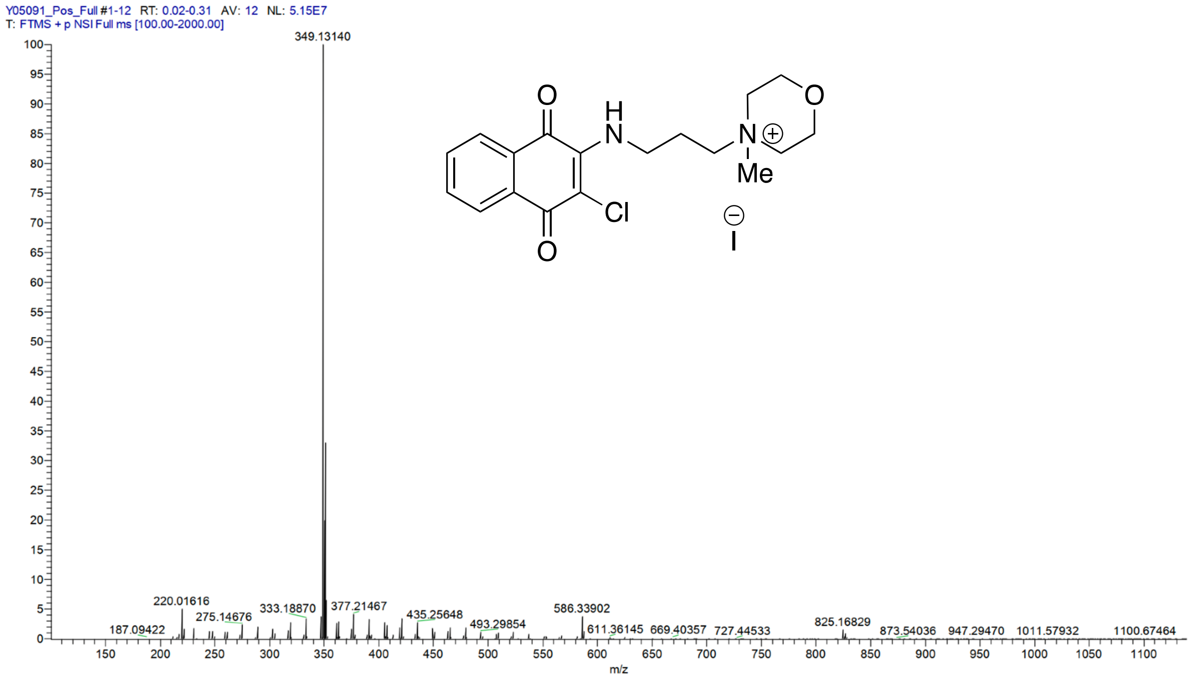

HRMS spectrum of compound **2g**

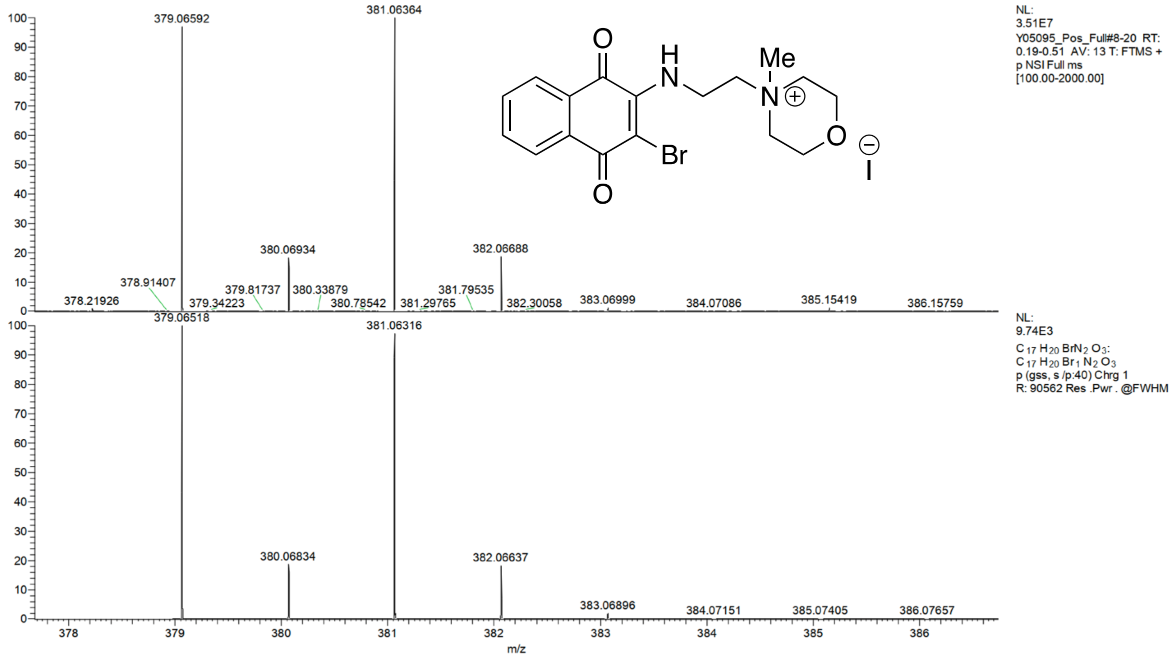

HRMS spectrum of compound **2h**

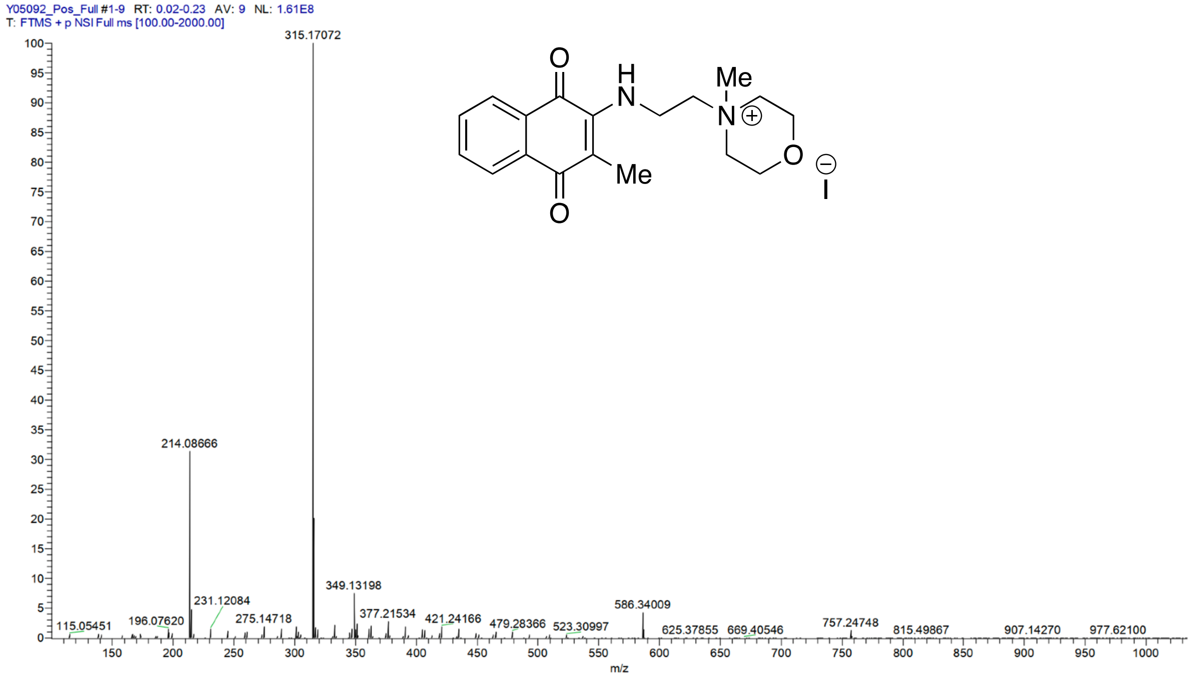

MS spectrum of compound **7a**

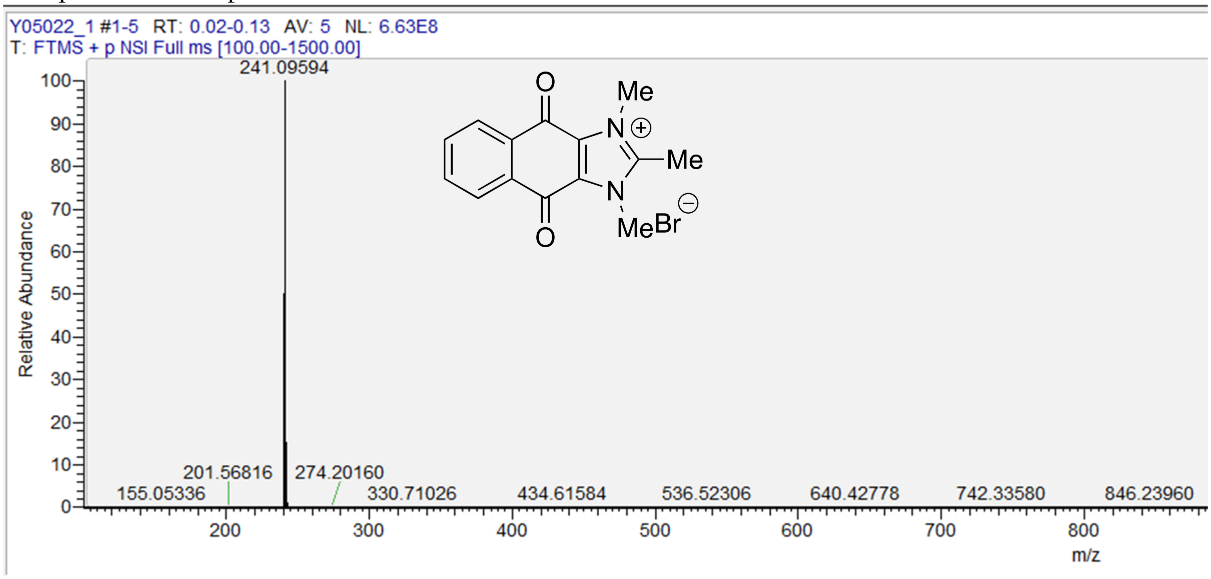

HRMS spectrum of compound **7b**

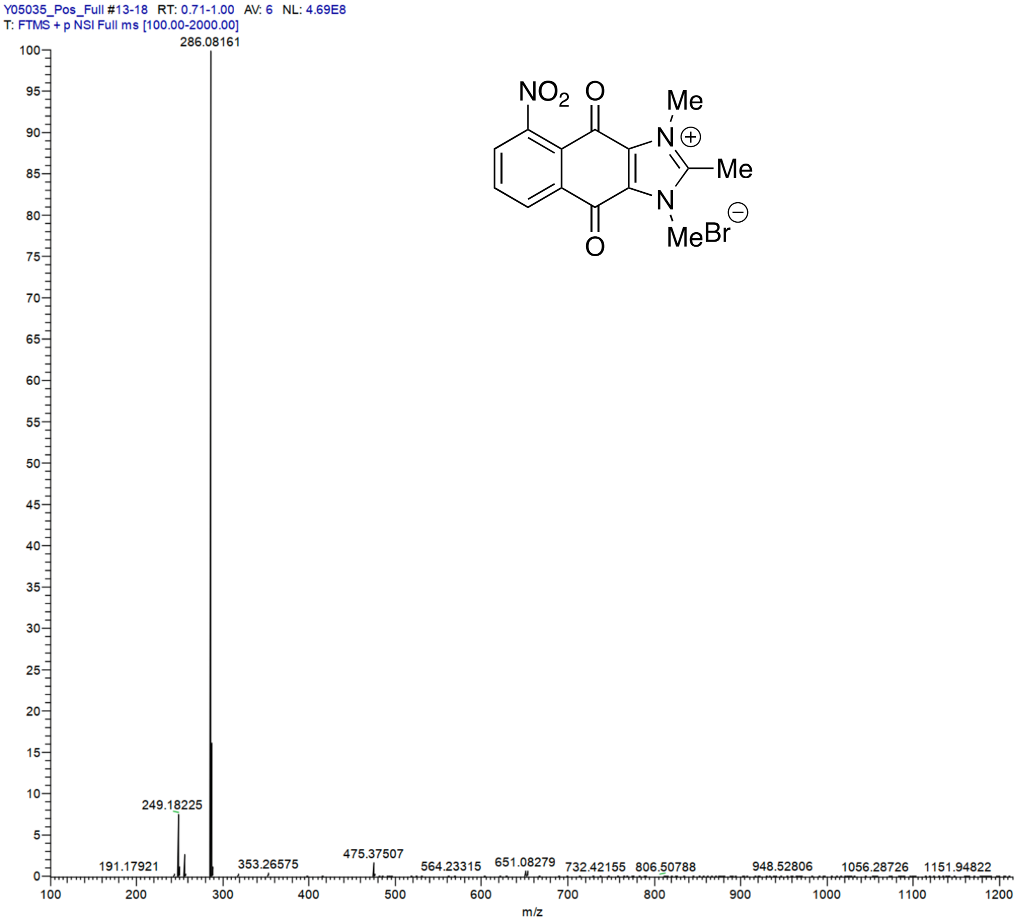

HRMS spectrum of compound **7c**

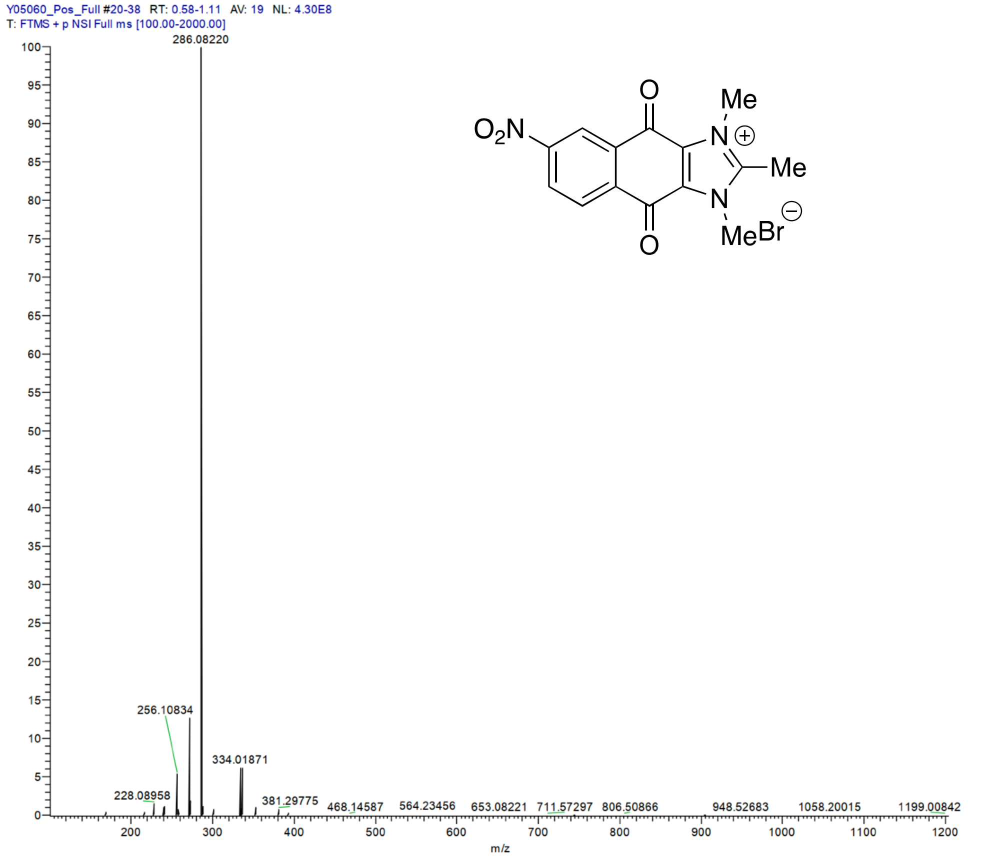

HRMS spectrum of compound **7d**

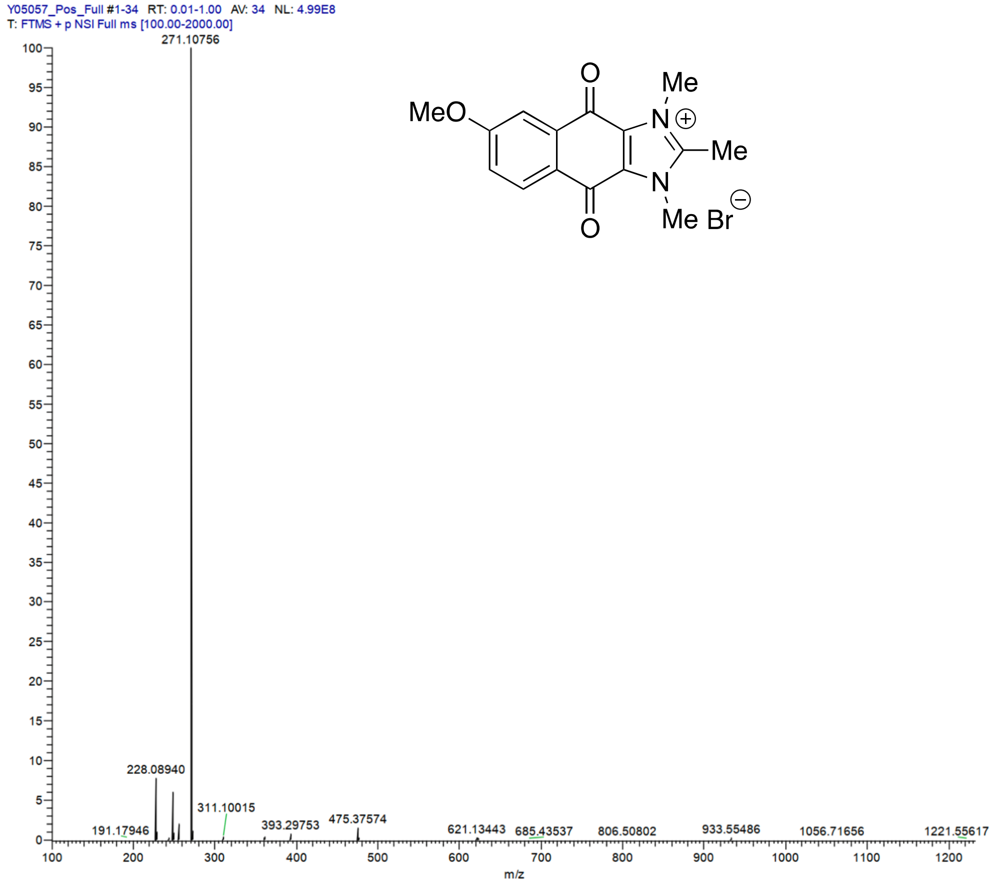

HRMS spectrum of compound **7e**

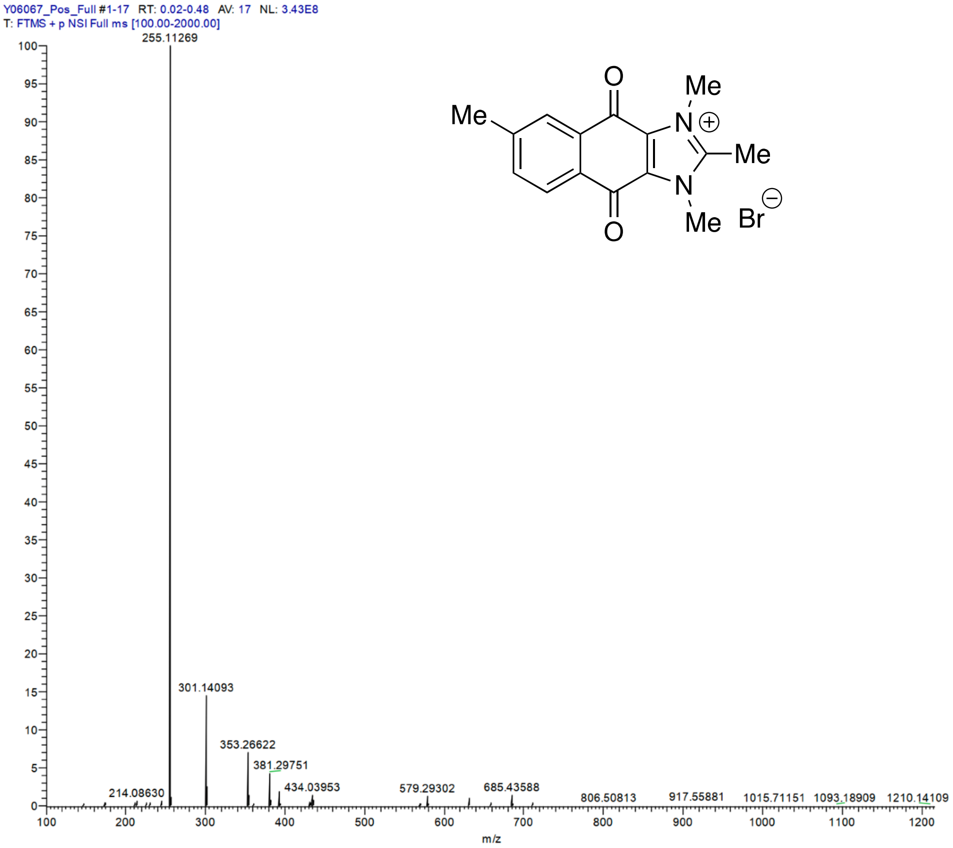

HRMS spectrum of compound **7f**

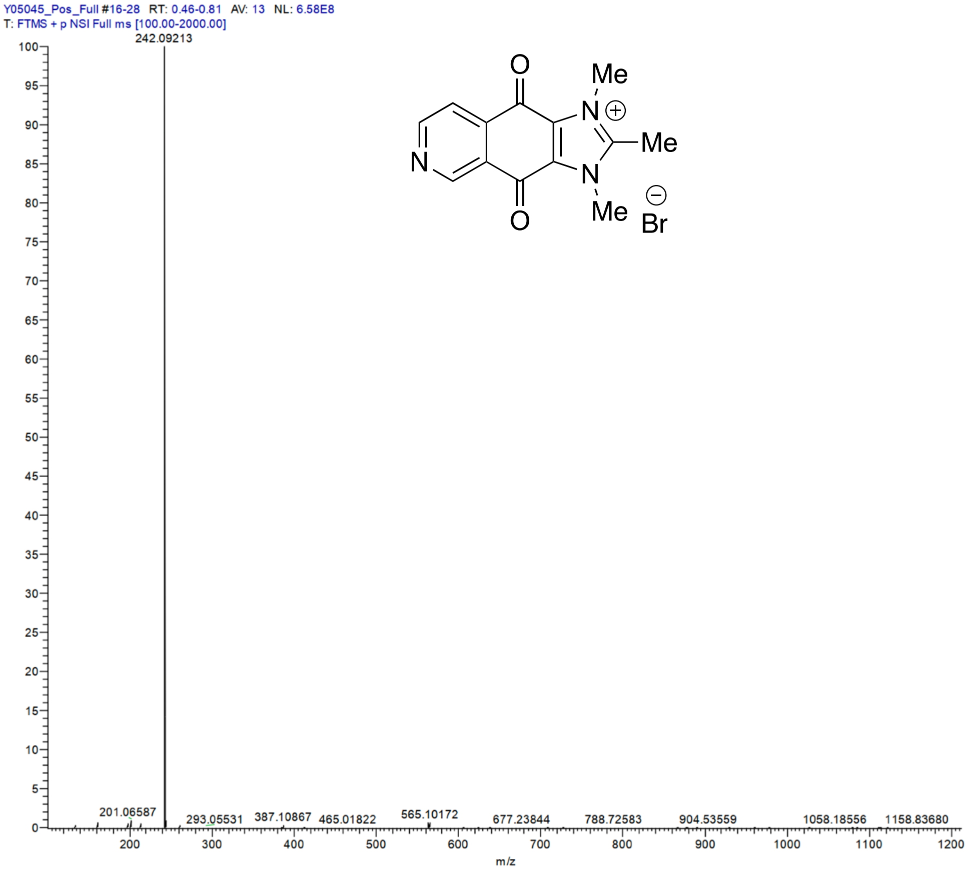

HRMS spectrum of compound **9**

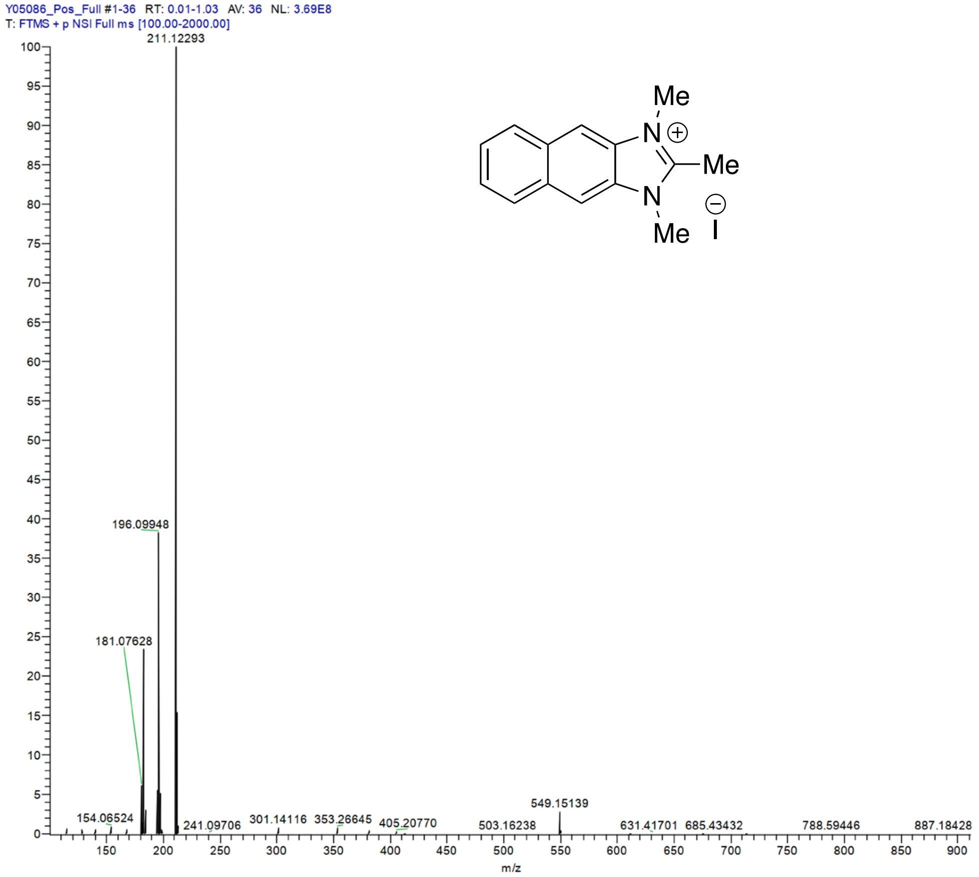

MS spectrum of compound **11**

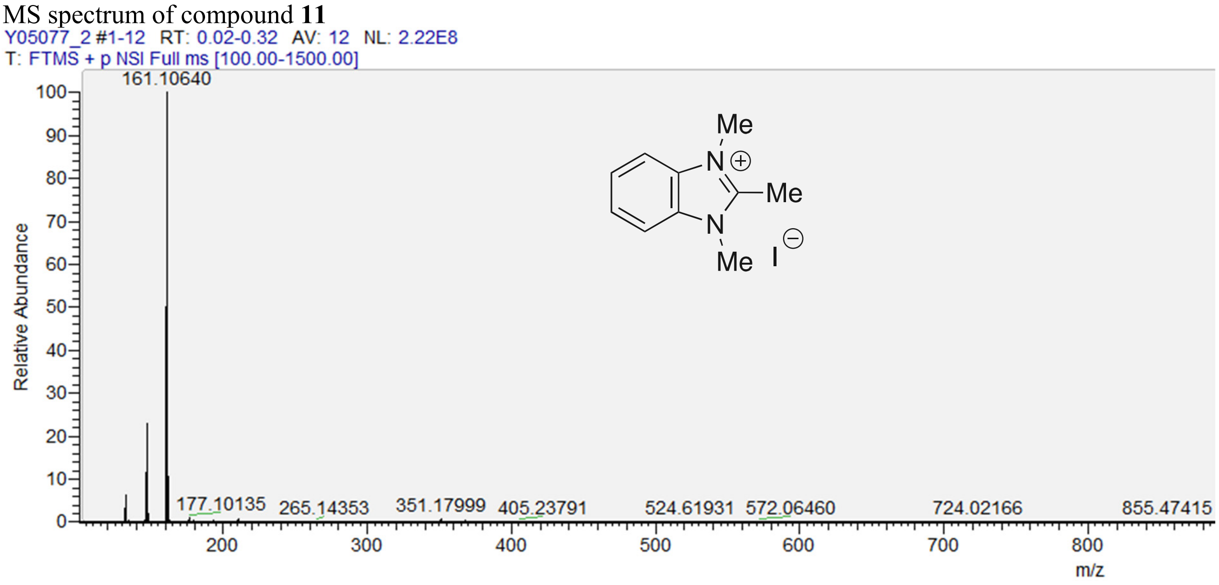

MS spectrum of compound **13**

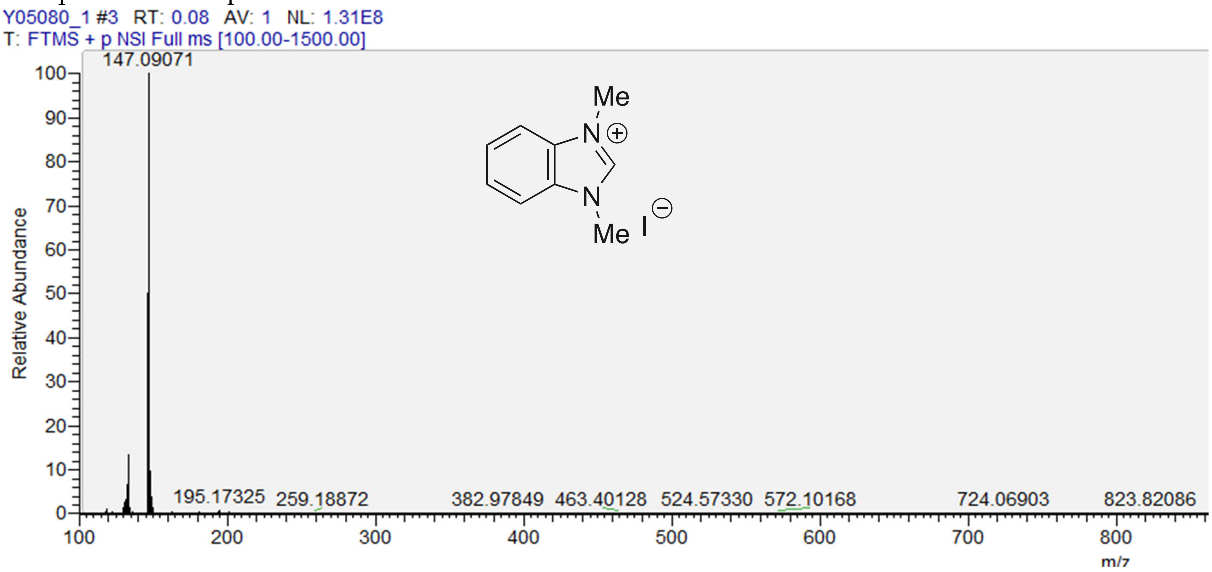

HRMS spectrum of compound **15**

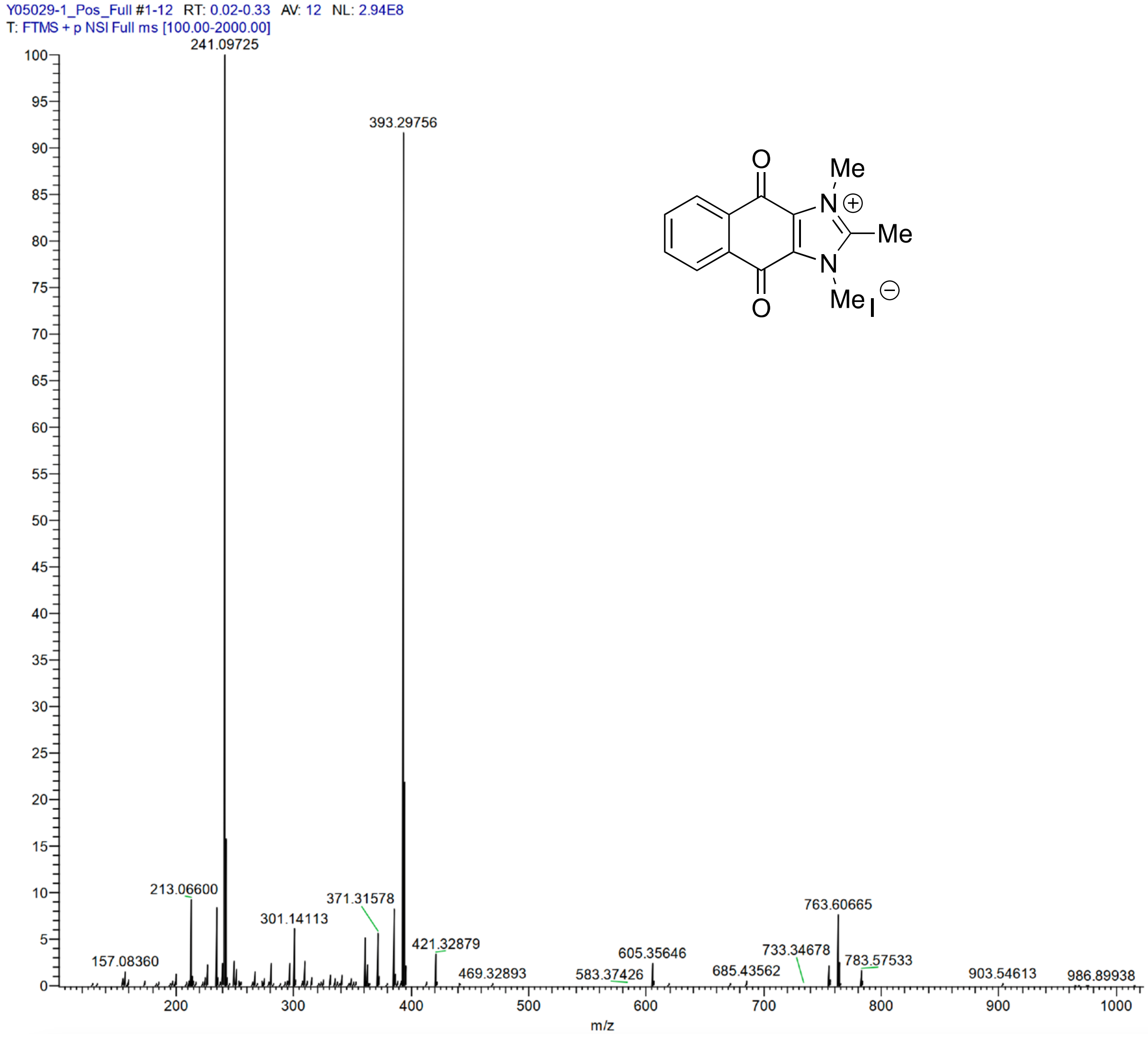

HRMS spectrum of compound **17a**

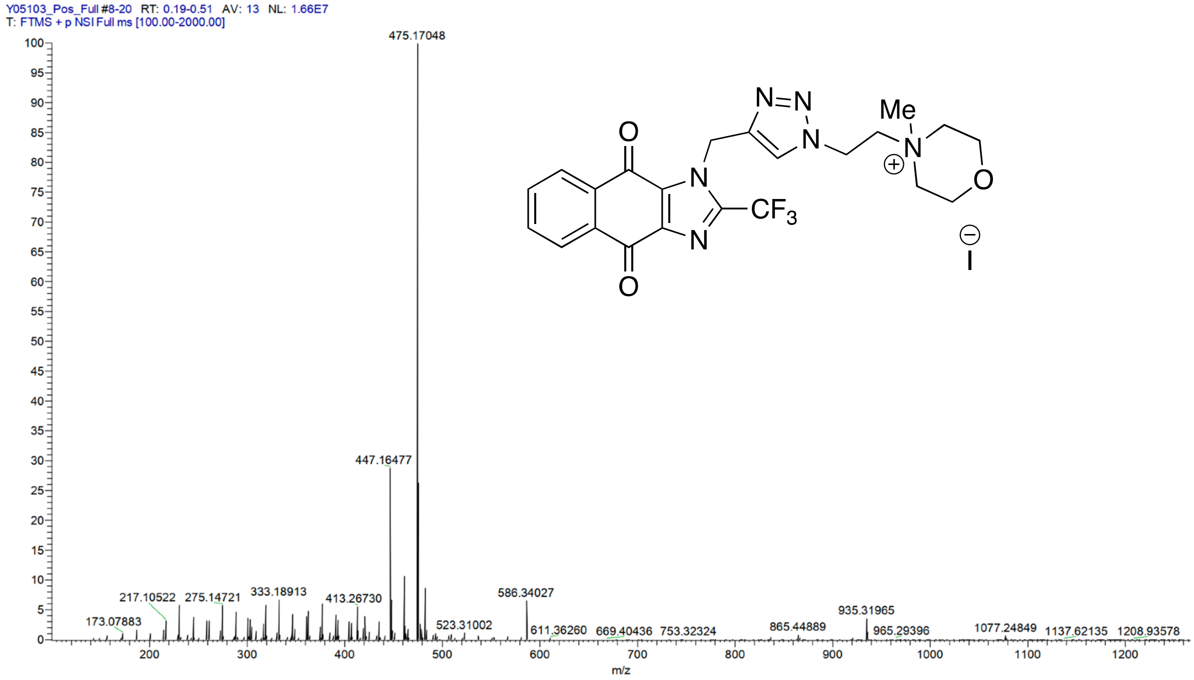

HRMS spectrum of compound **17b**

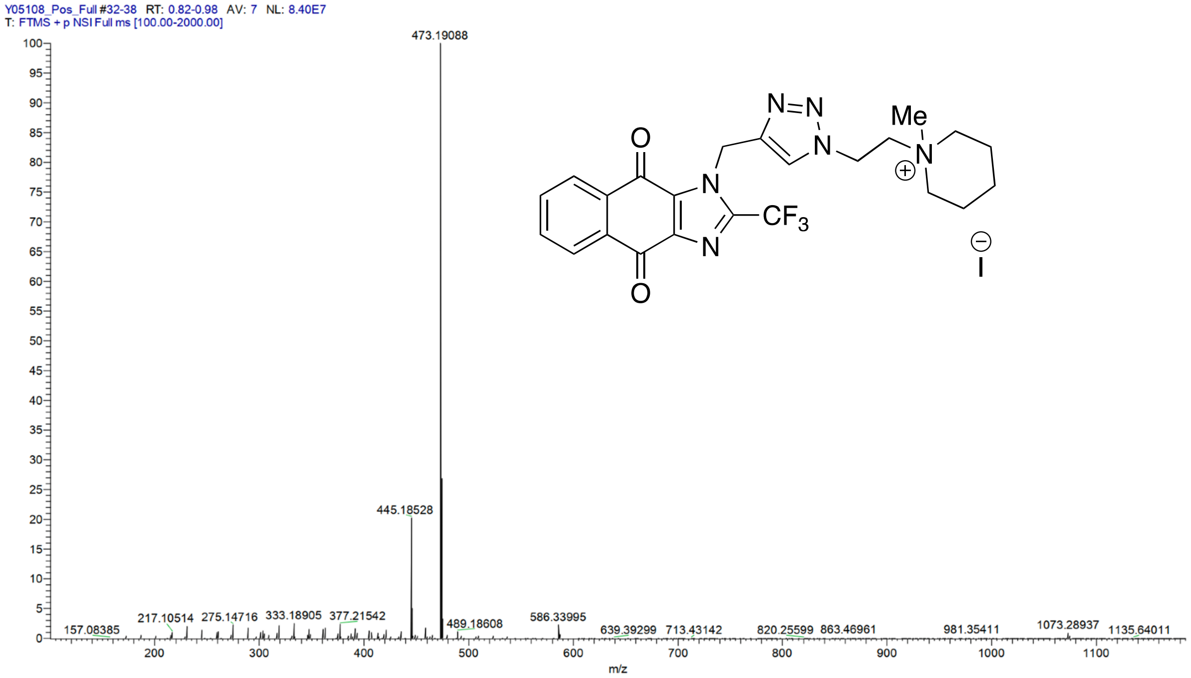

HRMS spectrum of compound **17c**

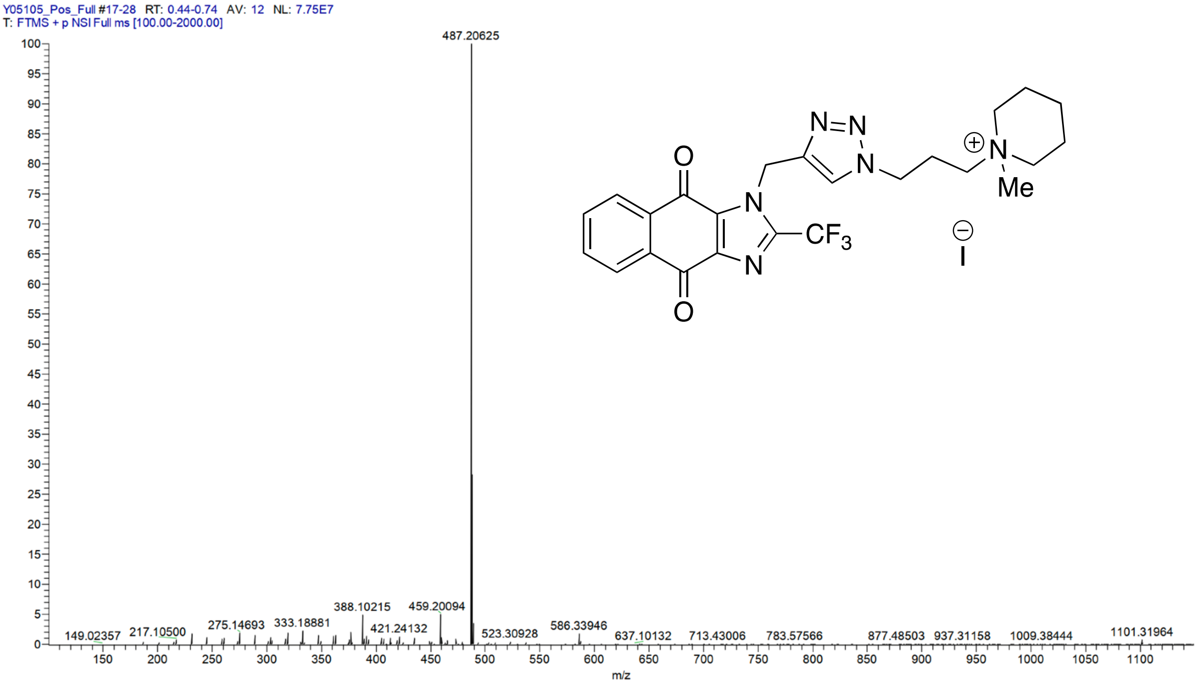

HRMS spectrum of compound **19**

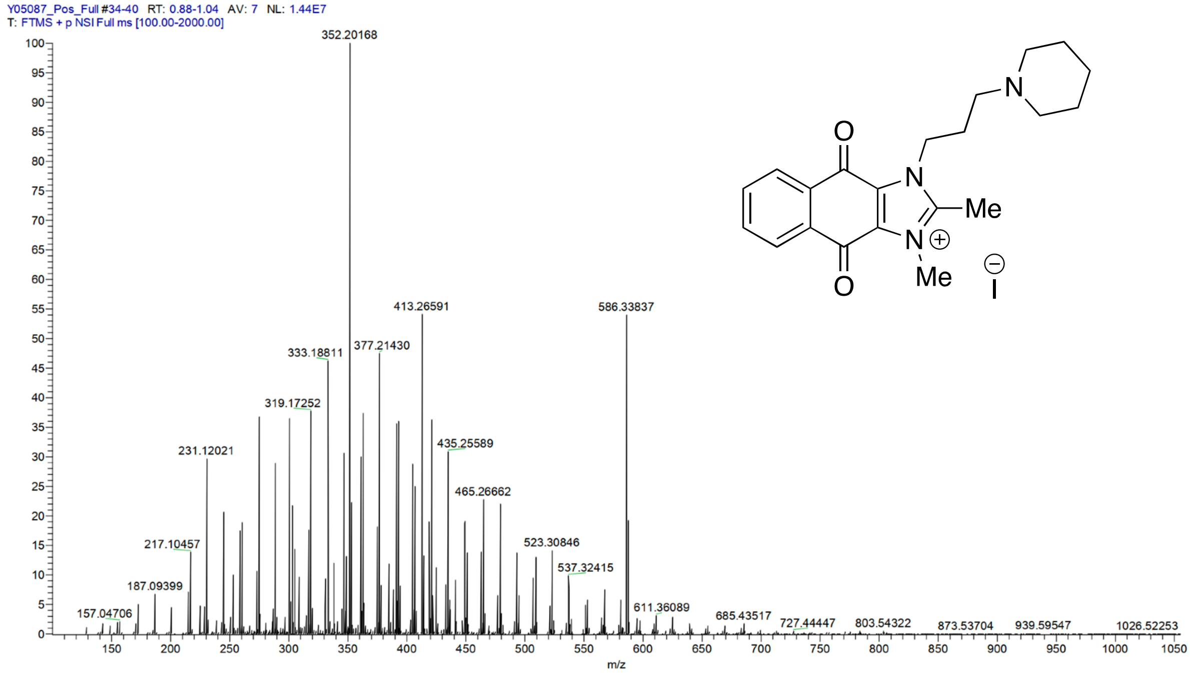

IR Spectra

IR spectrum of compound **2a**

Agilent Resolutions Pro

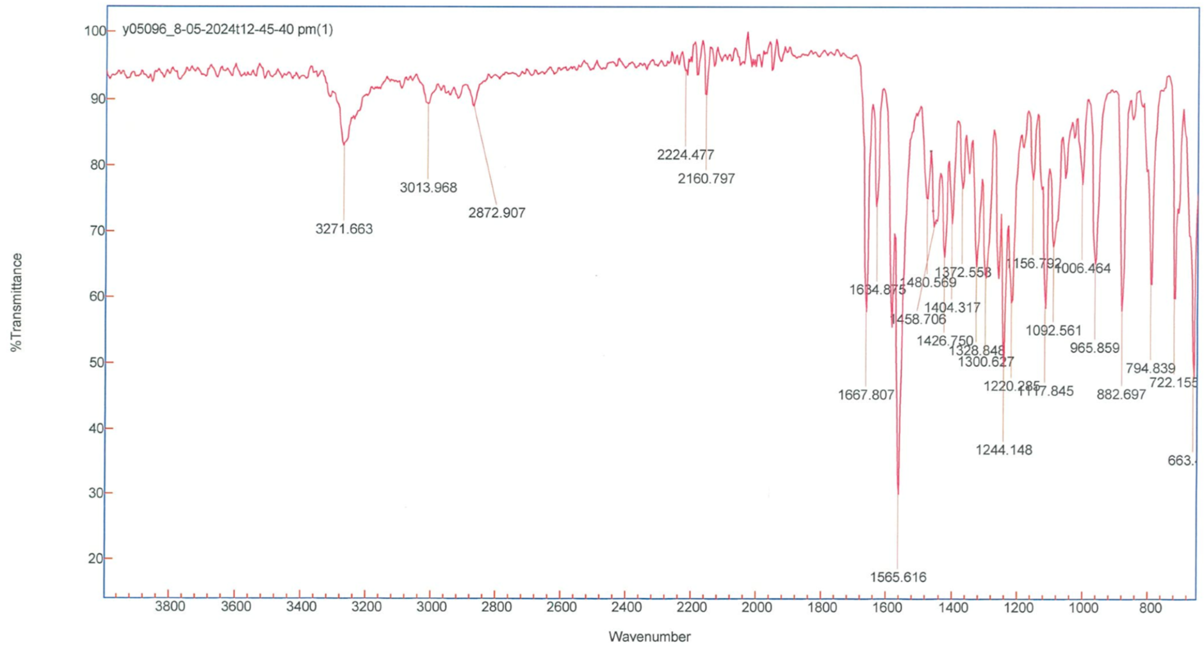

IR spectrum of compound **2b**

Agilent Resolutions Pro

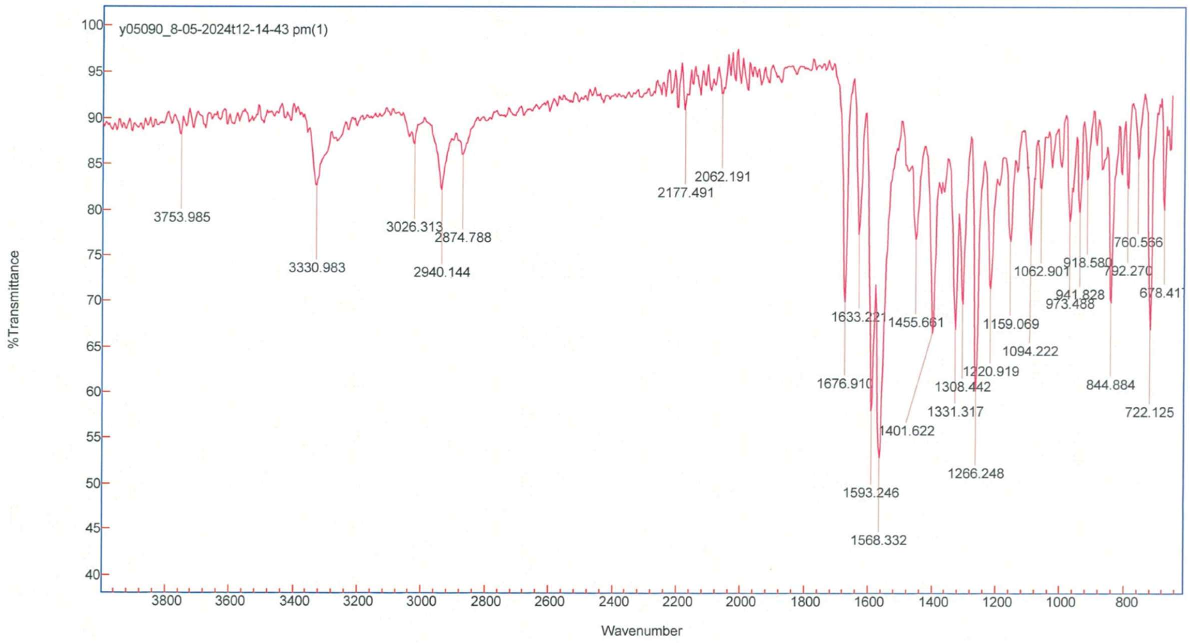

IR spectrum of compound **2c**

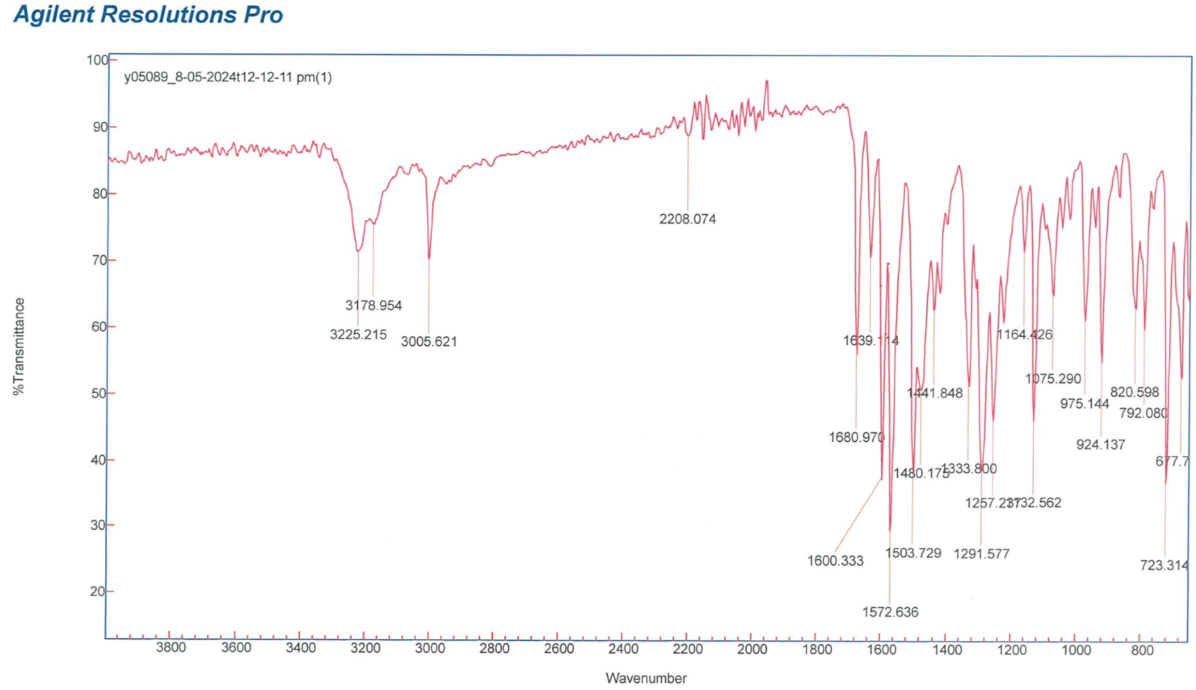

IR spectrum of compound **2d**

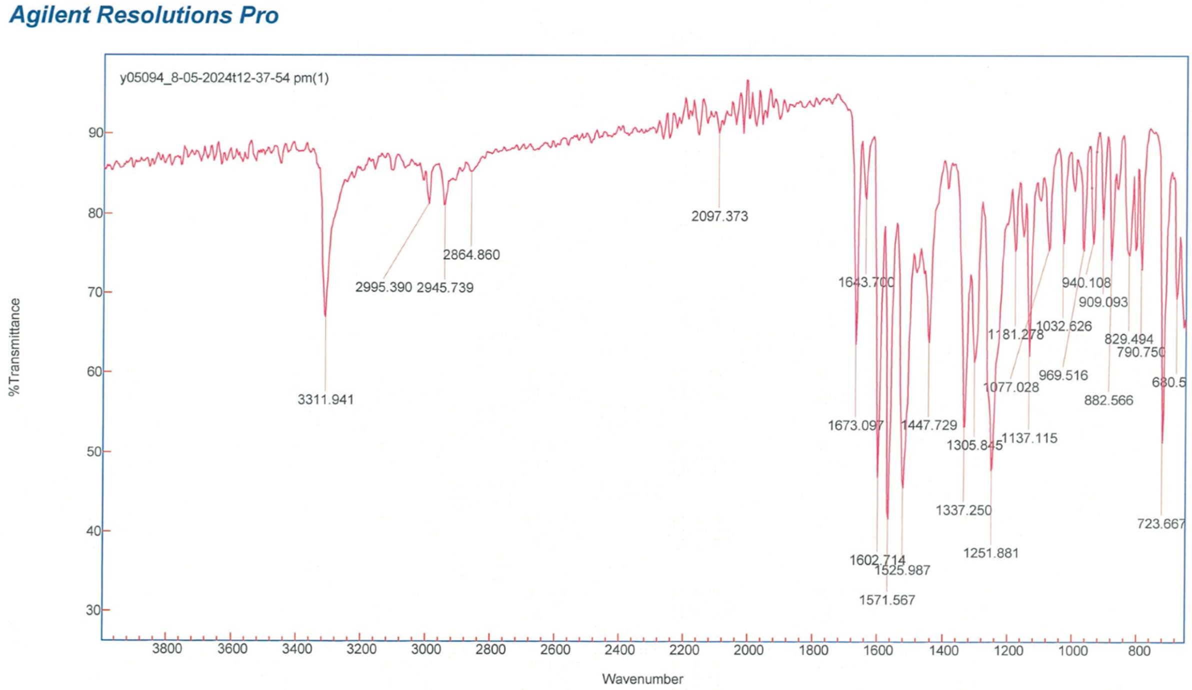

IR spectrum of compound **2e**

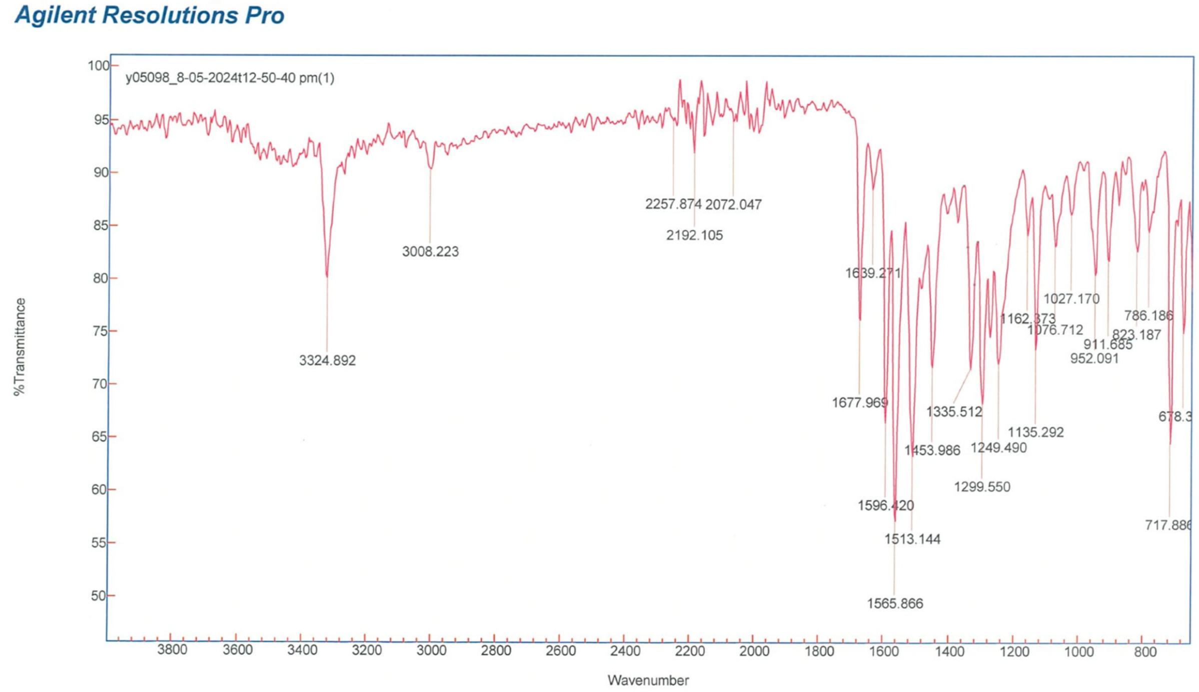

IR spectrum of compound **2f**

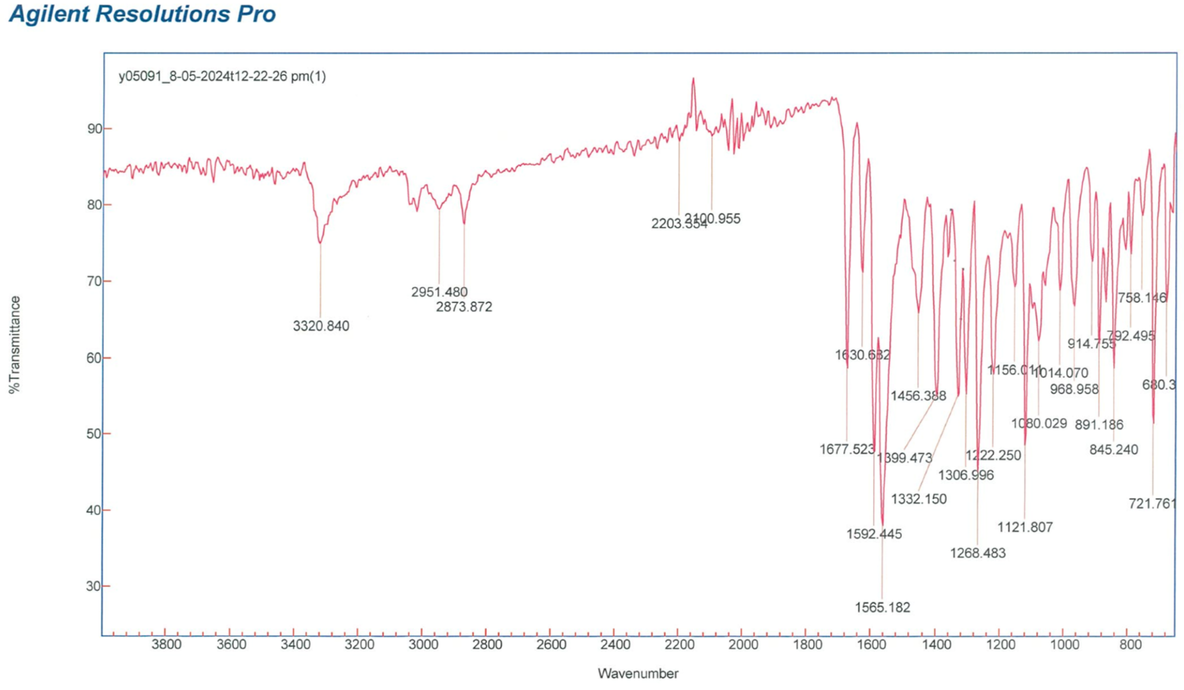

IR spectrum of compound **2g**

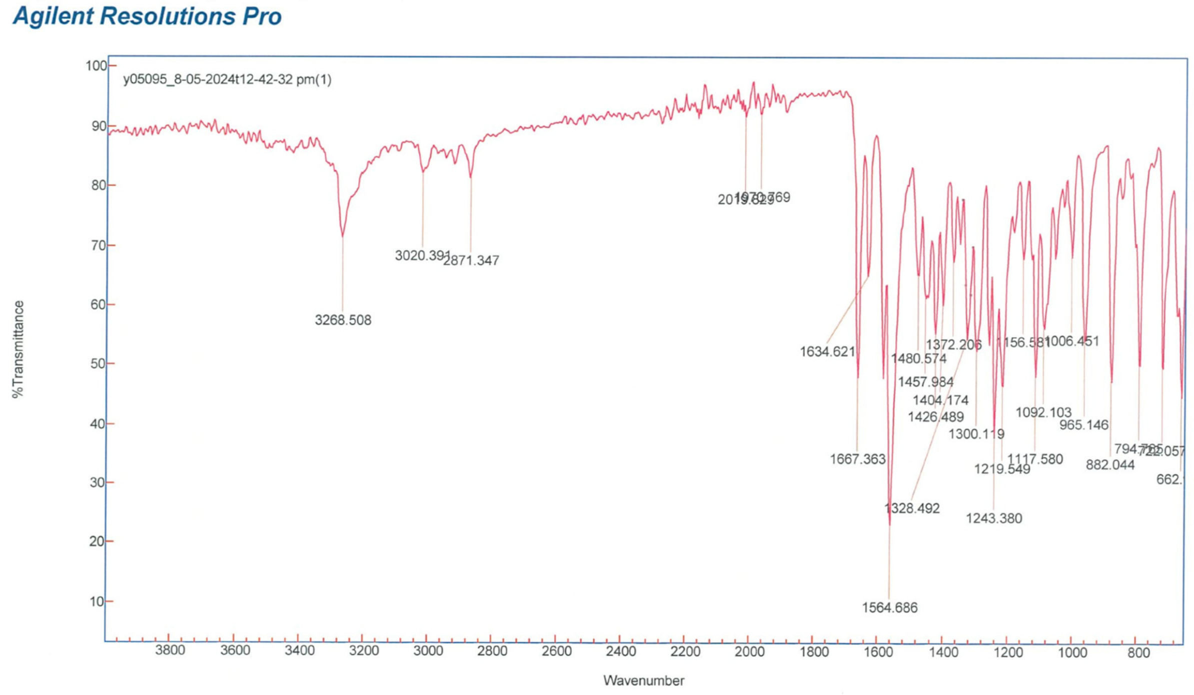

IR spectrum of compound **2h**

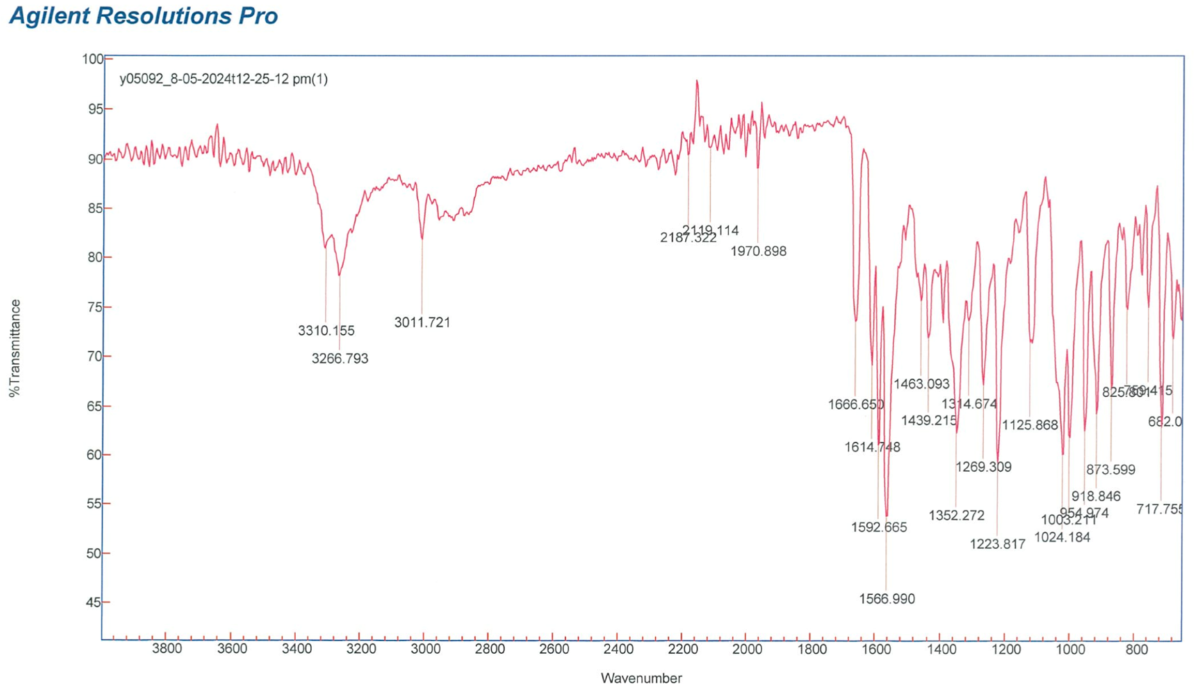

IR spectrum of compound **7a**

Agilent Resolutions Pro

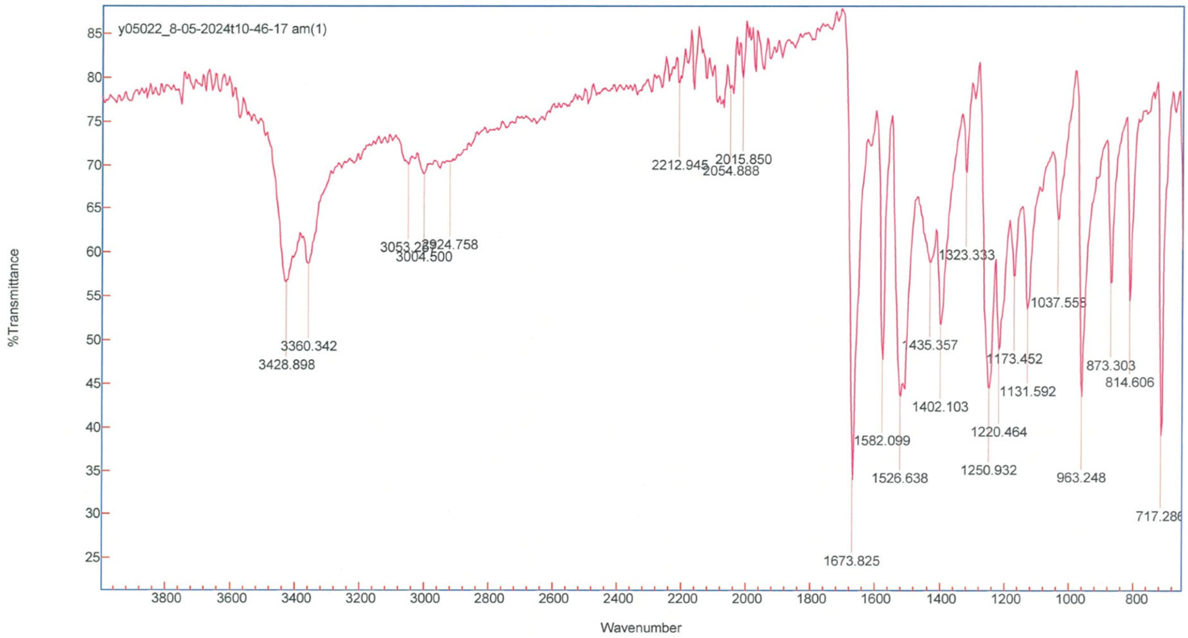

IR spectrum of compound **7b**

Agilent Resolutions Pro

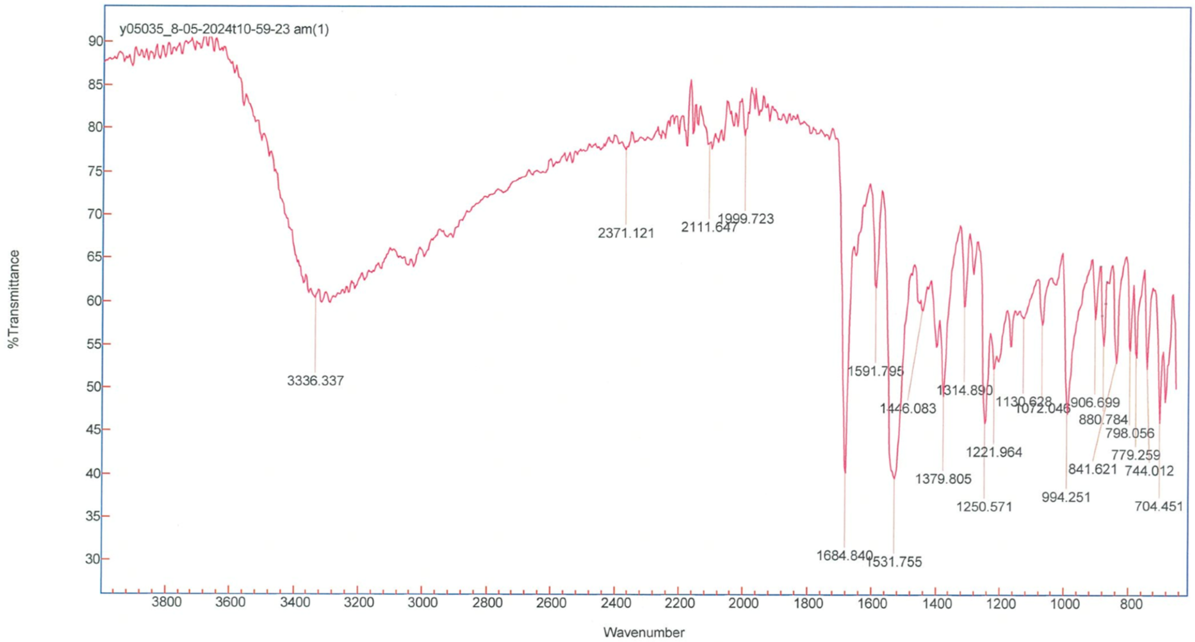

IR spectrum of compound **7c**

Agilent Resolutions Pro

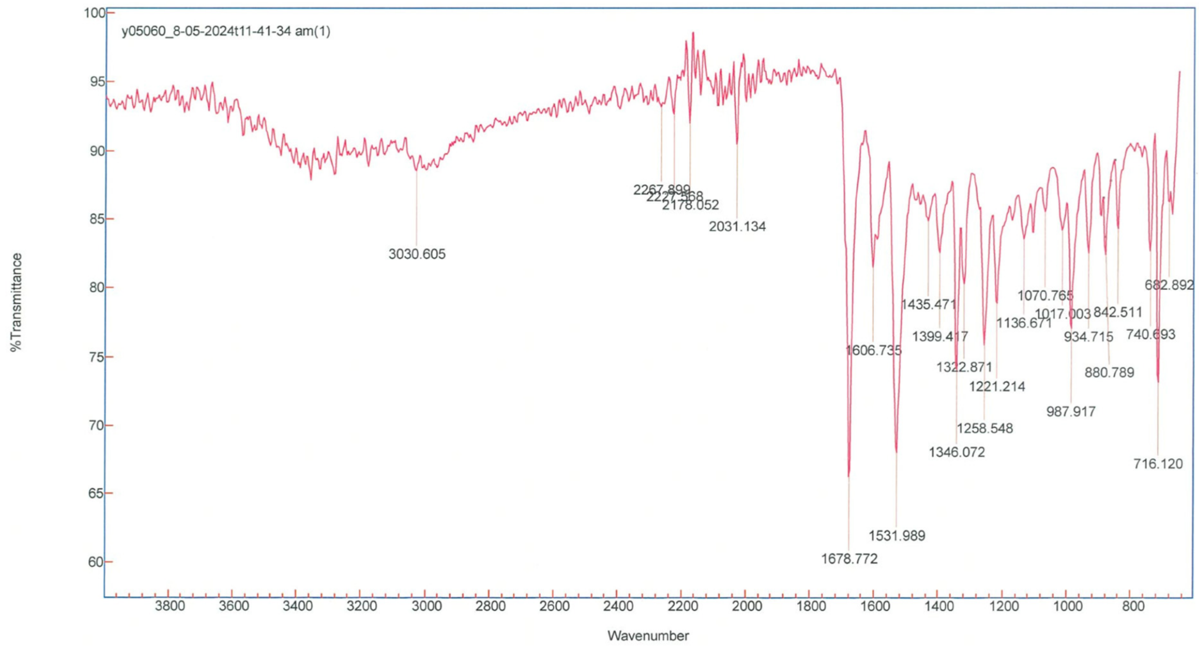

IR spectrum of compound **7d**

Agilent Resolutions Pro

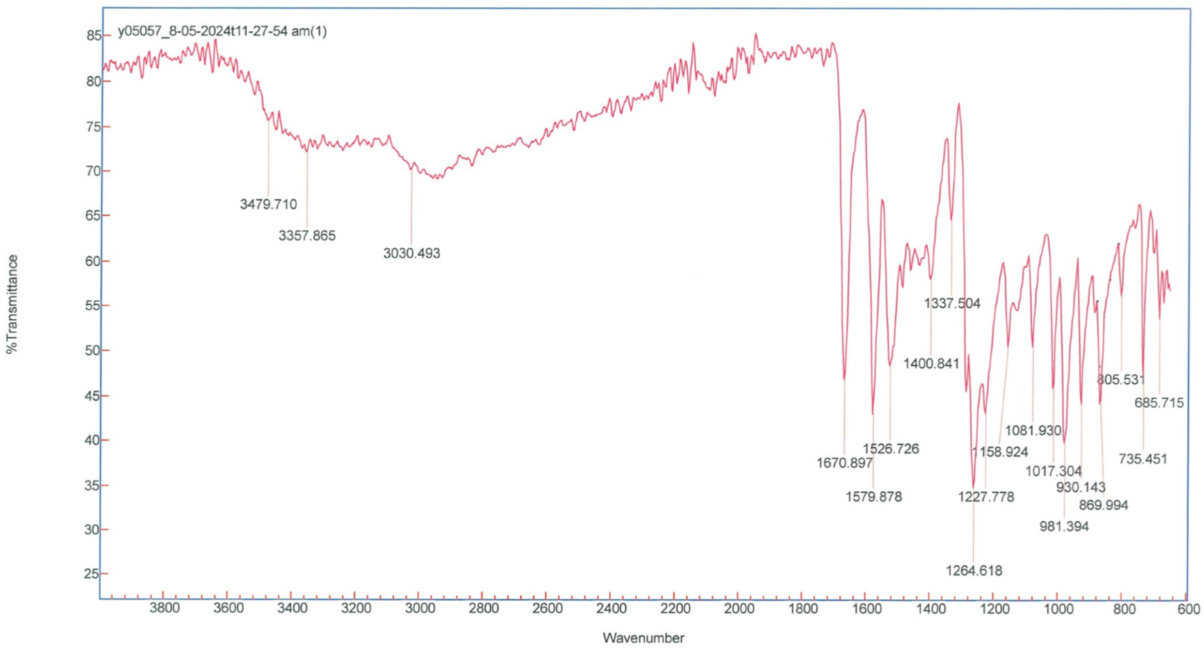

IR spectrum of compound **7e**

Agilent Resolutions Pro

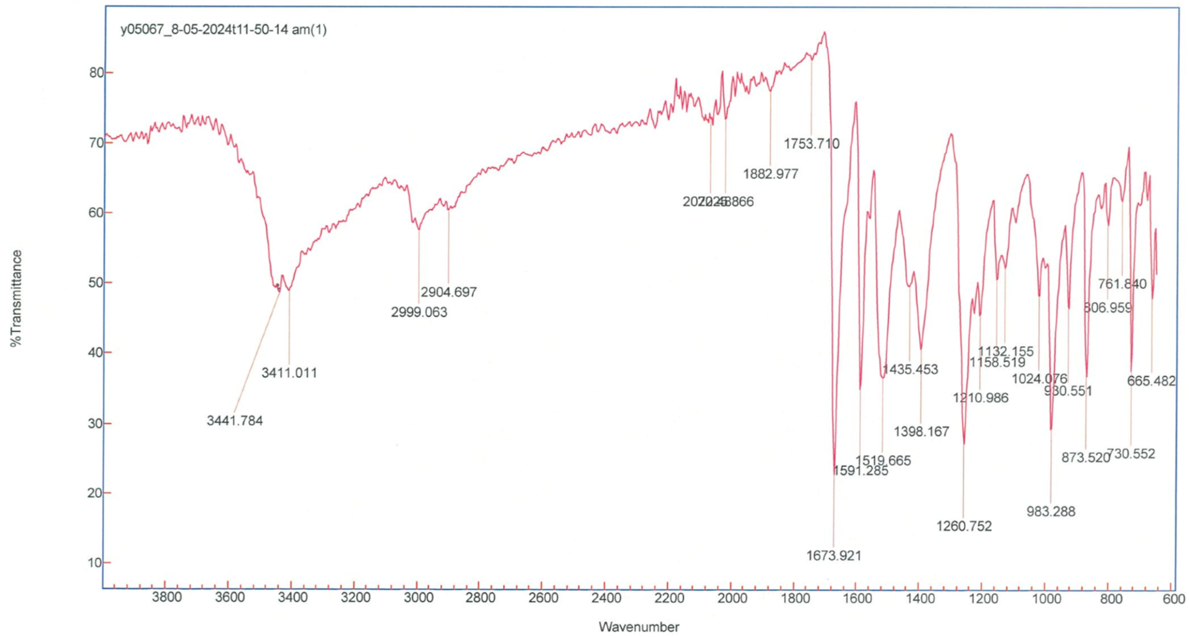

IR spectrum of compound **7f**

Agilent Resolutions Pro

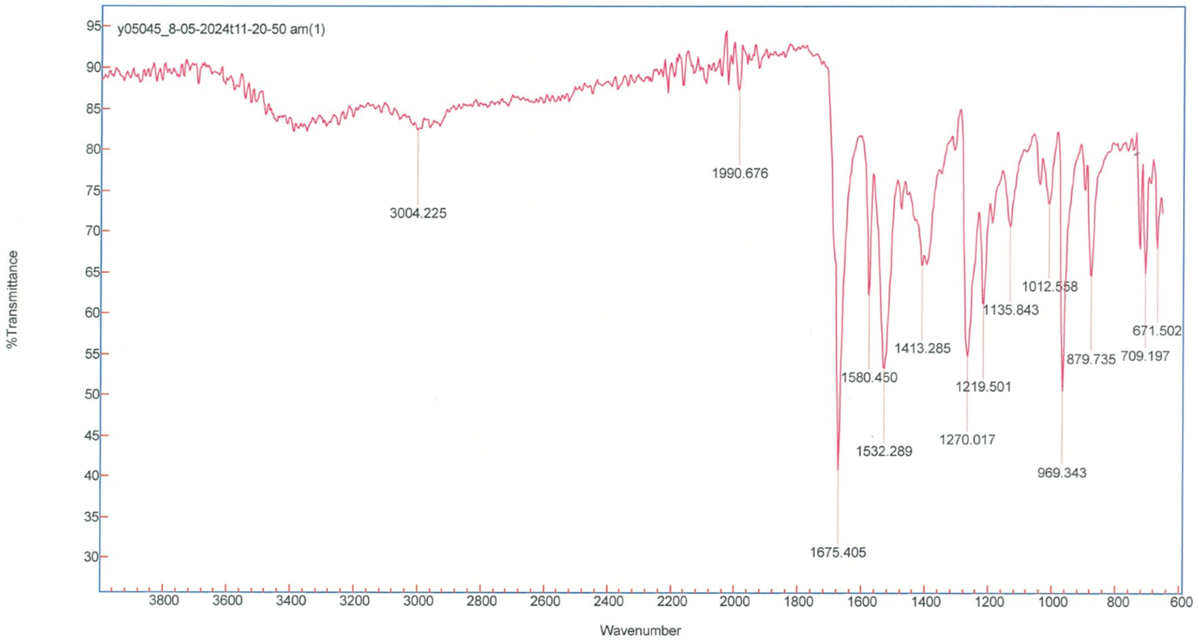

IR spectrum of compound **9**

Agilent Resolutions Pro

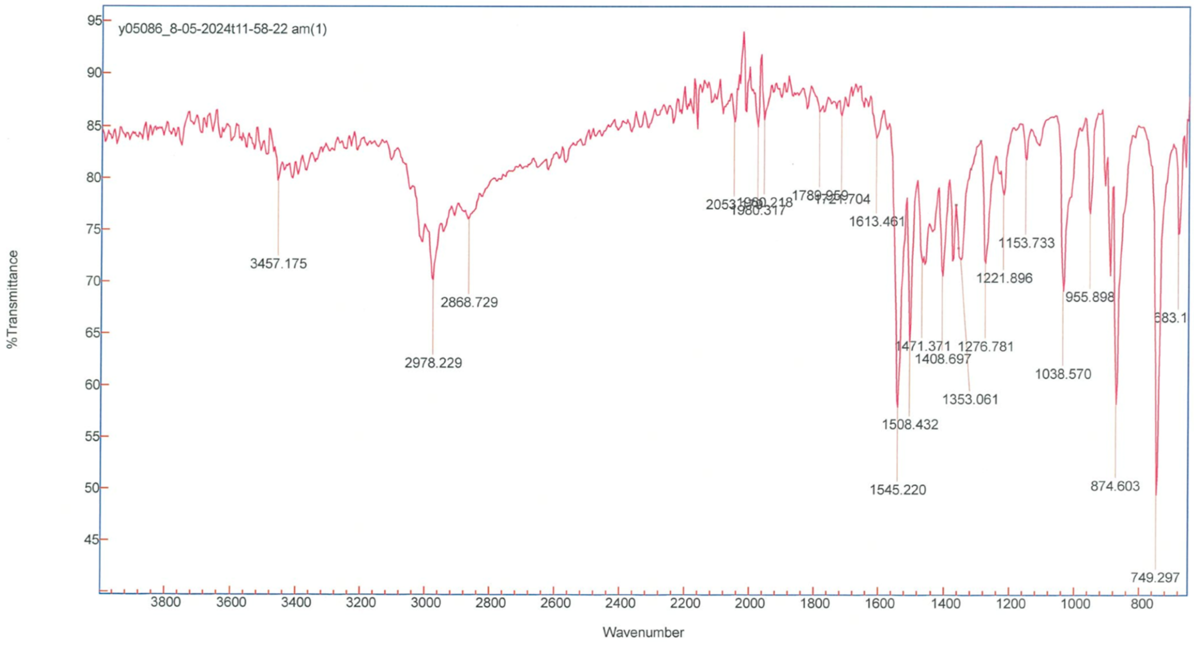

IR spectrum of compound **11**

Agilent Resolutions Pro

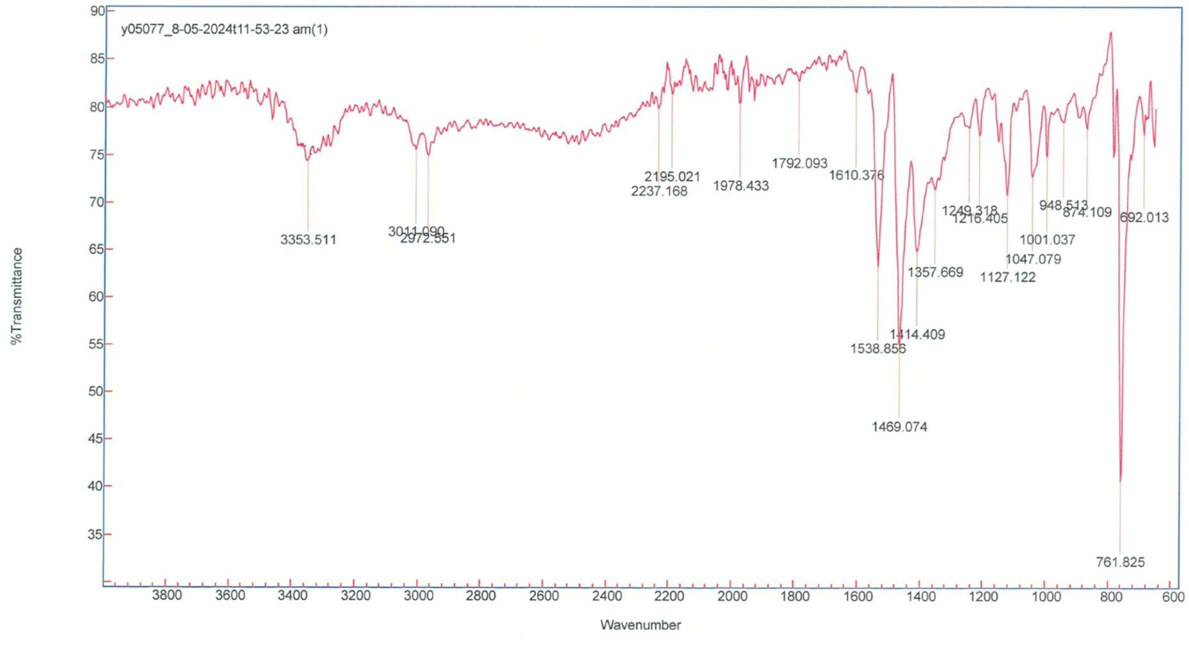

IR spectrum of compound **13**

Agilent Resolutions Pro

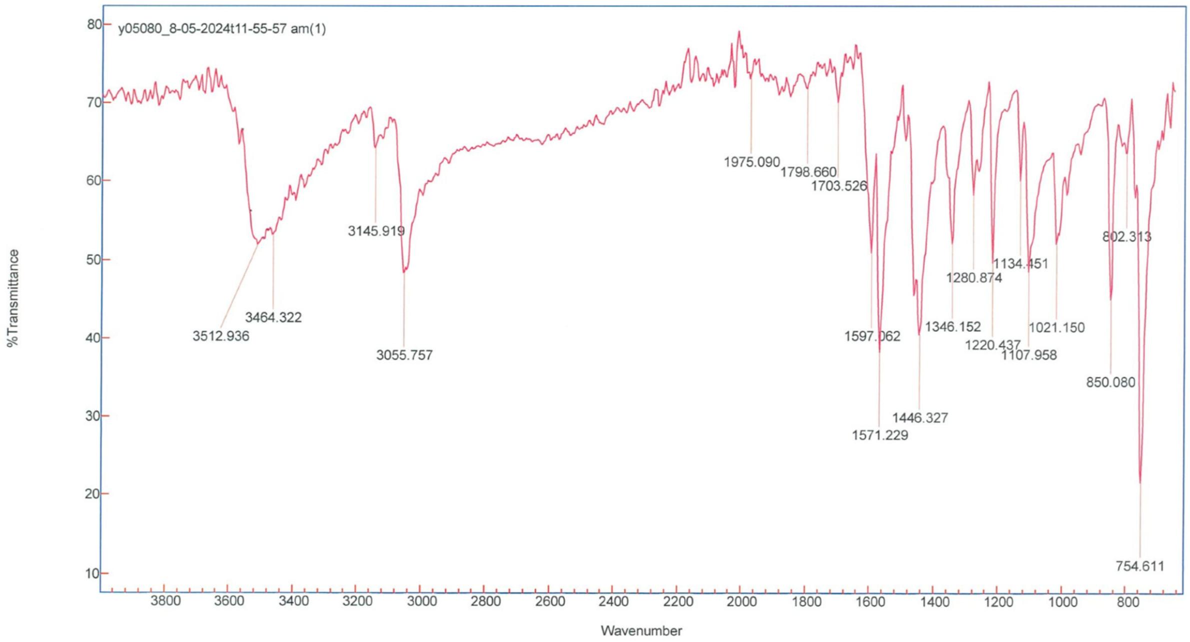

IR spectrum of compound **15**

Agilent Resolutions Pro

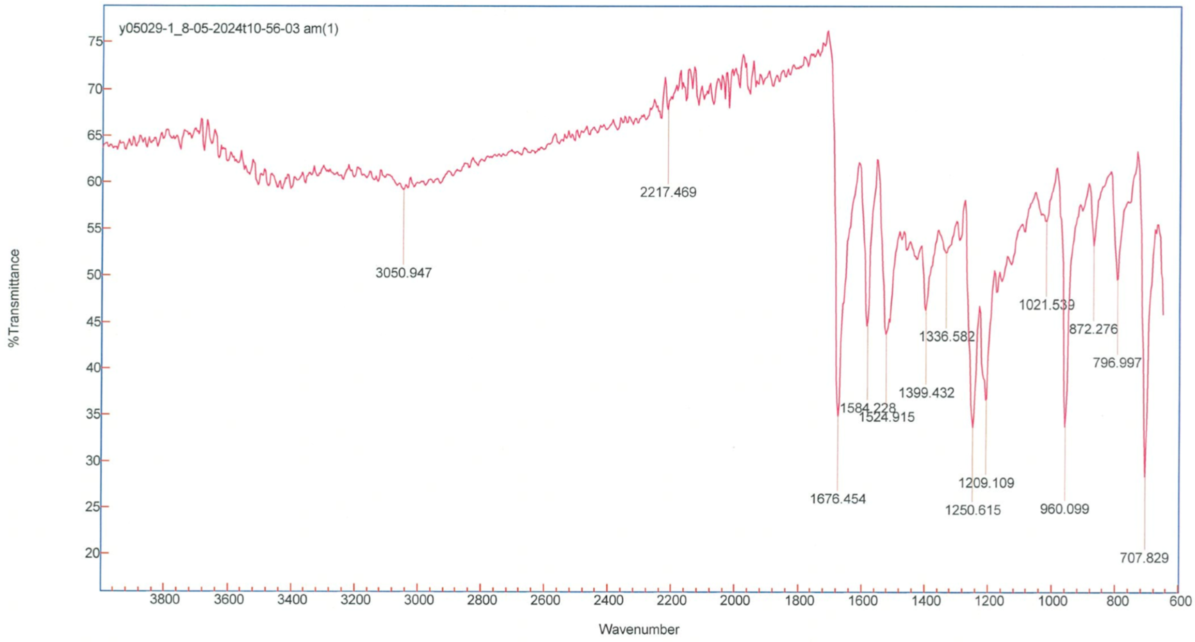

IR spectrum of compound **17a**

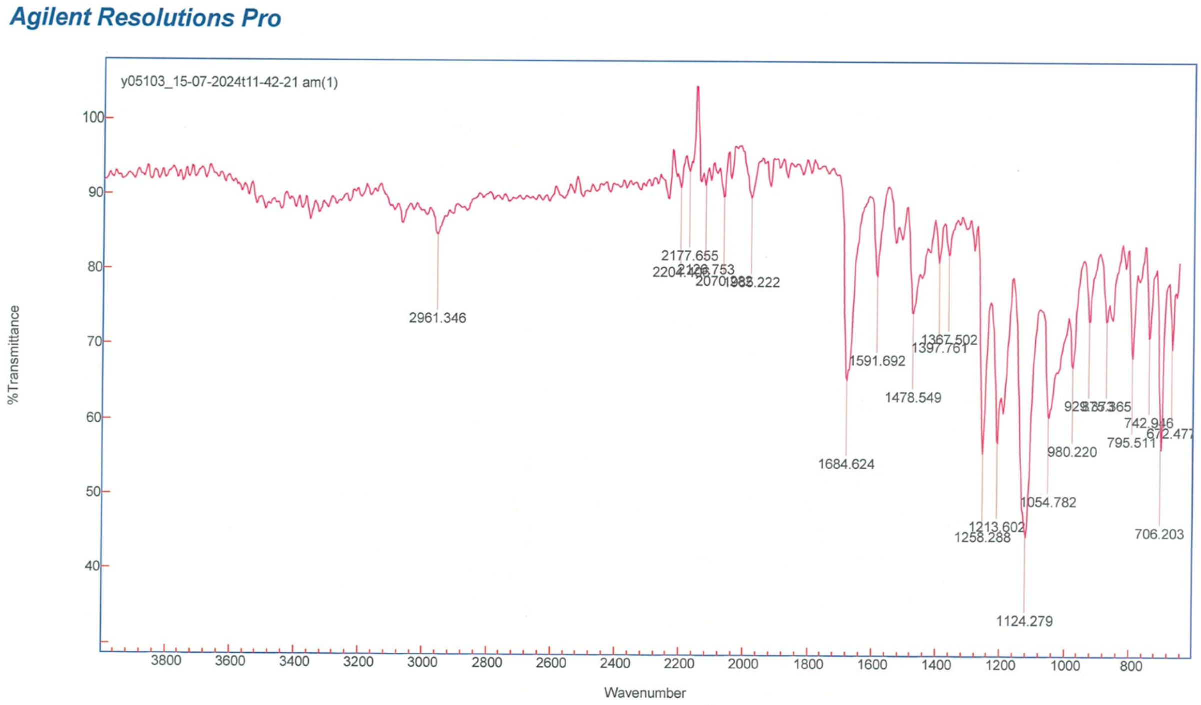

IR spectrum of compound **17b**

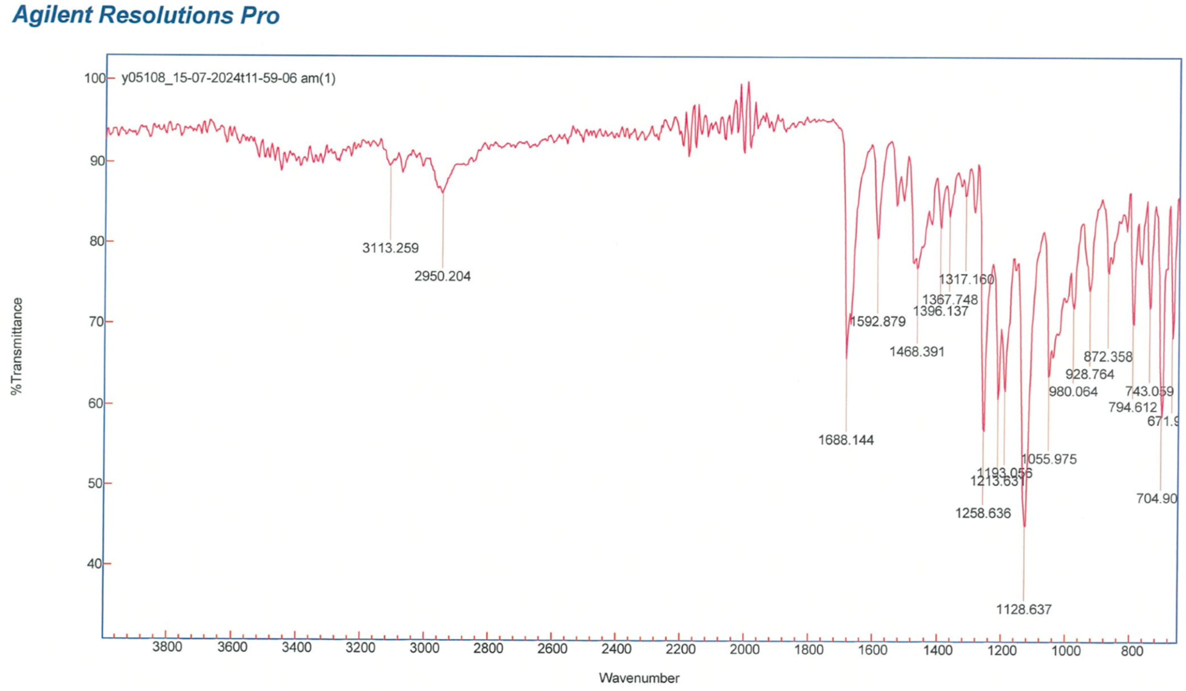

IR spectrum of compound **17c**

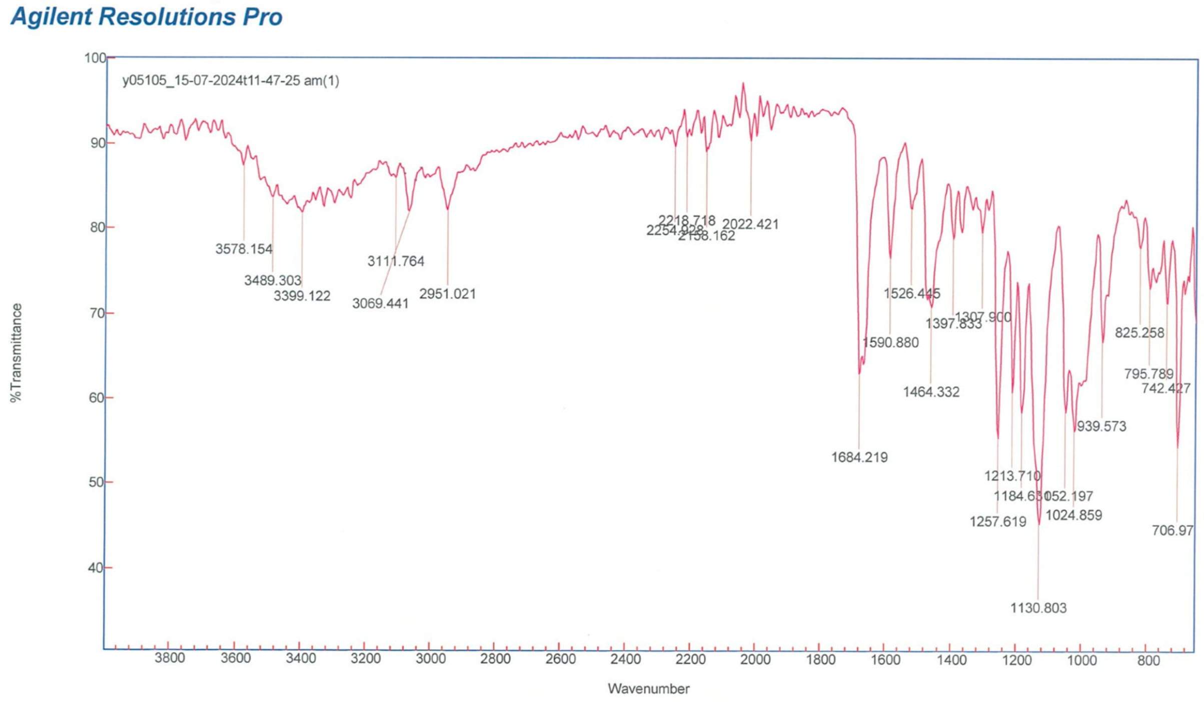

IR spectrum of compound **19**

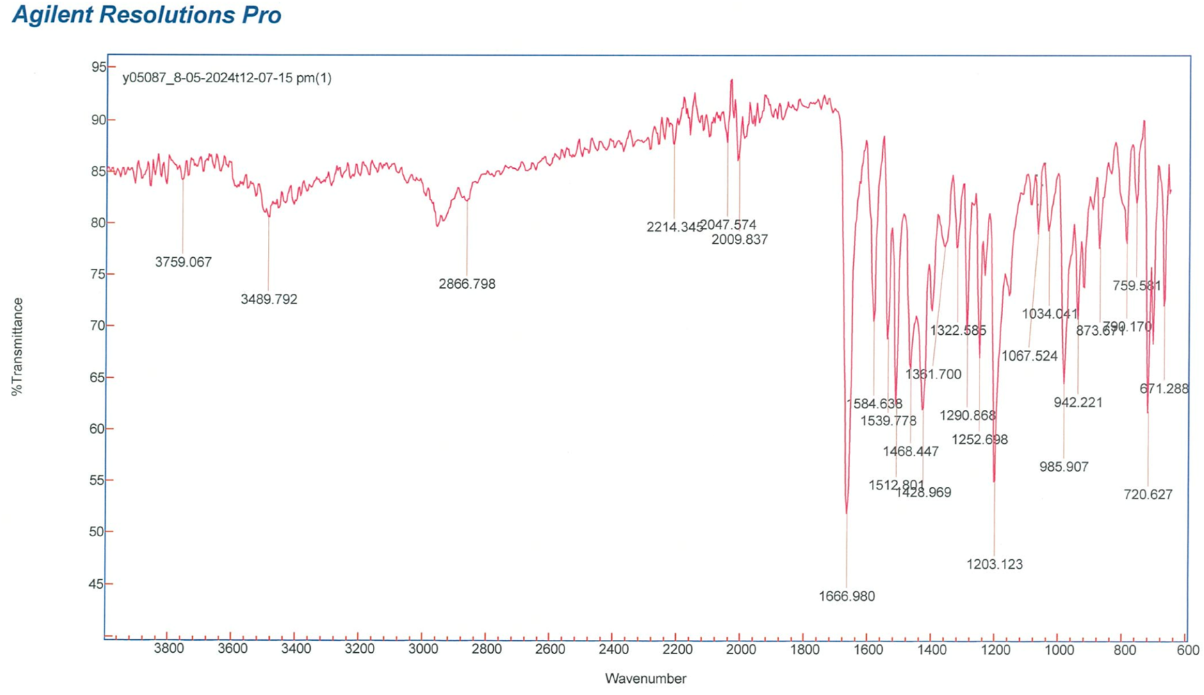

References:

1. Cheng, Y.; Jones, J. P.; Yu, T. T.; Olzomer, E. M.; Su, J.; Katen, A.; Black, D. S.; Hart-Smith, G.; Childress, E. S.; Wilkins, M. R.; Mateos, I. A.; Santos, W. L.; Hoehn, K. L.; Byrne, F. L.; Kumar, N., Design, synthesis and biological evaluation of glucose metabolism inhibitors as anticancer agents. *Bioorganic Chemistry* **2024,** *151.*
2. Abdel-Wahab, A. F.; Mahmoud, W.; Al-Harizy, R. M., Targeting glucose metabolism to suppress cancer progression: prospective of anti-glycolytic cancer therapy. *Pharmacol Res* **2019,** *150*, 104511.
3. Huang, Y.; Sun, G.; Sun, X.; Li, F.; Zhao, L.; Zhong, R.; Peng, Y., The Potential of Lonidamine in Combination with Chemotherapy and Physical Therapy in Cancer Treatment. *Cancers (Basel)* **2020,** *12* (11).
4. Rosbe, K. W.; Brann, T. W.; Holden, S. A.; Teicher, B. A.; Ill, E. F., Effect of lonidamine on the cytotoxicity of four alkylating agents in vitro. *Cancer Chemother Pharmacol* **1989,** *25*, 32-36.
5. Esteva-Font, C.; Phuan, P. W.; Anderson, M. O.; Verkman, A. S., A small molecule screen identifies selective inhibitors of urea transporter UT-A. *Chem Biol* **2013,** *20* (10), 1235-44.
6. Habtemariam, S., The Molecular Pharmacology of Phloretin: Anti-Inflammatory Mechanisms of Action. *Biomedicines* **2023,** *11* (1).
7. Qiu, H. Y.; Zhu, X.; Luo, Y. L.; Lin, H. Y.; Tang, C. Y.; Qi, J. L.; Pang, Y. J.; Yang, R. W.; Lu, G. H.; Wang, X. M.; Yang, Y. H., Identification of New Shikonin Derivatives as Antitumor Agents Targeting STAT3 SH2 Domain. *Sci Rep* **2017,** *7* (1), 2863.
8. Zhang, Q.; Liu, Q.; Zheng, S.; Liu, T.; Yang, L.; Han, X.; Lu, X., Shikonin Inhibits Tumor Growth of ESCC by suppressing PKM2 mediated Aerobic Glycolysis and STAT3 Phosphorylation. *J Cancer* **2021,** *12* (16), 4830-4840.
9. Marques, L. B.; Ottoni, F. M.; Pinto, M. C. X.; Ribeiro, J. M.; de Sousa, F. S.; Weinlich, R.; de Victo, N. C.; Kisitu, J.; Holzer, A. K.; Leist, M.; Alves, R. J.; Souza-Fagundes, E. M., Lapachol acetylglycosylation enhances its cytotoxic and pro-apoptotic activities in HL60 cells. *Toxicol In Vitro* **2020,** *65*, 104772.
10. Salustiano, E. J.; Netto, C. D.; Fernandes, R. F.; da Silva, A. J.; Bacelar, T. S.; Castro, C. P.; Buarque, C. D.; Maia, R. C.; Rumjanek, V. M.; Costa, P. R., Comparison of the cytotoxic effect of lapachol, alpha-lapachone and pentacyclic 1,4-naphthoquinones on human leukemic cells. *Invest New Drugs* **2010,** *28* (2), 139-44.
11. West, T. J.; Bi, J.; Martinez-Pena, F.; Curtis, E. J.; Gazaniga, N. R.; Mischel, P. S.; Lairson, L. L., A Cell Type Selective YM155 Prodrug Targets Receptor-Interacting Protein Kinase 2 to Induce Brain Cancer Cell Death. *J Am Chem Soc* **2023**.
12. Lucki, N. C.; Villa, G. R.; Vergani, N.; Bollong, M. J.; Beyer, B. A.; Lee, J. W.; Anglin, J. L.; Spangenberg, S. H.; Chin, E. N.; Sharma, A.; Johnson, K.; Sander, P. N.; Gordon, P.; Skirboll, S. L.; Wurdak, H.; Schultz, P. G.; Mischel, P. S.; Lairson, L. L., A cell type-selective apoptosis-inducing small molecule for the treatment of brain cancer. *Proc Natl Acad Sci U S A* **2019,** *116* (13), 6435-6440.
13. Ho, S. H.; Sim, M. Y.; Yee, W. L.; Yang, T.; Yuen, S. P.; Go, M. L., Antiproliferative, DNA intercalation and redox cycling activities of dioxonaphtho[2,3-d]imidazolium analogs of YM155: A structure-activity relationship study. *Eur J Med Chem* **2015,** *104*, 42-56.
